# The Pyruvate-Phosphate Dikinase (C_4_-*SmPPDK*) Gene From *Suaeda monoica* Enhances Photosynthesis, Carbon Assimilation, and Abiotic Stress Tolerance in a C_3_ Plant Under Elevated CO_2_ Conditions

**DOI:** 10.3389/fpls.2020.00345

**Published:** 2020-04-21

**Authors:** Sonam Yadav, Mangal Singh Rathore, Avinash Mishra

**Affiliations:** Division of Applied Phycology and Biotechnology, CSIR-Central Salt and Marine Chemicals Research Institute, Bhavnagar, India

**Keywords:** abiotic stress, C_4_ photosynthesis, carbon sequestration, halophyte, salinity, transgenic

## Abstract

A pyruvate-phosphate dikinase (C_4_-*PPDK*) gene was cloned from *Suaeda monoica*, which had a single-cell C_4_ photosynthesis pathway without Kranz anatomy and was functionally validated in a C_3_ model plant under different abiotic stress conditions in an ambient and elevated CO_2_ environment. Overexpression of *SmPPDK* promoted growth of C_3_ transgenic plants, enhancing their photosynthesis (CO_2_ assimilation) by lowering photorespiration under stress conditions. Transgenic plants also showed an improved physiological status, with higher relative water content (RWC), membrane integrity, concentration of glycine betaine, total soluble sugars, free amino acids, polyphenols and antioxidant activity, and lower electrolyte leakage, lipid peroxidation, free radical accumulation, and generation of reactive oxygen species (ROS), compared to control plants. Moreover, *SmPPDK* transgenic plants exhibited earlier flowering and higher dry biomass compared to controls. These results suggested that the C_4_-*PPDK* gene was appropriate for improvement of carbon assimilation, and it also played an important role in adaption to salinity and severe drought-induced stress. More intriguingly, an elevated CO_2_ environment alleviated the adverse effects of abiotic stress, particularly caused by drought through coordination of osmoprotectants and antioxidant defense systems. The molecular, physiological, metabolic, and biochemical indicators ameliorated the overall performance of model C_3_ plants overexpressing the C_4_-*PPDK* gene in an elevated CO_2_ environment, by lowering photorespiration metabolic processes, however, further studies are needed to confirm its precise role in C_3_ plants as protection against future climate change.

## Introduction

Global climate changes such as increasing temperature, atmospheric CO_2_ concentration and salinization, and altered precipitation are among the primary constraints on crop improvement, including biomass production and yield (Dhir, 2018). In addition, the increasing demands in feeding a fast growing population also impose pressure to enhance the productivity and quality of current crops under limited resources. Global climate changes result in desertification and salinization, with >6% of global land area and >30% of irrigated farmland being salt affected (FAO, 2011). Global CO_2_ concentrations have increased from a preindustrial level of 285 μmol mol^–1^ to the current level of 384 μmol mol^–1^, and they have been predicted to reach 936 μmol mol^–1^ by the year 2100, with an increase in global atmospheric temperature of about 6.4°C (IPCC, 2014). These stresses will likely affect the productivity of plants, influencing the sequestration and assimilation of atmospheric CO_2_ through photosynthesis.

Various studies on elevated CO_2_ concentrations have shown a close relationship between improvements to photosynthesis and increases in yield and total biomass of plants (Kellner et al., 2019; Pastore et al., 2019). Therefore, this provides an attractive approach to enhancing yield by increasing photosynthesis of a particular crop species (Zhang et al., 2014; Yadav et al., 2018). Among the three categories of photosynthesis in carbon-assimilating plants, productivity and biomass of C_4_ plants are not limited by atmospheric CO_2_; therefore, they are considered the most efficient CO_2_ sequestrators. C_4_ photosynthesis inhibits the oxygenation reaction and acts as a CO_2_ pump, increasing the intracellular CO_2_/O_2_ ratio at the active site of ribulose bisphosphate carboxylase/oxygenase (Rubisco) (Hatch, 1987; Sage, 2004). It has been well documented that C_3_ plants lose about 50% of total available CO_2_ to photorespiration compared to C_4_ plants. In addition, C_4_ photosynthesis has an advantage over that of C_3_ plants, exhibiting a higher assimilation efficiency and yield of biomass under different abiotic stress conditions, including drought, high temperature, and high light intensity, by improving water and nitrogen use efficiency (Hatch, 1987; Sage and Pearcy, 2000). Enzymes involved in the C_4_ assimilation pathway could also play a significant role in tolerance to abiotic and biotic stress (Ku et al., 2000; Gu et al., 2013). Enhancing photosynthesis efficiency in C_3_ plants using an enzyme from the C_4_ pathway could provide an alternative approach for improving productivity, biomass, and yield (Yadav and Mishra, 2019). Genera *Suaeda* and *Bienertia* have broken the dual-cell paradigm that Kranz anatomy is essential for C_4_ carbon assimilation (Freitag and Stichler, 2002; Voznesenskaya et al., 2002; Akhani et al., 2005).

*Suaeda* species belong to the family Amaranthaceae and have a unique approach to C_4_ carbon assimilation within a single chlorenchyma cell (Voznesenskaya et al., 2002, 2004), in which chloroplasts are dimorphic. A single-cell process is the most common in the nicotinamide adenine dinucleotide phosphate-dependent malic enzyme (NADP-ME) subtype of C_4_ photosynthesis species, where the chloroplasts of mesophyll cells have well-developed grana, while the chloroplasts of bundle sheath lack grana. However, single-cell C_4_ photosynthesis is not repressed by O_2_, under low CO_2_ concentration, and carbon isotope values are the same as for the Kranz type of the C_4_ plant (Voznesenskaya et al., 2002, 2004). Likewise, single-cell C_4_ plants contain dimorphic polarized chloroplasts, with a key enzyme, pyruvate phosphate dikinase (PPDK) located at the distal end of the chloroplast, while Rubisco is at the proximal end, effectively following the same differentiation as the Kranz type of anatomy (Dengler and Nelson, 1999; Sheen, 1999). As such, engineering of a single-cell C_4_ organization into C_3_ crops might provide an alternative approach to achieving Kranz type C_4_ carbon assimilation without the need to establish a dual-cell system.

PPDK (EC 2.7.9.1) is nuclear encoded, abundantly present in the chloroplasts of mesophyll cells, an important rate-limiting enzyme of the C_4_ pathway (Edwards et al., 1985; Hatch, 1987), and is regulated by light-dependent gene expression (Sheen and Bogorad, 1987). It catalyzes the interconversion of pyruvate to phosphoenolpyruvate (PEP), the acceptor of atmospheric CO_2_ in C_4_ plants, and C_4_ photosynthesis rate is correlated (*r* = 0.96) with the activity of PPDK (Edwards et al., 1985). Its structural and functional properties have been studied in the bacterium *Clostridium symbiosum* (Chastain et al., 2011). The phylogenetic distribution of PPDK is confined to selected groups of bacteria, archaea, protozoa, lower fungi, and green plants (Liapounova et al., 2006; Slamovits and Keeling, 2006). C_4_ PPDK is highly homologous to C_3_ PPDK (Agarie et al., 1997), with the regions and residues involved in catalysis also being highly conserved among bacteria (Fißlthaler et al., 1995). PPDK can also contribute to the conversion of NADH to NADPH, which is an essential coenzyme of antioxidant enzymes, and is also involved in nitrogen assimilation and synthesis of fatty acids and osmotically active compounds (Doubnerová and Ryšlavá, 2011). PPDK is critically involved during early seedling growth and seed development in both C_3_ and C_4_ plants (Chastain et al., 2006; Yu et al., 2016; Chang et al., 2017). In *Arabidopsis thaliana*, PPDK is overexpressed during leaf senescence by significantly accelerating nitrogen remobilization from leaves (Taylor et al., 2010; Wang et al., 2019). PPDK also plays a key role in host stress defense mechanisms, as it is induced by various abiotic stresses, such as drought, salinity, heavy metals, submergence, low oxygen and cold (Doubnerová and Ryšlavá, 2011; Hýsková et al., 2014; Wang et al., 2018), and biotic stress, including viral infection (Spoustová et al., 2015). In nonphotosynthetic parts of the plant, PPDK plays a significant role in the maintenance of pH and rejuvenation of citric acid intermediates, which are involved in the synthesis of amino acids (Hýsková et al., 2014). PPDK homologs have been reported from bacteria and unicellular parasitic protists, such as *Giardia lamblia*, *Trichomonas vaginalis*, and *Entamoeba histolytica*, however, they have not been found in insects or vertebrates. As such, PPDK is a potential target for antiparasitic and antimicrobial drugs, as well as C_4_-specific herbicides (Minges and Groth, 2017).

The advantages of a C_4_-based physiology are observed during stress triggered by high temperatures, drought, salinity, and elevated CO_2_ levels. PPDK is detected in C_3_ plants (Chastain et al., 2008), however, its role is undefined. Consequently, the integration of the C_4_ *PPDK* gene into C_3_ plants could be a promising approach to relegate photorespiration, resulting in the improvement of photosynthetic efficiency of C_3_ plants under climatic stress (Yadav and Mishra, 2020). In this current study, a gene that encoded PPDK was successfully introduced and expressed in a C_3_ plant (Ku et al., 2000; Fukayama et al., 2001; Gu et al., 2013).

Our previous studies suggested that halophytes were a potential resource for stress tolerance genes (Jha et al., 2011; Chaturvedi et al., 2012, 2014; Joshi et al., 2013; Singh et al., 2014b; Udawat et al., 2014, 2016; Tiwari et al., 2015) and promoters (Tiwari et al., 2014, 2016, 2019; Singh et al., 2016) that improved photosynthesis (Patel et al., 2015; Jha et al., 2019) and also provide abiotic stress tolerance (Singh et al., 2014a; Pandey et al., 2016; Mishra and Tanna, 2017; Udawat et al., 2017). Notably, the single-cell *PPDK* gene of *Suaeda* spp. was induced under elevated CO_2_ conditions (Yadav et al., 2018). In the current study, a C_4_ photosynthetic *PPDK* gene was isolated from the C_4_ halophyte *Suaeda monoica* and was functionally validated by being introduced into a C_3_ model plant, tobacco. The effects of overexpression of *SmPPDK* were studied on the phenotype, photosynthetic efficiency, and yield of transgenic tobacco plants. In addition, the synergetic effect of elevated CO_2_ stress along with other abiotic stress conditions was studied on morphology, physiology, metabolites, and the biochemical status of transgenic lines, compared to ambient conditions and wild-type (WT) plants.

## Materials and Methods

### Cloning and *in silico* Analysis of *SmPPDK* Gene

The *PPDK* gene was isolated by rapid amplification of complementary DNA (cDNA) ends (RACE) techniques, using different combinations of primers and PCR conditions (Supplementary Table S1). A full-length *PPDK* gene was cloned, sequenced (at M/s Macrogen, South Korea), and submitted to the National Center for Biotechnology information (NCBI) (accession no. MK704410).

### Genome Organization of *SmPPDK* Gene

Genomic DNA was isolated from *S. monoica* leaves using the *N*-cetyl-*N*,*N*,*N*-trimethyl ammonium bromide (CTAB) method, and the *PPDK* gene was amplified with gene-specific primers (Supplementary Table S1). Amplicons were purified, cloned, and sequenced (M/s Macrogen, South Korea). Introns and exons were analyzed using available online tools (NCBI).

### Genetic Transformation of *SmPPDK* Gene

The full-length coding sequence (CDS) of *SmPPDK* gene was amplified from cDNA using gene-specific primers containing *Kpn*I and *Xba*I restriction sites at the 5′- and 3′-ends, respectively (Supplementary Table S1), and was cloned into *pCAMBIA1301* through an intermediary plant expression vector pRT101. The resulting vector, *pCAMBIA1301:SmPPDK* was mobilized into *Agrobacterium tumefaciens* strain EHA104 and transformed to tobacco (*Nicotiana tabacum* cv. Petit Havana) plants by the standard leaf disk cocultivation method (Horsch et al., 1985). Leaf disks were cocultivated for 72 h and then transferred into direct shooting medium, followed by shoot elongation medium. Subsequently, they were transferred to rooting medium supplemented with 20 mg/L hygromycin. Rooted plants were acclimatized in soil and then transferred to a greenhouse.

Putative T0 and T1 transgenic lines were screened by germination of seeds on Murashige and Skoog (MS) medium supplemented with hygromycin (20 mg/L). Insertion of the transgene was confirmed by polymerase chain reaction (PCR) amplification using gene-specific (GSP) and vector-specific primers (Supplementary Table S1). In addition, β-glucuronidase (GUS) activity was determined in transgenic lines, along with WT and vector-control (VC) plants, using a GUS staining kit (Sigma, United States).

### Analysis of T1 Transgenic Tobacco Plants

#### Seed Germination Assay and Growth Parameter Analysis

Seeds of *SmPPDK* transgenic lines (L1, L20, L32, L33, and L40) were surface sterilized, and they germinated on MS media supplemented with hygromycin (20 mg/L), whereas seeds of WT (tobacco plants cv. Petit Havana) and VC (tobacco plants transformed with vector *pCAMBIA1301*) plants only germinated on MS basal medium without hygromycin. In addition, surface-sterilized seeds (transgenic lines, VC, and WT) were also germinated on MS medium supplemented with 200 mM NaCl, 300 mM mannitol, and two different concentrations of sucrose (1 and 2%) at 25 ± 2°C with a light cycle of 12 h light and 12 h dark. Germination assays were performed in three replicates, with each replicate containing 20 seeds. The rate of emergence/seed germination and mean germination time (MGT) were calculated (Pandey et al., 2015).

One-week-old equally sized plants, including positive T1 transgenic, VC, and WT seedlings, were arranged on MS media supplemented with 200 mM NaCl, 300 mM mannitol, and sucrose (1 or 2%) and incubated for 21 days for growth parameter (root length, shoot length, fresh weight, and dry weight) studies. In addition, uniformly sized seedlings were transferred into 1/2 strength Hoagland medium (Hoagland and Arnon, 1950) and allowed to grow at 25 ± 2°C for 45 days under a 12-/12-h light/dark cycle. Experiments were designed in two sets. In the first set, transgenic plants along with WT and VC plants were studied for stress (salinity and drought) tolerance at ambient CO_2_ (400 ppm) concentrations. Acclimatized plants were subjected to 200 mM NaCl or 10% polyethylene glycol (PEG) treatment in a hydroponic culture system for 24 h. For the second set of experiments, all plants (T1, VC, and WT) were first acclimatized at an elevated CO_2_ (900 ppm) concentration and then subjected to the stress treatments described above for 24 h in a hydroponic culture system. Subsequently, samples were harvested and processed for physiological, biochemical, and molecular analysis, with all physio-biochemical parameters of transgenic lines being compared with control plants (WT and VC). In addition, the performance of plants was compared under ambient and elevated CO_2_ stress conditions.

#### Measurement of Leaf Gas Exchange Parameter

Transgenic lines, along with WT and VC plants, treated and untreated, were grown in a plant growth chamber (Percival Scientific, United States), with the fourth youngest fully expanded leaves being used to measure photosynthetic parameters, such as net photosynthetic rate (Pn), stomatal conductance (Gs), electron transport rate (ETR), internal CO_2_ concentration (Ci), transpiration rate, and VpdL, using a portable photosynthesis system (LI-COR Biosciences Inc., Nebraska). Water use efficiency (WUE) of photosynthesis was calculated (Schulte et al., 2003). Photosynthesis-related measurements were started at 9:00 AM under the following condition: photosynthetically active radiation (PAR), 1,000 μmol m^–2^ s^–1^ photosynthesis photon flux density (PPFD); relative humidity (RH), 55–60%; leaf chamber temperature, 25°C; leaf chamber area, 2 cm; CO_2_ flowrate, 300 μm; and reference CO_2_ concentration, 400 μmol mol^–1^ for ambient CO_2_ and 900 μmol mol^–1^ for elevated CO_2_.

#### Measurement of Chlorophyll Fluorescence and Fluorescence Induction Curves

Plants were kept under actinic light (1,000 μmol^–2^ s^–1^) for 1 min, at which point photochemical quenching (qP) and quantum efficiency of photosystem II (ϕPSII) were recorded. The light-adapted chlorophyll fluorescence parameters were calculated as follows:

$$\mathrm{Photochemical}\,\mathrm{quenching}\,\left(\mathrm{qP}\right)=\frac{\left({\mathrm{Fm}}^{\prime }-\mathrm{Fs}\right)}{\left({\mathrm{Fm}}^{\prime }-{\mathrm{Fo}}^{\prime }\right)}$$

$$\mathrm{Actual}\,\mathrm{photochemical}\,\mathrm{efficiency}\,\mathrm{of}\,\mathrm{PSII}\,\left(\phi \,\mathrm{PSII}\right)=\frac{\left({\mathrm{Fm}}^{\prime }-\mathrm{Fs}\right)}{{\mathrm{Fm}}^{\prime }}$$

Where Fm′ is the maximum fluorescence in light-adapted plant, Fs is the steady state of fluorescence for light-adapted plant, and Fo′ is the initial fluorescence in light-adapted plant.

The dark-adapted maximum photochemical efficiency of PSII (Fv/Fm) was recorded on the same fourth fully expanded leaves using the following equation:

$$\mathrm{Photosynthesis}\,\mathrm{efficiency}\,\left(\mathrm{PSII}\right)\,\frac{\mathrm{Fv}}{\mathrm{Fm}}=\frac{\mathrm{Fm}-\mathrm{Fo}}{\mathrm{Fm}}$$

The dark-adapted fluorescence transient induction curve (based on OJIP curve) was recorded using a chlorophyll analyzer (Handy PEA, Hansatech Co., United Kingdom), and the resulting OJIP curve was analyzed (Strasser and Strasser, 1995). The first (O), second (J), third (I), and fourth (P) phases were recorded with fluorescence intensities at 0.02, 0.3, 2, and 30 ms, respectively.

#### Measurement of Photosynthetic Pigments

About 100 mg fresh weight (FW) of leaves (control and treated plants) was homogenized in 1 ml chilled *N*,*N*-dimethylformamide (DMF) for 2–3 h in the dark. Absorbance was measured at 665, 647, and 461 nm using a UV–visible spectrophotometer (SpectramaxPlus 384, Molecular Devices, United States). Total chlorophyll (a + b), chlorophyll a (Chl a), chlorophyll b (Chl b), and carotenoids were calculated as per following equation (Inskeep and Bloom, 1985; Chamovitz et al., 1993):

$$\mathrm{Chl}\,a=12.70\,\left(A_{665}\right)-2.79\,\left(A_{647}\right)$$

$$\mathrm{Chl}\,b=20.70\,\left(A_{647}\right)-4.62\,\left(A_{665}\right)$$

$$\mathrm{Total}\,\mathrm{Chl}\,\left(a+b\right)=17.90\,\left(A_{647}\right)+8.08\,\left(A_{665}\right)$$

$$\mathrm{Carotenoids}=\left[A_{461}-\left(0.046\times A_{665}\right)\right]\times 4$$

#### Measurement of Relative Water Contain

The weight of fresh leaves of equal size about 2 cm (from control and treated plants) was recorded (FW); leaves were immersed in deionized water at room temperature for overnight (16 h). Samples were dried at room temperature (by keeping on blotting papers), and turgid weight (TW) was recorded; after that, samples were dried at 65°C (in an oven) for 48 h, and dry weight (DW) was calculated. The relative water content (RWC) was calculated as percentage by following equation:

$$\mathrm{Relative}\,\mathrm{water}\,\mathrm{contain}\,\left(\mathrm{RWC}\right)\%=\left(\frac{\mathrm{FW}-\mathrm{DW}}{\mathrm{TW}-\mathrm{DW}}\right)\times 100$$

#### Measurement of Electrolyte Leakage and Membrane Stability Index

Fresh leaf samples of control and treated plants were washed with deionized water to remove surface-staking electrolytes. Simultaneously, leaves were cut into equal size and immersed into 10 ml of deionized water in closed vials and incubated in gentle shaking at room temperature for 4 h. Electrical conductivity was recorded (*L*_t_) with a conductivity meter (Seven Easy, Mettler Toledo AG8603, Switzerland), and after that, sample vials were incubated at 99°C for 20 min and cooled at room temperature, and final electrical conductivity (*L*_0_) was recorded (Lutts et al., 1996).

For membrane stability index (MSI), fresh leaf samples were washed thoroughly with deionized water, and then, the experiment was conducted into two sets. In one set, leaves were immersed in 10 ml of deionized water and incubated at 40°C for 30 min. In another set, leavers were immersed in 10 ml of water and incubated at 99°C for 10 min. Electrical conductivity was measured for both the sets of vials (Sairam, 1994). The percentage of electrolyte leakage and MSI were calculated using the following equation:

$$\mathrm{Electrolyte}\,\mathrm{leakage}\,\left(\mathrm{EL}\right)=\left(\frac{L_{t}}{L_{0}}\right)\times 100$$

$$\mathrm{Membrane}\,\mathrm{stability}\,\mathrm{iindex}\,\left(\mathrm{MSI}\right)=100\times 1-\left(\frac{L_{1}}{L_{2}}\right)$$

#### Quantification of PPDK Enzyme and Activity Assay

About 500 mg of fresh leaf samples of control and treated plants was homogenized with 2 ml of extraction buffer [50 mM HEPES–KOH and 10 mM of dithiothreitol (DTT); pH 7.5] for the extraction of PPDK enzyme (Yadav et al., 2018). The enzyme was quantified (Bradford, 1976), and the activity was measured at 340 nm in the forward direction by coupling the production of PEP to NADH via PEPC and malate dehydrogenase (MDH) (Alla and Hassan, 2012). Briefly, a reaction mixture containing 25 mM HEPES–KOH (pH 8.0), 8 mM MgSO_4_, 10 mM DTT, 10 mM NaHCO_3_, 2 mM pyruvate, 5 mM (NH_4_)_2_SO_4_, 1 mM glucose-6-phosphate, 1 mM ATP, 2.5 mM KPO_4_, 0.2 mM NADH, 0.5 U PEPC, and 2 U MDH was prepared. The activity of PPDK was started by adding 0.2 ml of crude enzyme extract in 1 ml freshly prepared reaction mixture, allowed to react at 25°C, and absorbance was read at 340 nm (*ε* = 6,221 μl μmol^–1^ cm^–1^). The PPDK specific activity was expressed as nanomoles of enzyme per gram of FW, and enzyme activity was expressed as kat (the release of 1 mol of NAD s^–1^) mg^–1^ protein.

#### Measurement of Malondialdehyde and Hydrogen Peroxide Contents

About 500 mg of fresh leaf samples was extracted with 1 ml of ice-cold 0.1% of trichloroacetic acid (TCA) reagent followed by centrifugation at 10,000 *g* for 10 min at 4°C, and the supernatant (extract) was collected for malondialdehyde (MDA) and hydrogen peroxide content. Lipid peroxidation was studied by estimating MDA content (Hodges et al., 1999). Extracts were divided into two sets: first, 0.5 ml of the extract was mixed with 2 ml of TBA (0.65% *w*/*v*, in 20% TCA); second, 0.5 ml of the extract was mixed with 20% TCA. Samples were incubated at 95°C for 30 min and, after cooling, centrifuged at 10,000 *g* for 5 min. The absorbance was measured at 440, 532, and 600 nm, respectively, and MDA content was quantified using the following equation:

$$A=\left({\mathrm{Abs}}_{532+\mathrm{TBA}}-{\mathrm{Abs}}_{600+\mathrm{TBA}}\right)-\left({\mathrm{Abs}}_{532-\mathrm{TBA}}-{\mathrm{Abs}}_{600-\mathrm{TBA}}\right)$$

$$B=\left({\mathrm{Abs}}_{440+\mathrm{TBA}}-{\mathrm{Abs}}_{600-\mathrm{TBA}}\right)\times 0.0571$$

$$\mathrm{MDA}\,\left({\mathrm{nmolg}}^{-1}\right)=\frac{\left[\left(\frac{A-B}{157000}\right)\times 10^{6}\right]}{\mathrm{Weight}\,\mathrm{of}\,\mathrm{tissue}\,\mathrm{in}\,\mathrm{gram}}$$

For the estimation of hydrogen peroxide (H_2_O_2_) content, 0.25 ml of the extract was mixed with reaction buffer [0.25 mM FeSO_4_, 0.25 mM (NH_4_)_2_SO_4_, 25 mM H_2_SO_4_, 1.25 mM xylenol orange, and 1 mM sorbitol], followed by incubation at room temperature for 30 min. The absorbance was measured at 560 nm to quantified hydrogen peroxide content with a known concentration of H_2_O_2_, which was used as a standard (He et al., 2000).

#### Quantification of Glycine Betaine

Glycine betaine (GB), a quaternary ammonium compound, was quantified with some modifications (Grieve and Grattan, 1983). Glycine betaine was extracted from 1,000 mg of leaves in 20 ml of deionized H_2_O (for 24 h at 25°C). Extracts were filtered, diluted 1:1 with 2 N H_2_SO_4_, and incubated for 1 h at ice. Subsequently, 200 μl of freshly prepared cold KI–I_2_ (15.7 g of iodine and 20 g of KI in 100 ml of H_2_O) was added in the sample and incubated 15–20 min and then centrifuged at 10,000 *g* for 10 min at 4°C. After that, periodide crystals were separated from an acid medium, dissolved in 3 ml of 1,2-dichloroethane, and incubated for 2.5 h in dark. Simultaneously, a standard curve was prepared with glycine betaine (100 μg/ml), and absorbance was recorded at 365 nm.

#### Measurement of Antioxidant Enzyme Activity

About 500 mg of the leaf samples was used for the extraction of protein. Samples were homogenized in 2 ml of extraction buffer, containing 50 mM potassium phosphate buffer (pH 7.0), 1 mM EDTA, 0.05% (*w*/*v*) Triton X-100, 5% (*w*/*v*) polyvinylpolypyrrolidone (PVPP), 1 mM phenylmethylsulfonyl fluoride (PMSF), and 2 mM DTT. The extracted protein was quantified (Bradford, 1976) and further used for the estimation of catalase (CAT), ascorbate peroxidase (APX), superoxide dismutase (SOD), and glutathione reductase (GR) activities.

Catalase (1.11.1.6) activity was quantified by observing the disappearance of H_2_O_2_ (Miyagawa et al., 2000). The activity of catalase was started by adding 20 μg of enzyme extract in 3 ml of the reaction mixture that contained 50 mM of phosphate buffer (pH 7.0) and 10 mM of H_2_O_2_. The absorbance was measured at 240 nm for every 30 s up to 3 min in a UV–visible spectrophotometer (Molecular Devices, United States). The change in absorbance at 240 nm was recorded, and activity of CAT was measured using *ϕ* = 43.6 mM^–1^ cm^–1^ as extinction coefficient and expressed as U/mg (Patterson et al., 1984).

Ascorbate peroxidase (APX: EC 1.11.1.11) was estimated by observing the oxidation of ascorbate (Nakano and Asada, 1981). The activity of APX in the reaction was started by adding 40–60 μg of enzyme extract in 3 ml of reaction mixture containing 50 mM phosphate buffer (pH 7.0), 0.5 mM ascorbic acid, and 0.1 mM H_2_O_2_, and absorbance was recorded at 290 nm for 2 min to determine the specific activity of APX (*ϕ* = 2.8 mM^–1^ cm^–1^).

For glutathione reductase (GR: EC 1.8.1.7) activity, 50 μl of the enzyme extract was added in the reaction mixture containing 50 mM of phosphate buffer (pH 7.5), 1 mM EDTA, 1 mM glutathione oxidized (GSSG), 0.75 mM 5,5-dithio-bis-(2-nitrobenzoic acid) (DTNB), and 0.1 mM NADPH. Absorbance was recorded at 412 nm for 2 min, and specific activity was estimated (*ε* = 26.6 mM^–1^ cm^–1^).

For the measurement of SOD activity, the inhibition of photochemical reduction in nitro-blue tetrazolium (NBT) was used (Beyer and Fridovich, 1987). For the enzyme assay, 3 ml of reaction mixture containing 50 mM phosphate buffer (pH 7.8), 9.9 mM L-methionine, 58 μM NBT, 0.025% Triton-X, and 2.4 μM riboflavin was prepared. Simultaneously, 0.1 ml of the enzyme extract was added into the reaction mixture and allowed to react for 10 min at an illuminated light along with blank (containing reaction mix without enzyme extract). The reaction was stopped by incubating the sample (and blank) in the dark for 15 min. The amount of enzyme required for 50% inhibition of the photochemical reduction in NBT was recorded at 560 nm and represented as 1 U of SOD activity.

#### Measurements of Total Sugar, Reducing Sugar, Starch, Free Amino Acid, and Polyphenol Content

About 1,000 mg of leaves was extracted in 80% ethanol. The extract was reacted with 2 ml of anthrone reagent (0.2% *w*/*v* anthrone in 95% of H_2_SO_4_) for total sugar and 3 ml of freshly prepared 3,5-dinitrosalicylic acid (DNS) reagent for reducing sugar estimation, respectively. Subsequently, samples were incubated at a boiling water bath for 5 min and cooled at room temperature, and the absorbance was measured at 630 and 540 nm, respectively. For starch estimation, the left pellet was digested with 52% of perchloric acid and centrifuged at 10,000 *g* for 10 min. The digested product was reacted with 3 ml of anthrone reagent, incubated at a boiling water bath for 10 min, and the absorbance was recorded at 630 nm (Ghose, 1987). For estimation of free amino acid and polyphenol, about 0.2 ml of plant extracts was reacted with Ninhydrin reagent (Sugano et al., 1975) and Folin–Ciocalteu (FC) reagent (Chandler and Dodds, 1983), respectively. The reaction mixture was incubated at a boiling water bath, and absorbance was measured at 540 and 650 nm for free amino acid and polyphenol, respectively. Known concentration of glucose, glycine, and catechol were used as a standard for the estimation of sugars (including starch), amino acids, and polyphenol, respectively.

#### Gene Expression Analysis

Total RNA was isolated from transgenic lines, WT and VC plants (Chomczynski and Sacchi, 1987), converted to cDNA using reverse transcriptase according to the manufacturer’s (Promega, United States) instructions. Primer *NtActine* was used as an internal reference to normalize the gene expression of selected genes studied by real-time quantitative PCR (Supplementary Table S1). Differential transcript expression was studied for key genes involved in the metabolism of reactive oxygen species (ROS) (*NtCAT*, *NtAPX*, *NtGR*, and *NtSOD*), and photorespiration [hydroxypyruvate reductase (NtHPR) and glycolate oxidase (*NtGO*)]. The real-time quantitative PCR (RT-qPCR) was performed in a CFX 96TM Real-Time system (Bio-Rad, United States) with 1× SYBR green PCR reaction mix (Supplementary Table S1). The relative expression of the transcript (2^–ΔΔ*Ct*^) was compared with the corresponding control (Livak and Schmittgen, 2001).

#### Analysis of Metabolites

Total metabolites were extracted from transgenic lines, WT, and VC plants and analyzed by gas chromatography–mass spectrometry (Lisec et al., 2006). Leaf samples (1,000 mg) were made fine powder using liquid N_2_ and extracted with 100% ethanol (ice cold). Simultaneously, 30 μl of adonitol (0.2 mg/ml) was added as an internal reference, vortexed for 20 s, incubated at 70°C, after which it was sonicated for 10 min at room temperature. Samples were centrifuged at 10,000 *g* for 10 min, clear supernatant was collected, 325 μl chloroform was added, followed by addition of 700 μl of water; samples were mixed thoroughly and centrifuged at 10,000 *g* for 5 min. The aqueous phase was collected and dried in a vacuum concentrator. About 60 μl of methoxyamine pyridine was added and incubated at 37°C for 2 h. Finally, 130 μl of *N*-methyl-*N*-(trimethylsilyl) trifluoroacetamide (MSTFA) was added, incubated for 30 min at 37°C, and used for the gas chromatography–mass spectrometry (GC-MS) analysis (GC-2010 Plus Shimadzu, Model GCMS TQ8040). The chromatographic parameters were as follows: column, RTX-5MS (diphenyl dimethyl polysiloxane, 30.0 m × 0.25 mm); injection, split injection; injection volume, 1 μl; split ratio, 50.0; and temperature, 250°C (Tanna et al., 2018). The initial temperature was 80°C with a hold time of 2 min; after that, temperature was raised up to 315°C with a rate of 10°C/min and held for 15 min (Tanna et al., 2019). As a carrier, helium gas was used with a flowrate of 2 ml/min, and the total processing time was 40–50 min. Mass spectra were recorded, metabolites were identified by matching peaks with available NIST library, and concentration was measured with respect to internal reference peak area and expressed as μg/g FW.

#### *In situ* Localization of Peroxide (H_2_O_2_) and Superoxide Radicals (O_2_^–^)

*In situ* analysis of peroxide and superoxide radicals were analyzed by histochemical staining with 3,3-diaminobenzidine (DAB) and NBT, respectively (Shi et al., 2010). Both DAB and NBT staining solutions were prepared with a concentration of 1 mg/ml in 10 mM phosphate buffer (pH 3.8 for DAB and pH 7.8 for NBT). Same age of leaves, harvested from transgenic lines, WT, and VC were immersed in their corresponding staining solutions and incubated for 2 h in the dark at room temperature. Later on, they were exposed to light until the brown and blue color visualized on the leaf surface. The staining solutions were removed, samples were bleached with aqueous ethanol (70%), and photographs were taken.

### Statistical Analysis

All experiments were performed in triplicate, and each replicate contained 15 plants. Data were given as mean ± standard error (SE), and statistical significance was determined by analysis of variance (ANOVA), using Tukey’s honestly significant difference (HSD) test. Significant differences were considered at *p* < 0.05 and designated by different letters. Correlation and multivariate analyses were performed using Pearson’s correlation matrix, regression analysis, and principal component analysis, using SYSTAT and Sigma Plot software.

## Results

### Genomic Organization and Genetic Transformation of the *PPDK* Gene

**FIGURE 1:**
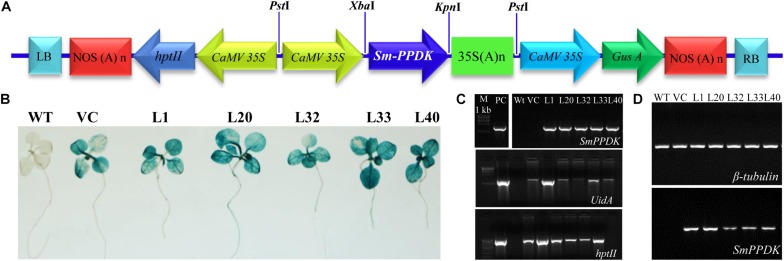
Vector construct and conformation of transgene in transgenic lines. Gene construct: **(A)** *pCAMBIA1301:SmPPDK*, **(B)** histochemical β-glucuronidase (GUS) assay of selected transgenic lines, **(C)** molecular confirmation of transgenic lines by PCR, and **(D)** semiquantitative reverse transcriptase PCR.

A 4,434 bp *PPDK* gene sequence, obtained from genomic DNA of *S. monoica*, comprised six introns flanked by seven exons (Supplementary Figure S1). A full-length cDNA (2,862 bp) of the *SmPPDK* gene (Supplementary Figure S2) was cloned in *pCAMBIA1301* under the control of the cauliflower mosaic virus (CaMV) 35S promoter (*pCAMBIA1301:SmPPDK*) (Figure 1A) and transformed to tobacco, resulting in 50 transgenic lines (T0) being obtained. The presence of the transgene (*PPDK*, *hptII*, and *uidA*) was confirmed in T0-transgenic lines by PCR amplification, using gene-specific primers (Supplementary Table S1). In addition, T0 transgenic seedlings and controls [wild-type (WT) and vector control (VC)] were subjected to histochemical GUS assays. Based on molecular confirmation by PCR and histochemical GUS assays, five transgenic lines L1, L20, L32, L33, and L40 were selected for further analysis. For these, T1 transgenic lines were further confirmed by histochemical GUS assay (Figure 1B) and PCR (Figure 1C), in comparison with the controls. Semiquantitative reverse transcriptase PCR, in which *β-tubulin* was used as an internal reference gene for normalization, demonstrated that there was overexpression of the *SmPPDK* gene in selected T1 transgenic lines (Figure 1D). Quantitative real-time-based analysis confirmed that all transgenic lines (L1, L20, L32, L33, and L40) contained a single integration event (Supplementary Figure S3). Scanning electron microscopy (field emission) of leaves of transgenic and control plants confirmed that overexpression of *SmPPDK* did not affect the ultrastructure of mesophyll cells (Supplementary Figure S4).

### Overexpression of the *SmPPDK* Gene Improved Seed Germination and Seedling Growth Under Stress Conditions

Transgenic seed showed significantly higher germination (mean germination time and seed germination rate) compared to WT and VC seeds under different stress conditions (Supplementary Figures S5, S6). They also took less time to germinate (higher seed germination rate) under different abiotic stress conditions, compared to WT and VC seeds. Similarly, improved growth (Supplementary Figure S7), including increased fresh weight, dry weight, shoot length, and root length (Supplementary Figure S8), and morphological changes (Supplementary Figure S9) were observed in transgenic seedlings under different abiotic stress conditions, compared to WT and VC plants.

### *SmPPDK* Overexpressing Transgenic Lines Showed Improved Photosynthesis and Physiology Under Different Abiotic Stress Conditions at Ambient and Elevated CO_2_ Level

A cumulative principal component analyses revealed a complex biplot comprised of seven observations (WT, VC, and transgenic lines, L1, L20, L32, L33, and L40) and 114 variables (Supplementary Figure S10). Principal components analysis (PCA) and heat map showed the differential physiological and biochemical response of transgenic lines (L1, L20, L32, L33, and L40) and control plants (WT and VC) under normal and stress conditions (Supplementary Figures S10, S11).

#### CO_2_ Assimilation Indicators

**FIGURE 2:**
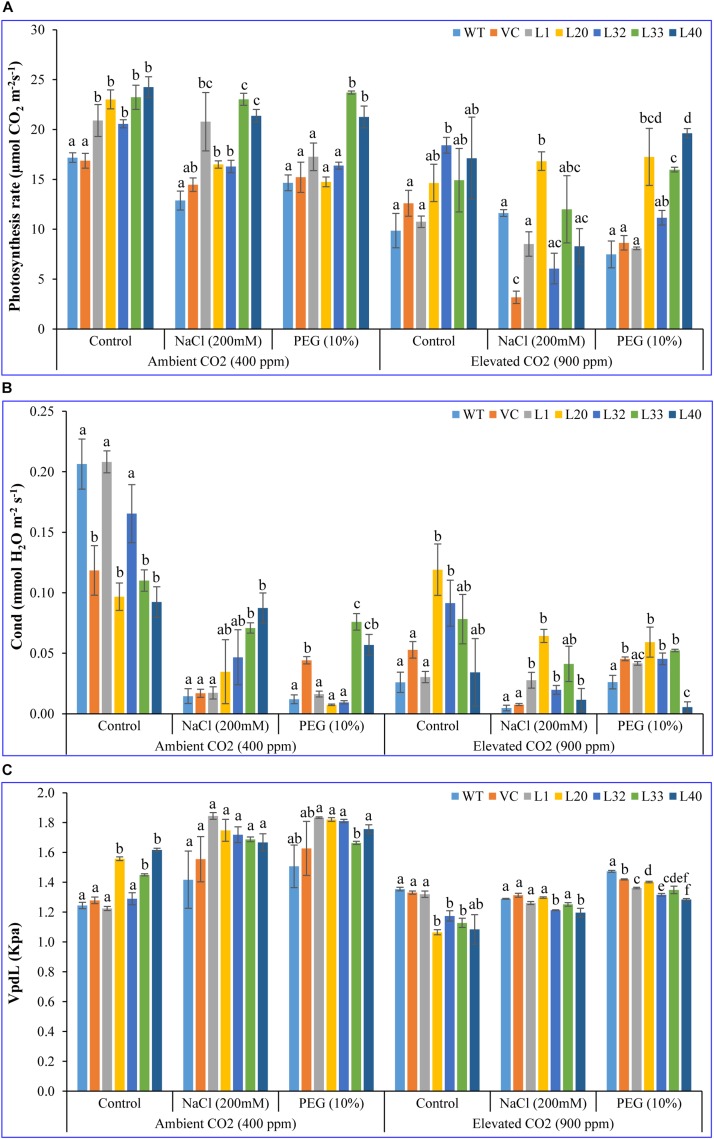
Analysis of the photosynthesis parameters (net photosynthesis, stomatal conductance, and VpdL) of transgenic lines. **(A)** Net photosynthesis, **(B)** stomatal conductance, and **(C)** VpdL of T1 transgenic lines (L1, L20, L32, L33, and L40), wild type (WT), and vector control (VC) were analyzed under salinity (200 mM NaCl) and osmotic [10% polyethylene glycol (PEG)] stress condition grown in ambient and elevated CO_2_ environment. Bars represent means ± SE, and values with different letters are significant at *P* < 0.05.

**FIGURE 3:**
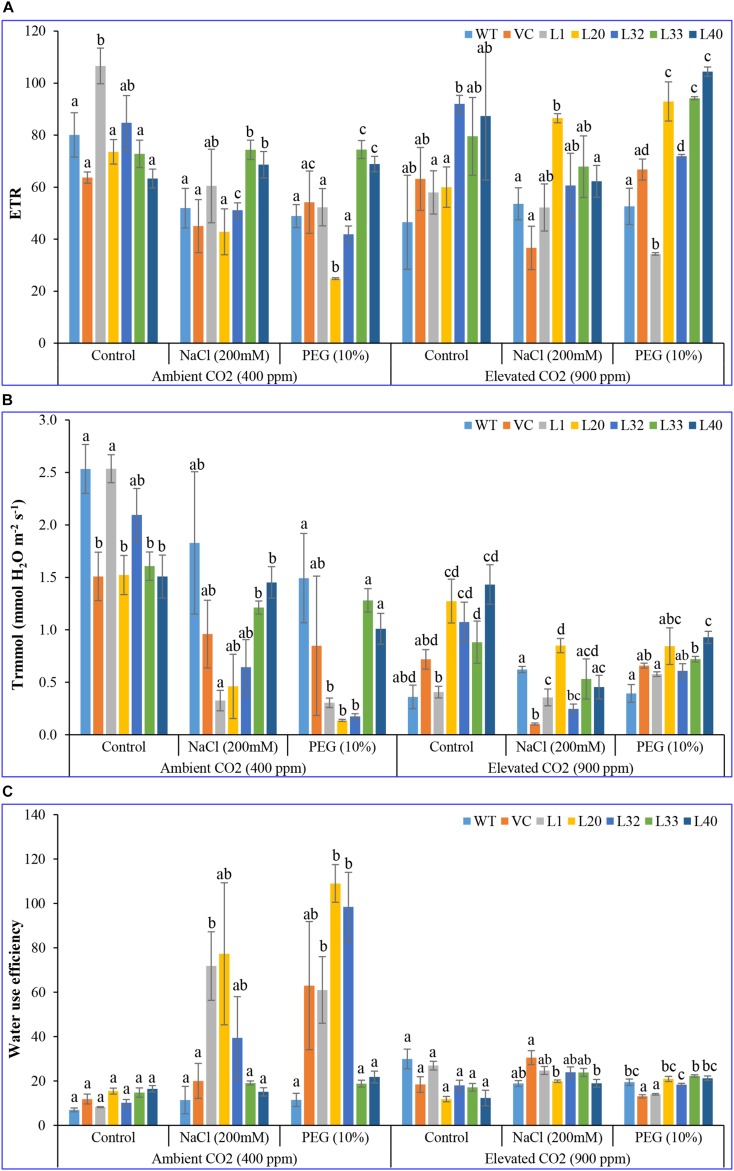
Analysis of the photosynthesis parameters (electron transport rate, transpiration rate, and water use efficiency) of transgenic lines. **(A)** Electron transport rate, **(B)** transpiration rate, and **(C)** water use efficiency of T1 transgenic lines (L1, L20, L32, L33, and L40), wild type (WT), and vector control (VC) were analyzed under salinity (200 mM NaCl) and osmotic [10% polyethylene glycol (PEG)] stress condition grown in ambient and elevated CO_2_ environment. Bars represent means ± SE, and values with different letters are significant at *P* < 0.05.

Net photosynthesis rate (Pn) of transgenic lines (16–24 μmol CO_2_ m^–2^ s^–1^) was higher than that of control (WT and VC) plants (12–17 μmol CO_2_ m^–2^ s^–1^) under control and stress conditions at ambient CO_2_ levels. Although Pn of all plants (transgenic lines along with WT and VC) significantly lowered under elevated CO_2_ conditions; the level of decrease was less for the transgenic lines and was still higher (14–23 μmol CO_2_ m^–2^ s^–1^) compared to control (WT and VC) plants (3–12 μmol CO_2_ m^–2^ s^–1^) under control and stress conditions (Figure 2A). Under ambient CO_2_ conditions, no significant difference in stomatal conductance (Gr; 0.1–0.2 mmol H_2_O m^–2^ s^–1^) was observed between transgenic lines and control plants (WT and VC), whereas under NaCl and drought stress, transgenic lines showed higher stomatal conductance (0.02–0.8 and 0.01–0.08 mmol H_2_O m^–2^ s^–1^, respectively) than WT and VC plants (0.01–0.05 mmol H_2_O m^–2^ s^–1^). At elevated CO_2_ levels, stomatal conductance significantly decreased under control conditions compared to ambient CO_2_, however, transgenic lines maintained a higher level of conductance under control and stress conditions (0.02–0.1 mmol H_2_O m^–2^ s^–1^) than that of WT and VC plants (0.005–0.05 mmol H_2_O m^–2^ s^–1^) (Figure 2B). At ambient CO_2_ levels, the vapor pressure difference between leaf intercellular air spaces and the atmosphere (VpdL) was significantly higher in transgenic lines (1.3–1.8 kPa) compared to WT and VC plants (1.2–1.6 kPa), under control and stress conditions. Under elevated CO_2_ levels, VpdL decreased in control and stress conditions, however, no significant difference was observed between transgenic lines and control (WT and VC) plants (Figure 2C). The trend of photosynthetic electron transport rate (ETR) complemented that of photosynthetic rate and was similar in both controlled and stressed plants under ambient and elevated CO_2_ conditions (Figure 3A). At ambient CO_2_ levels, the transpiration rate (Tr) was higher in all plants (transgenic lines along with WT and VC) under control conditions (1.5–2.5 mmol H_2_O m^–2^ s^–1^), compared to elevated CO_2_ conditions (0.4–1.5 mmol H_2_O m^–2^ s^–1^), and no significant difference was noticed between transgenic lines and control (WT and VC) plants (Figure 3B), although, under elevated CO_2_ levels, transgenic lines showed a higher Tr in control and drought stress conditions than that of WT and VC plants. In addition, WUE of photosynthesis was higher in transgenic lines under salt (15–77) and drought (19–109) stress at ambient CO_2_ levels, compared to WT (12) and VC (20–63) plants (Figure 3C). However, at elevated CO_2_ levels, WUE decreased in all plants, however, the degree of decline was lower in transgenic lines than that of control (WT and VC) plants. Overall, these results indicated that tobacco transgenic for *SmPPDK* had better photosynthesis characteristics than WT and VC plants, under both ambient and elevated CO_2_ stress conditions.

#### Chlorophyll a Fluorescence Parameters and Fluorescence Induction Curve

**FIGURE 4:**
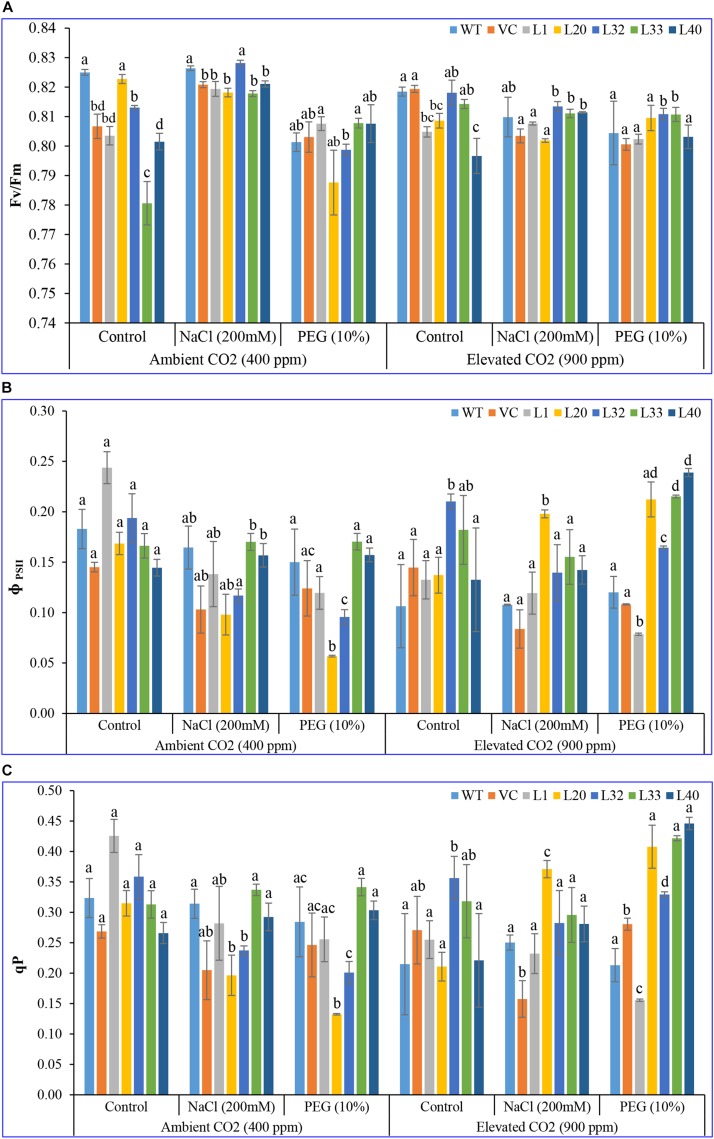
Analysis of the photosynthesis parameters (Fv/Fm, ϕPSII, and photochemical quenching) of transgenic lines. The maximum photochemical efficiency of **(A)** PSII–Fv/Fm, **(B)** the actual photochemical efficiency of PSII–ϕPSII, and **(C)** photochemical quenching of T1 transgenic lines (L1, L20, L32, L33, and L40), wild type (WT), and vector control (VC) were analyzed under salinity (200 mM NaCl) and osmotic [10% polyethylene glycol (PEG)] stress condition grown in ambient and elevated CO_2_ environment. Bars represent means ± SE, and values with different letters are significant at *P* < 0.05.

The pattern of ϕPSII and qP was found to be quite similar in all plants (transgenic lines and control, WT, and VC) under both ambient and elevated CO_2_ levels. At ambient CO_2_ levels, no significant difference was observed between transgenic and control plants, under both control and stress (salinity and drought) treatments. By contrast, at elevated CO_2_ levels, transgenic lines showed a higher ϕPSII and qP than WT and VC plants under control and stress conditions (Figure 4). Consequently, the maximal quantum yield of PSII (Fv/Fm) did not show a significant difference between plants under ambient CO_2_ levels for control and salt stress conditions, although under drought stress conditions, transgenic lines showed a higher Fv/Fm, as compared to WT and VC. However, under elevated CO_2_ stress conditions, transgenic lines exhibited a higher Fv/Fm compared to WT and VC plants (Figure 4). Overall, transgenic lines showed higher values for chlorophyll fluorescence parameters under elevated CO_2_ stress conditions than WT and VC plants. These results suggested that, under stress, the transgenic lines underwent a slight photoinhibition, however, higher photochemical efficiency was still maintained under drought stress at ambient and elevated CO_2_ levels.

The OJIP curve represented a polyphasic fluorescence transient curve, which gave an indication of PSII efficiency under stress conditions (Supplementary Figure S12). The curve for the transgenic lines overlapped with that of WT and VC plants at ambient CO_2_ levels under control and salt treatment, whereas Fm values were higher in transgenic lines than in WT and VC plants. However, under drought treatment, the OJIP curves of the transgenic lines were significantly changed from those of WT and VC plants. Similarly, under elevated CO_2_ levels, no significant changes were noticed. Differences were observed under salt and drought stress treatments, however, the impact on the OJIP curve of transgenic lines was lower than that of WT and VC plants. Compared to control plants, changes observed in the initial fluorescence (Fo) of transgenic lines were comparatively insignificant, however, the maximum fluorescence (Fm) significantly decreased in the WT and VC plants (Supplementary Figure S12). The values of Fm were related to the antenna complex, its structure, function, and energy dissipation. These observations suggested that elevated CO_2_ levels could cause less damage to PSII antenna complexes under salt or drought stress in transgenic lines, compared to corresponding control (WT and VC) plants.

#### Physiological Characteristics

**FIGURE 5:**
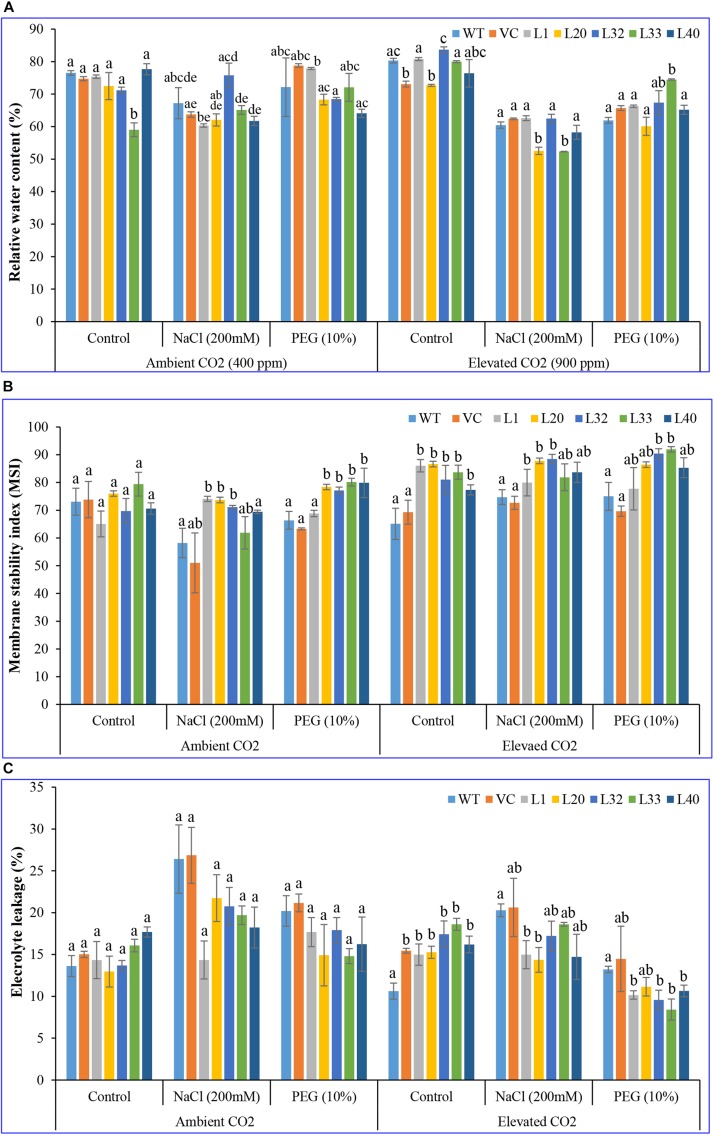
Analysis of the physiological status of transgenic lines. **(A)** Relative water content, **(B)** membrane stability index, and **(C)** electrolyte leakage of wild type (WT), vector control (VC), and T1 transgenic lines (L1, L20, L32, L33, and L40) were measured under salinity (200 mM NaCl) and osmotic [10% polyethylene glycol (PEG)] stress condition grown in ambient and elevated CO_2_ environment. Bars represent means ± SE, and values with different letters are significant at *P* < 0.05.

Change in the RWC of studied plants (transgenic lines, WT, and VC) was insignificant at ambient CO_2_ levels, under control and stress conditions. By contrast, there was a distinct difference for transgenic lines under drought stress at elevated CO_2_ levels (Figure 5A). MSI was considerably higher in transgenic lines (62–93%) than WT and VC plants (51–75%) at both ambient and elevated CO_2_ levels, under control and stress conditions (Figure 5B). In addition, EL was significantly lower in transgenic lines (8.5–18.5) compared to WT and VC plants (13–21) at elevated CO_2_ levels, under control and stressed conditions, whereas EL was higher at ambient CO_2_ compared to that at elevated CO_2_ levels under both control and stress conditions (Figure 5C). At ambient CO_2_ levels, transgenic lines showed higher chlorophyll content under stress compared to WT and VC plants. Elevated CO_2_ levels resulted in significantly higher chlorophyll content in transgenic lines, compared to WT and VC plants, under control and stress treatments (Supplementary Figure S13). By contrast, transgenic lines did not show any significant difference in carotenoid content at ambient and elevated CO_2_ levels under control and stress conditions (Supplementary Figure S13). All these observations suggested that transgenic plants showed an improved physiological status under stress conditions compared to control (WT and VC) plants.

### *SmPPDK* Activity in Transgenic Plants Under Different Stress Conditions and Elevated CO_2_

The maximum PPDK activity was measured in the L40 transgenic line, which was 3.5-fold higher than that of WT plants under control conditions at ambient CO_2_ levels. Similarly, L20 showed 3.8-fold higher activity under salinity stress at ambient CO_2_ levels. PPDK activity was more pronounced under drought stress conditions in ambient CO_2_, being 4.1-fold higher than for WT plants. By contrast, PPDK activity was found to be higher at elevated CO_2_ levels under both control and stress conditions. At elevated CO_2_ levels, the L32 line showed about a 10-fold higher activity under control conditions, while L20 showed a maximum of a 2.2-fold increase under salt stress. Under drought stress, L40 showed a maximum 25-fold higher activity of PPDK compared to the control (WT and VC) plants (Supplementary Figure S14). All these observations indicated that the transgenic lines had higher *Sm*PPDK enzyme activity under abiotic stress in elevated CO_2_ conditions.

*SmPPDK* transcript expression showed a maximum of about 5-fold higher levels in L1 and L33 under NaCl stress conditions, followed by a 3-fold upregulation for L20, whereas a maximum of 6-fold higher expression was noted in L33, followed by a 4.5-fold upregulation for L20 under drought stress, compared to control (unstressed) conditions in an ambient CO_2_ environment. Similarly, transgenic line L32 showed a maximum of 4- and 7.5-fold increase in expression under NaCl and drought stress conditions, respectively, in an elevated CO_2_ environment, followed by an increase shown by transgenic line L1 (Supplementary Figure S14).

### Overexpression of *SmPPDK* Protected Plants From Abiotic Stress-Induced Reactive Oxygen Species Damage

Reactive oxygen species (ROS), such as superoxide radical (O_2_^–^) and hydrogen peroxide (H_2_O_2_), often accumulated when plants were senescing or facing different abiotic stresses. However, some enzymatic antioxidant defense mechanisms could act to protect plants against impairment due to oxidative stress. When plants were subjected to abiotic stress, a large amount of ROS accumulated with the antioxidant system not able to compensate; this situation was often indicated by elevated lipid peroxidation (measured by MDA content) and reduced levels of antioxidant enzymes.

**FIGURE 6:**
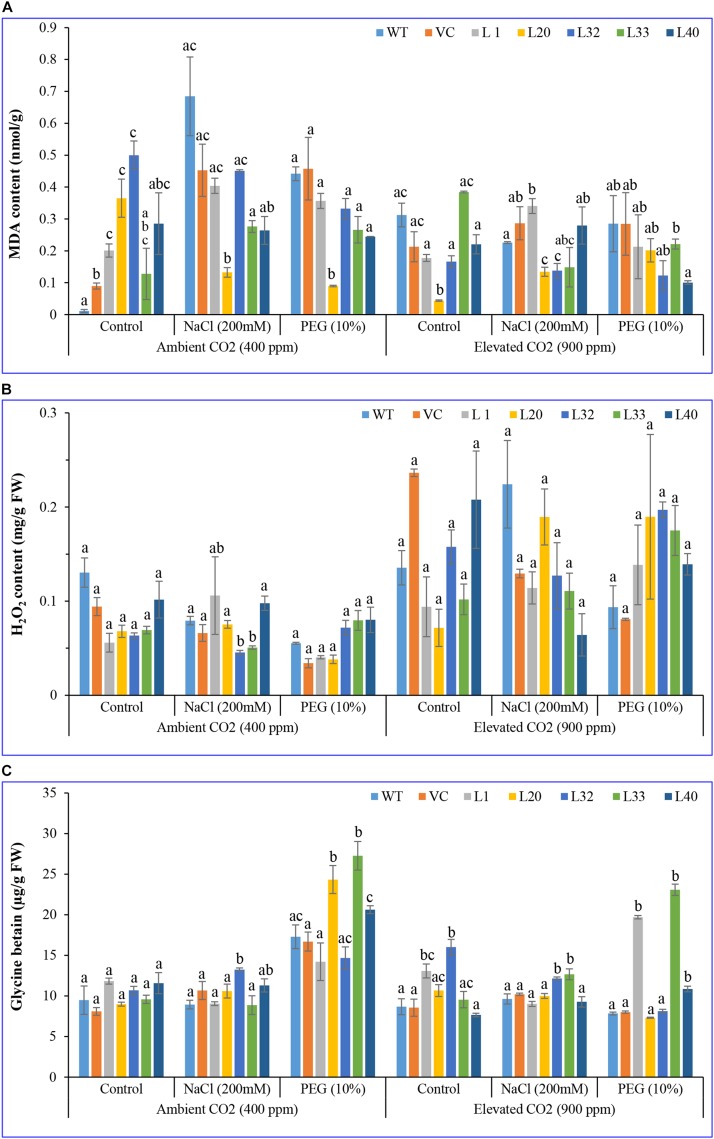
Biochemical analysis of transgenic lines. Estimation of **(A)** lipid peroxidation–malondialdehyde, **(B)** hydrogen peroxide, and **(C)** glycine betaine contents of wild type (WT), vector control (VC), and T1 transgenic lines (L1, L20, L32, L33, and L40) were measured under salinity (200 mM NaCl) and osmotic [10% polyethylene glycol (PEG)] stress condition grown in ambient and elevated CO_2_ environment. Bars represent means ± SE, and values with different letters are significant at *P* < 0.05.

For both ambient and elevated CO_2_ levels, most of the transgenic lines had a lower MDA content, under stress conditions, compared to WT and VC plants (Figure 6A). The accumulation of ROS in transgenic lines was considerably lower than for WT and VC plants under salt and drought stress at both ambient and elevated CO_2_ conditions. Under elevated CO_2_, the MDA content decreased in all samples for control and stress treatments, however, the MDA content of transgenic lines was significantly lower than in WT plants.

For ambient CO_2_ levels, no significant difference was observed in H_2_O_2_ content for transgenic lines under salt stress, compared to that for control plants, however, under drought stress, L32, L33, and L40 lines accumulated a significantly higher amount of H_2_O_2_ compared to WT and VC plants. By contrast, at elevated CO_2_ levels, transgenic lines had a lower level of H_2_O_2_ compared to WT and VC plants, under control and salt stress conditions, whereas under drought stress, all transgenic lines accumulated significantly higher H_2_O_2_ contents (Figure 6B). These results showing a lower level of MDA and a higher level of H_2_O_2_ in transgenic lines indicated that overexpression of the *SmPPDK* gene ameliorated abiotic-stress-induced ROS damage under elevated CO_2_ conditions, with it being more distinct in transgenic lines under drought stress at elevated CO_2_ levels.

GB acted as an osmoprotectant under a stressful environment, in ambient CO_2_ conditions, being significantly increased under stress in all transgenic lines compared to the control plants. However, under salt stress, L32 showed a higher glycine content, whereas under drought stress, L20, L33, and L40 lines had significantly higher glycine betaine as compared to WT and VC plants. Similarly, at elevated CO_2_ conditions, transgenic lines accumulated a higher amount of glycine betaine as compared to WT and VC plants under control and stress conditions (Figure 6C). In addition, increased glycine betaine content was more pronounced in transgenic lines under drought stress at both ambient and elevated CO_2_ conditions.

**FIGURE 7:**
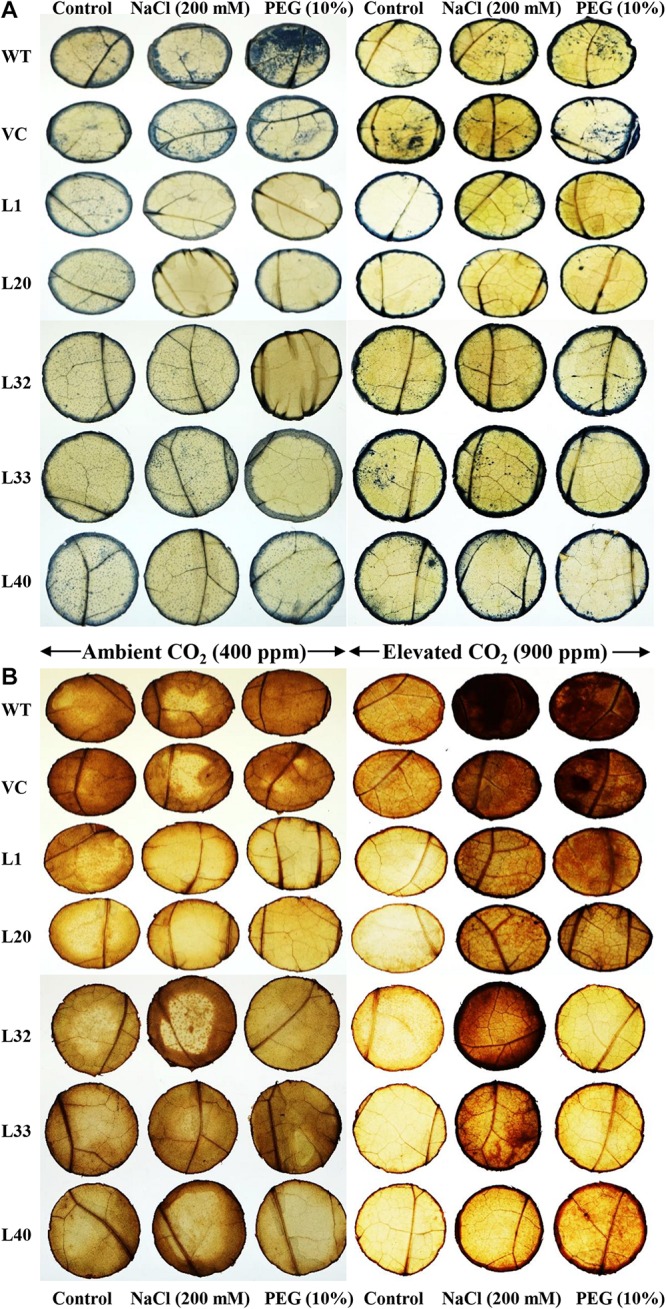
*In vivo* localization of free radicals. Localization of **(A)** superoxide free (O_2_^–^) radicals and **(B)** hydrogen peroxide (H_2_O_2_) in the leaves of wild type (WT), vector control (VC), and T1 transgenic lines (L1, L20, L32, L33, and L40) under salinity (200 mM NaCl) and osmotic [10% polyethylene glycol (PEG)] stress condition grown in ambient and elevated CO_2_ environment.

*In situ*, higher localization of superoxide free radicals (O_2_^–^) and peroxide (H_2_O_2_) was observed in the leaves of control plants (WT and VC) compared to that of transgenic lines, under stress conditions at ambient and elevated CO_2_ levels. Similarly, higher accumulations were also found in elevated CO_2_ conditions compared to ambient CO_2_ levels under different stress treatments (Figure 7).

### Overexpression of the *SmPPDK* Gene Enhanced an Antioxidant Defense System Under Adverse Stress Conditions

**FIGURE 8:**
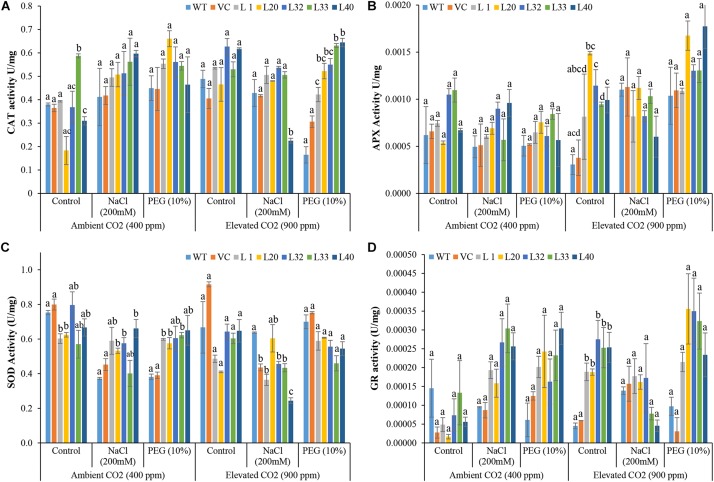
Analysis of reactive oxygen species (ROS) scavenging enzymes activity in transgenic lines. Estimation of **(A)** catalase, **(B)** ascorbate peroxidase, **(C)** superoxide dismutase, and **(D)** glutathione reductase activities in wild type (WT), vector control (VC), and T1 transgenic lines (L1, L20, L32, L33, and L40) were measured under salinity (200 mM NaCl) and osmotic [10% polyethylene glycol (PEG)] stress condition grown in ambient and elevated CO_2_ environment. Bars represent means ± SE, and values with different letters are significant at *P* < 0.05.

High activity of CAT was observed in transgenic plants under stress in both ambient and elevated CO_2_ conditions, as compared to WT and VC plants, although it was significantly more pronounced under drought stress at elevated CO_2_ levels (Figure 8A). Similarly, high APX activity was detected in all stress-treated plants, and the activity increased significantly in the drought-treated transgenic plants under elevated CO_2_ conditions, compared to WT and VC plants (Figure 8B). The activity of SOD was significantly higher in the transgenic lines under salt and drought stress in ambient CO_2_ conditions, as compared to that in WT and VC plants. By contrast, transgenic lines showed significantly lower activity of SOD under control and stress treatments at elevated CO_2_ condition as compared to WT and VC plants (Figure 8C). All transgenic lines showed significantly higher activity of GR under stress conditions in both ambient and elevated CO_2_ environments compared to WT and VC plants (Figure 8D).

Transcript expression analysis of ROS scavenging enzyme-encoding genes from tobacco, e.g., catalase (*NtCAT*), ascorbate peroxidase (*NtAPX*), superoxide dismutase (*NtSOD*), and glutathione reductase (*NtGR*), was performed (Supplementary Figure S15). The transcript level of *NtCAT* decreased in all plants under elevated CO_2_ conditions compared to the control. Transgenic lines L20 and L40 showed about a 3.5-fold upregulation of this transcript under drought stress in ambient CO_2_. The transcript for *NtAPX* increased 2–3-fold under stress at ambient CO_2_ levels. However, at elevated CO_2_ levels, its expression decreased on drought treatment, although it was slightly increased under salt stress (1.5–2-fold). By contrast, in elevated CO_2_ condition, the transgenic line L33 showed a 5- and 3-fold upregulation of the transcript for *NtAPX* under salt and drought stress, respectively Both *NtSOD* and *NtGR* genes were marginally upregulated (1.2–2-fold) in the transgenic under stress conditions in both ambient and elevated CO_2_ environments, except for line L40, which showed a 7- and 3- fold upregulation of the *NtGR* gene under salt and drought stress, respectively, in ambient CO_2_ conditions.

### Overexpression of the *SmPPDK* Gene Mitigated the Effect of Abiotic Stress Under Elevated CO_2_ by Lowering the Photorespiration Rate in Transgenic Lines

Photorespiration was considered the second most imperative metabolism in plants. However, it increased under stress conditions, such as drought and salinity, leading to H_2_O_2_ production in plant cells. The Gly/Ser ratio, one of the primary indicators of photorespiration, was significantly higher in selected transgenic plants and WT plants under ambient CO_2_ conditions compared to plants grown under stress (Supplementary Figure S16A). By contrast, the ratio was lower in transgenic lines grown under an elevated CO_2_ environment compared to WT plants. Photorespiration-associated genes from tobacco, *NtHPR* and *NtGO*, were differentially expressed under different stress conditions. In an ambient CO_2_ environment, the *NtHPR* gene was downregulated in transgenic lines under salt and drought stress conditions, compared to WT and VC plants (Supplementary Figure S16B). Similarly, at elevated CO_2_ levels, expression of *NtHPR* gene was higher in control plants compared to transgenic lines under stress conditions. By contrast, the *NtGO* gene was considerably upregulated in an elevated CO_2_ environment compared to ambient CO_2_ conditions (Supplementary Figure S16C). In ambient CO_2_ conditions, *NtGO* gene was downregulated in transgenic lines under salt (except L1) and drought (except L1 and L20) stress, whereas in elevated CO_2_ conditions, the *NtGO* gene was upregulated in all stress conditions except for L40 under NaCl.

### *SmPPDK* Transgenic Lines Accumulated Sugars, Polyphenols, and Free Amino Acids Under Abiotic Stress Conditions

In ambient and elevated CO_2_ conditions, total soluble sugar and reducing sugar were found to be significantly higher in transgenic lines under control conditions compared to WT and VC plants, however, this effect was more pronounced at elevated CO_2_ levels (Supplementary Figure S17). However, at ambient CO_2_ levels under salt stress, no significant difference in total sugar and reducing sugar contents were noticed among transgenic lines and WT and VC plants. By contrast, in an elevated CO_2_ state, transgenic lines accumulated a significantly higher amount of total sugar and reducing sugar under salt and drought stress, compared with WT and VC plants. The starch content increased considerably in L20 and L32 under control conditions, whereas it was reduced under salt and drought stress at ambient CO_2_ levels (Supplementary Figure S17). Under drought stress, L1 and L40 accumulated about a 12- and 5-fold higher starch content, respectively, compared to WT plants. By contrast, under elevated CO_2_ conditions, all transgenic lines accumulated high starch contents (Supplementary Figure S17).

Free amino acid (FAA) content showed no distinction among transgenic lines and WT and VC plants under control and NaCl stress conditions at ambient CO_2_ levels. However, it increased considerably under drought stress in transgenic lines compared to WT and VC plants. By contrast, at elevated CO_2_ levels, FAA content was significantly increased in transgenic lines under control, NaCl, and drought stress conditions compared with WT and VC plants (Supplementary Figure S18). Transgenic lines showed high polyphenol contents compared to control plants, however, they did not change significantly under stress conditions (Supplementary Figure S18). Overall, the results suggested that the transgenic lines accumulated a higher amount of sugars, FAA, and polyphenols to protect the host plant under stress conditions.

### Analysis of Metabolites

In total, 43 and 33 metabolites were detected in plants grown in ambient and elevated CO_2_ environments, respectively (Supplementary Tables S2, S3). Out of these, 25 metabolites were common to both conditions, 18 were unique to ambient conditions, while 8 metabolites were accumulated exclusively under elevated CO_2_ levels. Metabolites were differentially accumulated in plants (WT, and transgenic lines L20 and L40) under different stress conditions (salt and drought) in both ambient and elevated CO_2_ environments.

The metabolites commonly identified in WT and transgenic plants under ambient CO_2_ and control and stress conditions were 11 amino acids, 10 sugars, 6 organic acids, 3 sugar acids, 4 fatty acids, and 5 miscellaneous metabolites (Supplementary Table S2). The metabolites commonly identified in WT and transgenic plants under elevated CO_2_ (900 ppm) and control and stress conditions were 19 amino acids, 7 sugars, 1 sugar acid and 1 sugar alcohol, 4 organic acids, 1 fatty acid, 1 amine, and 2 miscellaneous metabolites (Supplementary Table S3). Out of 19 amino acids, only 6 amino acids, adenine, L-leucine, L-lysine, L-tryptophan, L-valine, and β-alanine, were differentially expressed in WT and transgenic lines under elevated CO_2_ levels in control and stress conditions, however, not at ambient CO_2_ levels in control and stress conditions. The concentration of asparagine was higher in transgenic lines under elevated CO_2_ and control and stress conditions, as compared to WT plants, and was also increased under ambient CO_2_ stress conditions. L-Proline was one of the most important indicators of drought stress, acting as an osmoprotectant to mitigate damage in plants under stress conditions. However, level of L-proline in *SmPPDK* transgenic lines were decreased under salt and drought stress in both ambient and elevated CO_2_ as compared to WT and control conditions. By contrast, accumulation of L-aspartic acid was increased under salt stress but downregulated under drought stress in transgenic lines at ambient CO_2_ levels; whereas, in elevated CO_2_ conditions, the opposite situation occurred, with L-aspartic acid being upregulated in transgenic lines under drought stress, compared to WT plants. Some important photorespiration metabolites, including glycine, serine, L-glutamic acid, and L-glutamine, were downregulated in transgenic lines under stress conditions at ambient CO_2_ levels, relative to WT plants, however, their expression was not significantly altered under elevated CO_2_ and control and stress conditions. Important sugar metabolites, such as fructose, galactose, and glucose, were differentially expressed in WT and transgenic lines. There was a considerably higher accumulation of sugar in transgenic lines in control conditions but downregulation under stress conditions at both ambient and elevated CO_2_ levels, as compared to WT plants. In some important Kreb’s cycle intermediates, such as citric acid, 2-ketoglutaric acid, and malic acid, accumulation showed considerably higher accumulation in transgenic lines under ambient CO_2_ but were downregulated at elevated CO_2_ levels, compared to WT plants.

### Overexpression of *SmPPDK* Improved Plant Performance Without Affecting Yield

**FIGURE 9:**
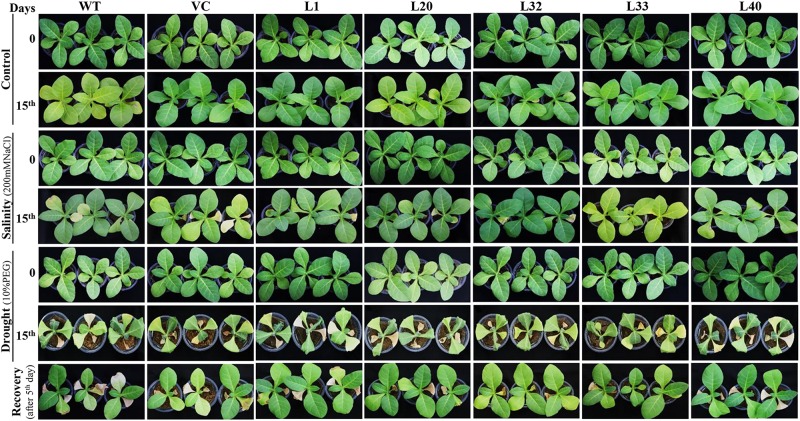
Comparative plant growth studies under salt and osmotic stress. Morphological studies of wild type (WT), vector control (VC), and T1 transgenic lines (L1, L20, L32, L33, and L40) under salinity (200 mM NaCl) and osmotic [10% polyethylene glycol (PEG)] stress condition.

One-week-old seedlings (transgenic and control plants) were transferred to a soil-filled pot and allowed to grow under normal growth conditions for 30 days. After being subjected to salt and drought stress treatments for a further 15 days, plant growth was documented and compared to control plants (Figure 9) and those treated under control conditions. Transgenic plants were only marginally affected or actually grew better under stress conditions compared to WT and VC plants. In addition, drought-treated plants recovered after the fifth day of restarting irrigation, with transgenic lines recovering faster than WT and VC plants. Transgenic and control (WT and VC) plants were also grown under normal greenhouse conditions and allowed to complete their life cycle (Supplementary Figure S19). No differences were found in vegetative performance between plants (Supplementary Table S4), however, comparatively delayed flowering was observed in WT and VC (control) plants (Supplementary Figure S19), with flowering time of transgenic lines being considerably shorter. In addition, L1 and L40 gave a significantly higher number of pods per plant than WT and VC plants. Even so, all transgenic lines displayed higher plant dry weight compared to that of WT and VC plants. Overall, these observations confirmed that integration of the C_4_-specific *PPDK* gene into C_3_ tobacco plants improved plant morphology, growth characteristics, and plant performance, without imposing any adverse effects.

## Discussion

An approach for increasing crop yield and photosynthetic efficiency in current climate conditions involves the ectopic expression of C_4_ genes in existing C_3_ crops. C_4_ plants have advantages under different climatic and environmental conditions, such as high salinity, drought, higher temperatures, and nitrogen or CO_2_ limitation, with these plants having a higher affinity for CO_2_. The overexpression of C_4_ PPDK, on its own or in combination with other C_4_ enzymes, has been performed in a number of C_3_ crops, with some of the transgenic plants showing greater biomass, while others exhibited alterations in photosynthetic efficiency (Yadav and Mishra, 2020).

In this current study, a C_4_-specific *PPDK* gene was cloned from single-celled *S. monoica*, and integrated into a C_3_ modal plant tobacco genome under the control of the CaMV35S constitutive promoter, using *Agrobacterium*-mediated genetic transformation (Figure 1 and Supplementary Figure S2). Previously, introgression of the maize *PPDK* gene increased photosynthesis efficiency and yield of transgenic lines by enhancing PPDK activity in leaves (Fukayama et al., 2001). The *SmPPDK* gene comprised of seven exons and six introns in the transgenic lines (Supplementary Figure S1), and overexpression *SmPPDK* cDNA resulted in increased plant biomass, vegetative growth, and sugar content (including total sugar, reducing sugar, and starch) under different stress conditions (Supplementary Figures S5–S9, S17). The *PPDK* gene played an important role in sugar metabolism, as transgenic plants grew efficiently under sugar deficiency (caused by stress) compared to WT and VC plants.

Photosynthesis is a primary metabolic process in the production of biomass, however, it is sensitive to different abiotic stresses. We observed a decrease in the rate of photosynthesis at elevated CO_2_ levels and under abiotic stresses (salt and drought), however, this decrease was lower in transgenic lines compared to WT and VC plants (Figure 2). Commonly, photosynthesis increases under elevated CO_2_ condition due to greater availability of carbon triggering higher Rubisco activity. Higher photosynthesis increases the amount of reserve carbohydrates in leaves, and this downregulates the expression of photosynthetic gene, thereby decreasing the photosynthesis (Cheng et al., 1998). It is a type of feedback inhibition in which photosynthesis decreases under long-term elevated CO_2_ exposure (Thompson et al., 2017).

Interestingly, the reduction in photosynthesis rate was found to be linked with transpiration under elevated CO_2_ conditions. In transgenic plants, increased total chlorophyll content was estimated due to high activity of C_4_ *Sm*PPDK, which improved photosynthetic efficiency (Supplementary Figures S13, S14). The integration of the transgene maintained the chlorophyll fluorescence efficiency without affecting the electron transport chain, under different abiotic stress conditions (Figures 2–4 and Supplementary Figure S12). In addition, PSII utilized the most energy by chlorophyll for photosynthesis, and it was considered a useful indicator to measure the effects of abiotic stress on plants (Maxwell and Johnson, 2000). Chlorophyll “a” fluorescence efficiency was also correlated with the content of total chlorophyll in leaves, and interestingly, transgenic lines showed increased chlorophyll content with higher chlorophyll “a” fluorescence efficiency. Fv/Fm ratio is a widely used parameter for measuring the status of PSII under stress conditions. *SmPPDK* transgenic plants did not show a significant change in Fv/Fm ratio, compared to WT and VC plants, under stress conditions in ambient CO_2_ condition, but performed better under stress at elevated CO_2_ levels (Zhao et al., 2007). Overall, the pattern of chlorophyll fluorescence suggested that a high Fv/Fm ratio under salt and drought stress at both ambient and elevated CO_2_ levels indicated higher photosynthesis and maximum quantum efficiency of PSII centers in transgenic plants. This resulted in an increase in biomass, CO_2_ assimilation, and yield in transgenic lines compared to WT and VC plants under adverse climate conditions (Supplementary Table S4). In a previous study, similar results were also obtained when the maize C_4_ *PPDK* gene was overexpressed in rice, with transgenic lines having larger panicles and greater yield compared to WT plants (Wang et al., 2004). There are several reports confirming that ectopic expression of a C_4_-specific *PPDK* gene in C_3_ plants has synergistic effects on photosynthesis (Ding et al., 2013; Zhang et al., 2014). Photosynthesis efficiency was also found to be positively correlated with stomatal conductance and internal CO_2_ concentrations (Ku et al., 2000).

Chlorophyll–fluorescence transient curve (OJIP) have been extensively used to study PSII activity (Yusuf et al., 2010), and those in the current study (Supplementary Figure S12) suggested that salt and drought stress were major constraints on PSII activity in the host plant. In addition, the expression of the exogenous *SmPPDK* gene alleviated damage to the PSII system and thus maintained photosynthetic electron transportation under adverse conditions. As such, *SmPPDK* transgenic tobacco plants maintained a comparatively high photochemical efficiency. Transgenic lines also maintained relatively higher water content under drought stress in elevated CO_2_ conditions compared to WT and VC plants (Figure 5). Improved RWC could also be correlated with an increase in stomatal conductance, and this was directly regulated by different C_4_ enzymes of organic acid metabolism, which modulate stomatal conductance by influencing guard cells (Lawson et al., 2018).

Transgenic lines had higher *Sm*PPDK activity compared to WT and VC plants, and the increment was greater under elevated CO_2_ conditions (Supplementary Figure S14). We previously reported that the activity of PPDK enzyme increased under elevated CO_2_ conditions in *Suaeda* spp. (Yadav et al., 2018). PPDK, involved in C_4_ photosynthesis, was more resistant to abiotic stress and elevated CO_2_ levels, ameliorating the adverse effects of induced stress. Previously, about a 5-fold increase in PPDK activity was estimated for transgenic lines compared to untransformed controls (Jiao et al., 2002). Enzymes, which are involved in C_4_ carbon assimilation, are known to accumulate in leaves under different abiotic stress conditions (Fißlthaler et al., 1995). These enzymes play a crucial role in plant responses to drought by minimizing the production of ROS and stabilizing membrane lipid peroxidation (Jiao et al., 2002; Gu et al., 2013). Similar results were observed in the current study, with a lower accumulation of MDA (i.e., lower lipid peroxidation) and H_2_O_2_ in transgenic lines under stress and elevated CO_2_ condition compared to WT and VC plants, which accounted for the improved tolerance of plants overexpressing the C_4_-specific *SmPPDK* gene (Figures 6, 7). Furthermore, lower production and localization of superoxide and peroxide free radicals were observed in transgenic lines compared to WT and VC plants. This suggested the *SmPPDK* gene mitigated oxidative stress by ROS scavenging activity.

Elevated CO_2_ reduces H_2_O_2_ accumulation by lowering the photorespiration rate, mitigating oxidative stress, and also by inducing protective mechanisms for the photosynthetic apparatus. However, improvements in photosynthesis efficiency due to enrichment of CO_2_ might also enhance the activity of antioxidant enzymes, leading to lower production of ROS (Zinta et al., 2014). The Gly/Ser ratio is an important parameter, which provides evidence that photorespiration is suppressed under elevated CO_2_ conditions (Kebeish et al., 2007). Transgenic lines showed reduced Gly/Ser ratios under stress conditions, with this ratio being considerably decreased under elevated CO_2_ stress conditions (Supplementary Figure S16). A decrease in Gly/Ser ratio was similarly observed in *A. thaliana* under elevated CO_2_ conditions, which was correlated with a lowering of photorespiration (Kebeish et al., 2007).

It has been well established that plants accumulate high levels of ROS under different stresses, and commonly, antioxidant systems are not adequate, a situation characteristically indicated by lipid peroxidation and low activity of antioxidant enzymes (Gill and Tuteja, 2010; Alexander et al., 2019). Elevated CO_2_ increased the level of antioxidants, including polyphenols, ascorbate, alkaloids, and the activity of some antioxidant enzymes (SOD, CAT, GR, and APX), which improve the antioxidant defense mechanisms and reduce ROS generation (Zinta et al., 2014). In the current study, *SmPPDK* transgenic lines showed high antioxidant activities under stress conditions, facilitating efficient survival under adverse conditions (Figure 8). Osmoprotectants such as glycine betaine, different sugars, FAAs, and polyphenols are considered essential biochemical stress markers for determining the response of plants to osmotic stress (Ksouri et al., 2007). The overexpressing *SmPPDK* transgenic lines accumulated high glycine betaine, total sugar, reducing sugars, polyphenols, and FAAs, with these accumulations increasing under elevated CO_2_ conditions (Figure 6 and Supplementary Figures S17, S18). In general, accumulation of polyphenols and different sugars help protect plants against ROS (Ksouri et al., 2007). These compounds accumulate to conserve the osmotic equilibrium across the membranes under osmotic stress conditions. Glycine betaine is mainly found in chloroplasts and plays an important role in the protection of thylakoid membranes, thus conserving photosynthetic activity. In addition, at higher salinity, it protects PSII, membranes, and Rubisco from osmotic stress (Jagendorf and Takabe, 2001). Under salt and drought stress conditions, glycine betaine and the antioxidant defense system have been shown to have a protective effect in wheat and suspension cultures of tobacco BY2 cells (Raza et al., 2007).

Wang et al. (2009) validated the two main ROS detoxifying enzymes, CAT and APX. In the current study, the activities of these enzymes were elevated in *SmPPDK* transgenic lines exposed to salinity, as compared to control plants. Furthermore, *SmPPDK* transgenic lines could detoxify ROS more efficiently; therefore, they showed tolerance under stress. Transcript expression analysis (Supplementary Figure S15) showed that in ambient CO_2_, *NtCAT*, and *NtAPX* were upregulated in transgenic lines under both NaCl and drought stress. By contrast, elevated CO_2_ resulted in downregulation of *NtCAT* expression in transgenic lines under stress conditions, as compared to ambient CO_2._ Likewise, *NtAPX* and *NtSOD* were also induced in all transgenic plants under elevated CO_2_ as compared to control plants. At elevated CO_2_ levels, *NtGR* was also upregulated significantly under both salt and drought stresses, relative to WT plants under the control conditions.

The different types and quantities of metabolites, as final products of cell regulation mechanisms, can be assayed to measure the direct response of biological systems to any environmental or genetic change (Patel et al., 2016). They are involved in various functions such as osmotic alterations, antioxidant protection, and signal transduction (Fiehn, 2002). Secondary metabolites play a key role in defense under stress condition (Tanna and Mishra, 2018). Plants produce different metabolites in response to abiotic stress and accumulate organic acids, sugars, polyamines, amino acids, and lipids (Guy et al., 2008). In this current present study, we examined the synthesis of metabolites as a prospective mechanism for improving photosynthesis and for stress tolerance in tobacco transformed with the *SmPPDK* gene. Accumulation of sugar alcohols mitigated the damage caused by stress and provided tolerance. *Salicornia brachia*, an extreme halophyte, accumulates a number of metabolites as a protective mechanism under salt and drought stress conditions (Mishra et al., 2015).

Under elevated CO_2_ conditions, the CO_2_/O_2_ ratio increased at sites of photoreduction in leaves due to a higher CO_2_ concentration gradient. Consequentially, the rate of photorespiration and formation of ROS decreased due to an increase in NADPH consumption. Pn and carbon supply is also increased, which leads to an enhanced WUE for photosynthesis (Morgan et al., 2011). All these effects minimize the oxidative stress, and further energy could be used for energy-dependent stress mitigation mechanisms, such as the production of osmolytes and antioxidants (Drake et al., 1997).

Overexpression of individual genes in different species is currently one of the more common practices in molecular biolog, however, it is a complicated process and is controlled by many different factors, such as transcriptional and posttranscriptional modifications, integration site, and transgene copy number (Ma et al., 2011). Although these factors will cause various phenotypic and biochemical changes, their exact mechanisms are still unknown. This was the first report of a C_4_-specific *PPDK* gene from single-celled *S. monoica* improving photosynthesis performance under salt and drought stress conditions at ambient and elevated CO_2_ levels. In this study, we observed that transgenic lines showed earlier flowering than control plants due to increases in carbon assimilation efficiency by decreasing the rate of photorespiration. Furthermore, transgenic plants were shown to produce a higher number of leaves per plant by increasing soluble sugar. Overall, *SmPPDK* transgenic lines also performed better in control field conditions (Supplementary Table S5) completing their life cycle earlier and with higher biomass than WT and VC plants. We hypothesized that reduction in photorespiration and ROS production in transgenic lines by improved antioxidant defense systems, enhanced photosynthesis performance of C_3_ transgenic tobacco lines under salt and drought stress conditions, at ambient and elevated CO_2_ levels, however, further molecular studies will be required to support this hypothesis.

## Data Availability Statement

The datasets generated for this study can be found in the MK704410.

## Author Contributions

AM: conceptualization and designing. SY: conduct of the experiments. SY and MR: data acquisition. SY and AM: analysis and interpretation, and drafting of the manuscript.

## Conflict of Interest

The authors declare that the research was conducted in the absence of any commercial or financial relationships that could be construed as a potential conflict of interest.
