# Supplementary material for: The Pyruvate-Phosphate Dikinase (C4-SmPPDK) Gene From Suaeda monoica Enhances Photosynthesis, Carbon Assimilation, and Abiotic Stress Tolerance in a C3 Plant Under Elevated CO2 Conditions
Source: Front Plant Sci. 2020 Apr 21;11:345. doi: 10.3389/fpls.2020.00345 (PMC7186359; doi:10.3389/fpls.2020.00345)

Table S1: List of primers and PCR conditions used in the study

Events/ purpose	Gene/Primer	Primer Sequences (5'→3')	PCR Conditions
Transcript profiling of *PPDK* Gene	PPTr:F<br>PPTr:R	GGTAAGGAATGAAACTAGCCCAGAGG<br>GATCTCAGAGCACCCTGAAACACAAC	94°C- 2 min; 94°C- 10 s; 58°C- 15 s; 72°C- 1 min; (40 cycle); 72°C- 7 min
Isolation of full length PPDK cDNA	PPDK:F<br>PPDK:R	***GGTACC***ATGGCATCTGCTTTTAAAGGAATG<br>***TCTAGA***TCAAACTGCAACTTGAGTCTGC	94°C- 5 min; 94°C- 1 min; 60°C- 1 min; 72°C- 3 min; (35 cycle); 72°C- 10 min
3' RACE of *PPDK* gene (reverse primer)	3'GSP-1<br>3'GSP-2<br>3'GSP-3<br>3'GSP-4	TTTGCTTCAGATGATAGGAT<br>ATGGATGGGCTTCCTGTGA<br>ACTGAAATGCAAGCACGG<br>TCACAGGAAGCCCATCCAT	94°C- 5 min; 94°C- 30 s; 58°C- 45 s; 72°C- 1 min; (35 cycle); 72°C- 5 min
5' RACE of *PPDK* gene (forward primer)	5'GSP-1<br>5'GSP-2<br>5'GSP-3<br>5'GSP-4<br>5'GSP-5<br>5'GSP-6<br>5'GSP-7<br>5'GSP-8<br>5'GSP-9<br>5'GSP-10<br>5'GSP-11	TTTGATAGCTTCAGGCATG<br>ATCTTCTCCCTGAGCATTAATCT<br>AACACCTGTTCCTGAAGTATT<br>CCCAGGAATCAAACACCGC<br>GCTTTTCTAACTTCTCCTCG<br>AGTCATAGGCAAAGCGTTCTC<br>GACAGTGTCCATCATCCCTG<br>GCGAGATTTGCTCCTTTACC<br>GAGGTCACAAGCCGTCATG<br>GGTAAAGGAAGGAGTGAAGG<br>ATGGCWTCWGCTTWTAAAGG	94°C- 5 min; 94°C- 1 min; 60°C- 1 min; 72°C- 3 min; (35 cycle); 72°C- 10 min
Transcript profiling of host (tobacco) genes: antioxidant enzyme encoding genes, and genes that involve in photorespiration	NtActin:F<br>NtActin:R	CGTTTGGATCTTGCTGGTCGT<br>CAGCAATGCCAGGGAACATAG	94°C- 3 min; 94°C- 10 s; 58°C- 30 s; 72°C- 1 min; (40 cycle); 72°C- 10 min
	NtSOD:F<br>NtSOD:R	AGCTACATGACGCCATTTCC<br>CCCTGTAAAGCAGCACCTTC	
	NtCAT:F<br>NtCAT:R	AGGTACCGCTCATTCACACC<br>AAGCAAGCTTTTGACCCAGA	
	NtAPX:F<br>NtAPX:R	CAAATGTAAGAGGAAACTCAGAGGA<br>CAGCCTTGAGCCTCATGGTACCG	
	NtGR:F<br>NtGR:R	ATAGATTCTGATGCTGCCCCTTG<br>CGACTCCTCAGTATGGAACTCAA	
	NtHPR:F<br>NtHPR:R	CAATTCCTGAAAGCCAGTGGTG<br>CTCATCCTCAAAGACATCAAGGC	
	NtGO:F<br>NtGO:R	GCAGGTCTACAAGGACAGGAAT<br>ACAAATCTGCAAGGTGGTGGA	
Genome organization study (exon and intron)	PPDK-F<br>PPDK-R	CGAGGAGAAGTTAGAAAAGC	94°C- 5 min; 94°C- 30 s; 60°C- 45 s; 72°C- 1 min; (35 cycle); 72°C- 10 min
Molecular identification of transgenic lines by *uid A* and *hptll* gens	gus:F<br>gus:R	GATCGCGAAAACTGTGGAAT<br>TGAGCGTCGCAGAACATTAC	95°C- 5 min; 95°C- 45 s; 55°C- 45 s; 72°C- 1min; (35 cycles); 72°C- 10 min
	hpt:F<br>hpt:R	TTCTTTGCCCTCGGACGAGTG<br>ACAGCGTCTCCGACCTGATG	
Copy number analysis of transgene in transgenic lines by Real- time PCR	GQ:F<br>GQ:R	GTGAAGGGCCAACAGTTCC<br>GGTAATGCGAGGTACGGTA	94°C- 5 min, 95°C- 45 s, 55°C- 45 s, (35 cycles); 72°C- 1 min; 72 °C- 5 min
	NRA:F<br>NRA:R	AATGCTGGCACTGATTGCAC<br>TCCTCTGCGCTGGAACAAG	

Note: Enzyme cutting sites— *GGTACC*– BamHI, and *TCTAGA*– XbaI

Exon 1

ATGGCATTATCTTTCAAAGGAATGCTGGTCAGAACTCCTGATATATGCACACAGACACTTCTGAAAGGGAAGCACTTACATCAGATTGGTTTTTTGAAGGACAAATATGAACTTGATTGTAGCCAATTCGATATTCGCCATGT

TCAATTCCAGAACAGGAAACACCCACATCGACTTGGAAGACAATGCAAGTCACAAGTTGACATGGCTCTGGTCTCAGAGCCAGTTCCAACCACCAAAAAGGTACACAATAAATAGTTGGCTGTGCTTTAAATTAGGCATATGA

ACGTGAACATTTGTCTCAAATGGAAAATATGTCTACCCATATTAATTATTTTCATGCTCATTTGTTTCGAGGCGTTACATTTTTCTTTTTGCATAAACCTAAGTGACTGATCTGTACTGCTTATCAATTGATATTACATCTTT

Exon 2

TCTTGAGCAGCGAGTATTTACTTTTGGTAAAGGAAGAAGTGATGGCGACAAGAGCATGAAGTCCTTGGTAGTAATTTGGCTTTAGATATGCGTATTTGACAAGTGAACTGAACGTTTTCATGGCCTGATATCACTCCTTGATA

TACTTAATCTGGTTATATAATGTTCTTAGTCCAAGTGGTAAAATAAGCTTGAGCTTAATACTCTACCCTATACCATCAAAGTAACATGGTTTTTCAACCAATAATGCTAGCAAAACTCACTAATTAACAACAACAACAACAAC

AATAAAGCCTTAGTCAAAATAGTTGGGGGTCGGTTACACATGAAATAATCATGATAATTGCAAAATTCATCTGCGTAAAACGTCTTCCTCCATTTTTCCTTGTTTAACACCAAGTCCCAACATTCTCATATCGCCAACAATCA

CCCTCATCCAAGTTTGCTTTGGTCTTACTCTACCTCTCACCTGTCCATCTTTCGGCCACTCTTCAAGCCTCTTACCGGTGTGTTACTCGTGATCATTGGTCCAATGCTTGGGGTGAAATCAATTCCCAGAGTGTGACAAACGG

TGTGTCTAATATGACCAAACCATCTTAGATGTTTTCCCTCATCTTATAGTCAATAGATGGAGTCGTCGCCTTCTCAAAACTAACTAATTAATATGTTTATAAAAAGTTTGTCATAATTTGTAGTGCTAAGGTATCTCTTTTTC

TGAGTTATTGTGCAGTTGGGAGGTAAAGGAGCAAATCTCGCAGAAATGGCAAGTATAGGCTTATCCGTTCCTCCTGGGTTGACAATTTCTACAGAAGCATGCCAAGAATATCAGGATGTTGGGAAAAAGCTTCCCGAAAGTTT

Exon 3

GTGGGAAGAAATACTTGAGGGTTTGAGGGTGATAGAGAATGACATGGGAGCGTACCTTGGAGACTCCTCAAAACCTCTTCTACTTTCAGTTCGTTCTGGTGCTGCAGGTAAGATGCTAAAATTTCGTCACATTAGTGACATAT

CTGTCTGATTCGAATTTGATATGTAATTTCTTTCAATTCAAATGTGTTTTTCTTACAATTGTCATGCTTTGTTCTTACTTCCTCAACAAATTTTGGCATCATGCATCTACAGATTTCCATGCCAGGGATGATGGACACTGTCT

Exon 4

TAAATCTTGGACTTAATGATGAAGTGGTAGTAGGGCTGGCAGCAAAAAGTGGAGAACGCTTTGCCTATGACTCATTTAGGCGTTTCTTGGACATGTTTGGTGGTGTAGTAAGTTTCTACCTCTGTTTGTGTCATCAATATTTC

TCTTAGTTTGTTGTTTCTTAAGCCTTTTTTTCTCTCGTTGAATGTTGACAACTTCAAACCTCAGTATAATGCTTCTTTTCAATCAAAAGCCTGAGACTGTTCAGAGAAATGCTTTTCTCAAGAATTATAAGCCTAAGGAATTA

TCATTCCGTACTTTGTTCTGATTTTTTTTCTTTCATTTCAAATCTTTAGAATGATTAAGATTATCAAAGCTTGTATGTGGCCTTTATACAGGACTTGAAGAATTACATGAATAGATGCTAGCTAAAATACACCTTTTGTTCTT

Exon 5

GAAGGTCATGGGGATTCCTCACTCAGCATTCGAGGAGAAGTTAGAAAAGCTGAAACAAACCAAAGGAGTTAGACTTGATACTGAACTAACTTCTTCCGATCTGAAAGAACTTGCAGAGCAATACAAAAATGTGTATATTGAAA

CAAAAGGAGAGGTTTTTCCAGCTGGTATGAAACTAACTTTCCTGTTTTTCTCTTAACTCCTTCCTCCTAGAATGGACTAAATCTTAGGGCACAAACTCACTAATTTCCAGATCCAAATAAATTTTGGAGTTTCAGTGCTAAAG

CTTTCTTTTTTCTGTAATCTTAATCAATCTCTAGATCCAAAGAAACAGCTTCAGTTAGCAATTCAAGCGGTGTTTGATTCCTGGGACAGTCCAAGAGCTGTTAAATATAGAAGCATCAACCAGATCTCTGGGTTGAAAGGAAC

Exon 6

TGCAGTCAACATCCAGTCTATGGTATTTGGTAATATGGGCAATACTTCAGGAACAGGTGTTCTTTTTACTCGAAATCCAAGCACTGGTGAAAAGAAGCTCTATGGAGAGTTTTTAATTAATGCTCAGGTATACTATAATCTCG

TAATTATTAACTTATAGAGAACATTGCAGTTGCTCTGTTTTCATATATTGATTGAAACTTAATAAACAGGGAGAAGATGTGGTCGCTGGCATTAGAACACCAGAAGACTTGGATGCCATGAAGAACTGCATGCCTGAAGCTTA

Exon 7

CAAAGAATTAGTTCAGAACTGTGAAATTTTAGAACAACATTACAAGGATATGATGGATATTGAATTCACCGTTCAAGAAAATAGGCTGTGGATGTTGCAATGCCGTTCTGGTAAGCGTACTGGAGCAGGTGCGGTGAAGATTGC
TGTAGATTTGGTGAATGAAGGAATTGTAAACTCCCGTGCTGCTGTTAAAATGGTGGAACCACAACATCTTGACCAACTTCTTCATCCACAGTTCGAAGACCCATCTGCATATAAAGATAGTGTGATTGCCCGTGGACTGCCAGC
ATCTCCTGGAGCTGCTGTGGGGCAGATTGTATTCAGTGCTGAGGATGCCGAAGCCTGGCATGCACAAGGGAAGAGTGCTATTCTGGTAAGGAATGAAACTAGCCCAGAGGATGTTGGGGGTATGCATGCAGCTGCTGGAATCTT
GACTGCTAGGGGAGGAATGACATCTCATGCTGCGGTCGTGGCTCGTGGCTGGGGAAAGTGTTGTGTTTCAGGGTGCTCTGAGATCCAAGTAAATGAAACCAACAAGGTTCTTGTGGCTGGAGATAAAGTCATAGCTGAAGGAGA
ATGGCTTTCTCTAAATGGGTCTACGGGTGAAGTGATCTTAGGGAAACAGCCACTTGCACCGCCTGCGCTCAGTGGAGATTTAGAAACCGTGATGTCTTGGGCTGATGCCGTACGACGTATTAAGGTGATGGCCAATGCAGATAC
ACCAGAGGATGCATTGACAGCAAGAAACAATGGTGCCCAAGGGATTGGCCTTTGCCGGACTGAGCACATGTTTTTTGCTTCAGATGATAGGATTAAGACAGTGAGGAAAATGATAATGGCAGTTACACCTGAGCAGAGGAAAGC
AGCTTTGGATCAGTTATTACCTTACCAGAGATCTGATTTTGAAGGGATTTTTCGAGCCATGGATGGGCTTCCTGTGACAATAAGATTGCTAGACCCTCCATTGCATGAGTTTCTTCCAGAAGGTGATTTGGAACAAATTGTCAA
TGAGTTGGCCTCCGAAACTGGAGTGAACAAAGATGAAGTCTTTTCCAGGATAGAAAAGCTATCTGAAGTTAATCCCATGTTGGGCTTCAGGGGTTGCAGGTTAGGGATATCATATCCCGAACTAACTGAAATGCAAGCACGTGC
AGTATTTCAAGCTGCAGTTTCAATGAGCAACCAGGGATTTACGGTTCTACCTGAAATCATGGTTCCTCTCGTTGGAACACCTCAGGAGTTAGGGAATCAAGTGAGTTTGATACGAAATGTTGCAACAAATGTATTCTCAGAGAT
GGGTTCCTCCTTAGATTACAAAGTAGGAACGATGATTGAGATCCCTAGAGCTGCTCTAGTAGCAGACCAGATTGCAGTGGAAGCAGATTTCTTCTCGTTTGGAACGAATGATCTTACACAGATGACATTTGGTTACAGTAGAGA
TGATGTTGGCAAGTTCCTCCCTATATACTTGTCACAGGGCATGCTGCAGACTGACCCCTTTGAGGTTCTCGACCAGAAAGGTGTAGGCCAACTCATCAAACTTGCAACTGAGAAAGGTCGTGCCGCAAAGCCTAGCTTGAAGGT
TGGCATATGCGGAGAACATGGAGGAGAGCCTTCTTCTGTCGCATTTTTTGTTGAGGCTGGACTTGATTATGTCTCATGTTCCCCATTCAGAGTTCCTATTGCAAGATTAGCTGCAGCTCAAGTTGCAGTTTGA

Figure S1: Systematic representation of genomic organization of *SmPPDK* gene sequence. The *SmPPDK* gene is comprised of six-exons and seven-introns (NCBI gene accession no.: MK704410)

```
1      **ATG**GCATTATCTTTCAAAGGAATGCTGGTCAGAACTCCTGATATATGCACACAGACACTTCTGAAAGGGAAGCACTTACATCAGATTGGTTTTTGAAGGACAAA
1       M  A  L  S  F  K  G  M  L  V  R  T  P  D  I  C  T  Q  T  L  L  K  G  K  H  L  H  Q  I  G  F  L  K  D  K

106    TATGAACTTGATTGTAGCCAATTCGATATTCGCCATGTTCAATTCCAGAACAGGAAACACCCACATCGACTTGGAAGACAATGCAAGTCACAAGTTGACATGGCT
36      Y  E  L  D  C  S  Q  F  D  I  R  H  V  Q  F  Q  N  R  K  H  P  H  R  L  G  R  Q  C  K  S  Q  V  D  M  A

211    CTGGTCTCAGAGCCAGTTCCAACCACCAAAAAGCGAGTATTTACTTTTGGTAAAGGAAGAAGTGATGGCGACAAGAGCATGAAGTCCTTGTTGGGAGGTAAAGGA
71      L  V  S  E  P  V  P  T  T  K  K  R  V  F  T  F  G  K  G  R  S  D  G  D  K  S  M  K  S  L  L  G  G  K  G

316    GCAAATCTCGCAGAAATGGCAAGTATAGGCTTATCCGTTCCTCCTGGGTTGACAATTTCTACAGAAGCATGCCAAGAATATCAGGATGTTGGGAAAAAGCTTCCC
106     A  N  L  A  E  M  A  S  I  G  L  S  V  P  P  G  L  T  I  S  T  E  A  C  Q  E  Y  Q  D  V  G  K  K  L  P

421    GAAAGTTTGTGGGAAGAAATACTTGAGGGTTTGAGGGTGATAGAGAATGACATGGGAGCGTACCTTGGAGACTCCTCAAAACCTCTTCTACTTTCAGTTCGTTCT
141     E  S  L  W  E  E  I  L  E  G  L  R  V  I  E  N  D  M  G  A  Y  L  G  D  S  S  K  P  L  L  L  S  V  R  S

526    GGTGCTGCAATTTCCATGCCAGGGATGATGGACACTGTCTTAAATCTTGGACTTAATGATGAAGTGGTAGTAGGGCTGGCAGCAAAAAGTGGAGAACGCTTTGCC
176     G  A  A  I  S  M  P  G  M  M  D  T  V  L  N  L  G  L  N  D  E  V  V  V  G  L  A  A  K  S  G  E  R  F  A

631    TATGACTCATTTAGGCGTTTCTTGGACATGTTTGGTGGTGTAGTCATGGGGATTCCTCACTCAGCATTCGAGGAGAAGTTAGAAAAGCTGAAACAAACCAAAGGA
211     Y  D  S  F  R  R  F  L  D  M  F  G  G  V  V  M  G  I  P  H  S  A  F  E  E  K  L  E  K  L  K  Q  T  K  G

736    GTTAGACTTGATACTGAACTAACTTCTTCCGATCTGAAAGAACTTGCAGAGCAATACAAAAATGTGTATATTGAAACAAAAGGAGAGGTTTTTCCAGCTGATCCA
246     V  R  L  D  T  E  L  T  S  S  D  L  K  E  L  A  E  Q  Y  K  N  V  Y  I  E  T  K  G  E  V  F  P  A  D  P

841    AAGAAACAGCTTCAGTTAGCAATTCAAGCGGTGTTTGATTCCTGGGACAGTCCAAGAGCTGTTAAATATAGAAGCATCAACCAGATCTCTGGGTTGAAAGGAACT
281     K  K  Q  L  Q  L  A  I  Q  A  V  F  D  S  W  D  S  P  R  A  V  K  Y  R  S  I  N  Q  I  S  G  L  K  G  T

946    GCAGTCAACATCCAGTCTATGGTATTTGGTAATATGGGCAATACTTCAGGAACAGGTGTTCTTTTTACTCGAAATCCAAGCACTGGTGAAAAGAAGCTCTATGGA
316     A  V  N  I  Q  S  M  V  F  G  N  M  G  N  T  S  G  T  G  V  L  F  T  R  N  P  S  T  G  E  K  K  L  Y  G

1051   GAGTTTTTAATTAATGCTCAGGGAGAAGATGTGGTCGCTGGCATTAGAACACCAGAAGACTTGGATGCCATGAAGAACTGCATGCCTGAAGCTTACAAAGAATTA
351     E  F  L  I  N  A  Q  G  E  D  V  V  A  G  I  R  T  P  E  D  L  D  A  M  K  N  C  M  P  E  A  Y  K  E  L

1156   GTTCAGAACTGTGAAATTTTAGAACAACATTACAAGGATATGATGGATATTGAATTCACCGTTCAAGAAAATAGGCTGTGGATGTTGCAATGCCGTTCTGGTAAG
386     V  Q  N  C  E  I  L  E  Q  H  Y  K  D  M  M  D  I  E  F  T  V  Q  E  N  R  L  W  M  L  Q  C  R  S  G  K

1261   CGTACTGGAGCAGGTGCGGTGAAGATTGCTGTAGATTTGGTGAATGAAGGAATTGTAAACTCCCGTGCTGCTGTTAAAATGGTGGAACCACAACATCTTGACCAA
421     R  T  G  A  G  A  V  K  I  A  V  D  L  V  N  E  G  I  V  N  S  R  A  A  V  K  M  V  E  P  Q  H  L  D  Q

1366   CTTCTTCATCCACAGTTCGAAGACCCATCTGCATATAAAGATAGTGTGATTGCCCGTGGACTGCCAGCATCTCCTGGAGCTGCTGTGGGGCAGATTGTATTCAGT
456     L  L  H  P  Q  F  E  D  P  S  A  Y  K  D  S  V  I  A  R  G  L  P  A  S  P  G  A  A  V  G  Q  I  V  F  S

1471   GCTGAGGATGCCGAAGCCTGGCATGCACAAGGGAAGAGTGCTATTCTGGTAAGGAATGAAACTAGCCCAGAGGATGTTGGGGGTATGCATGCAGCTGCTGGAATC
491     A  E  D  A  E  A  W  H  A  Q  G  K  S  A  I  L  V  R  N  E  T  S  P  E  D  V  G  G  M  H  A  A  A  G  I

1576   TTGACTGCTAGGGGAGGAATGACATCTCATGCTGCGGTCGTGGCTCGTGGCTGGGGAAAGTGTTGTGTTTCAGGGTGCTCTGAGATCCAAGTAAATGAAACCAAC
526     L  T  A  R  G  G  M  T  S  H  A  A  V  V  A  R  G  W  G  K  C  C  V  S  G  C  S  E  I  Q  V  N  E  T  N

1681   AAGGTTCTTGTGGCTGGAGATAAAGTCATAGCTGAAGGAGAATGGCTTTCTCTAAATGGGTCTACGGGTGAAGTGATCTTAGGGAAACAGCCACTTGCACCGCCT
561     K  V  L  V  A  G  D  K  V  I  A  E  G  E  W  L  S  L  N  G  S  T  G  E  V  I  L  G  K  Q  P  L  A  P  P

1786   GCGCTCAGTGGAGATTTAGAAACCGTGATGTCTTGGGCTGATGCCGTACGACGTATTAAGGTGATGGCCAATGCAGATACACCAGAGGATGCATTGACAGCAAGA
596     A  L  S  G  D  L  E  T  V  M  S  W  A  D  A  V  R  R  I  K  V  M  A  N  A  D  T  P  E  D  A  L  T  A  R

1891   AACAATGGTGCCCAAGGGATTGGCCTTTGCCGGACTGAGCACATGTTTTTTGCTTCAGATGATAGGATTAAGACAGTGAGGAAAATGATAATGGCAGTTACACCT
631     N  N  G  A  Q  G  I  G  L  C  R  T  E  H  M  F  F  A  S  D  D  R  I  K  T  V  R  K  M  I  M  A  V  T  P

1996   GAGCAGAGGAAAGCAGCTTTGGATCAGTTATTACCTTACCAGAGATCTGATTTTGAAGGGATTTTTCGAGCCATGGATGGGCTTCCTGTGACAATAAGATTGCTA
666     E  Q  R  K  A  A  L  D  Q  L  L  P  Y  Q  R  S  D  F  E  G  I  F  R  A  M  D  G  L  P  V  T  I  R  L  L

2101   GACCCTCCATTGCATGAGTTTCTTCCAGAAGGTGATTTGGAACAAATTGTCAATGAGTTGGCCTCCGAAACTGGAGTGAACAAAGATGAAGTCTTTTCCAGGATA
701     D  P  P  L  H  E  F  L  P  E  G  D  L  E  Q  I  V  N  E  L  A  S  E  T  G  V  N  K  D  E  V  F  S  R  I

2206   GAAAAGCTATCTGAAGTTAATCCCATGTTGGGCTTCAGGGGTTGCAGGTTAGGGATATCATATCCCGAACTAACTGAAATGCAAGCACGTGCAGTATTTCAAGCT
736     E  K  L  S  E  V  N  P  M  L  G  F  R  G  C  R  L  G  I  S  Y  P  E  L  T  E  M  Q  A  R  A  V  F  Q  A

2311   GCAGTTTCAATGAGCAACCAGGGATTTACGGTTCTACCTGAAATCATGGTTCCTCTCGTTGGAACACCTCAGGAGTTAGGGAATCAAGTGAGTTTGATACGAAAT
771     A  V  S  M  S  N  Q  G  F  T  V  L  P  E  I  M  V  P  L  V  G  T  P  Q  E  L  G  N  Q  V  S  L  I  R  N

2416   GTTGCAACAAATGTATTCTCAGAGATGGGTTCCTCCTTAGATTACAAAGTAGGAACGATGATTGAGATCCCTAGAGCTGCTCTAGTAGCAGACCAGATTGCAGTG
806     V  A  T  N  V  F  S  E  M  G  S  S  L  D  Y  K  V  G  T  M  I  E  I  P  R  A  A  L  V  A  D  Q  I  A  V

2521   GAAGCAGATTTCTTCTCGTTTGGAACGAATGATCTTACACAGATGACATTTGGTTACAGTAGAGATGATGTTGGCAAGTTCCTCCCTATATACTTGTCACAGGGC
841     E  A  D  F  F  S  F  G  T  N  D  L  T  Q  M  T  F  G  Y  S  R  D  D  V  G  K  F  L  P  I  Y  L  S  Q  G

2626   ATGCTGCAGACTGACCCCTTTGAGGTTCTCGACCAGAAAGGTGTAGGCCAACTCATCAAACTTGCAACTGAGAAAGGTCGTGCCGCAAAGCCTAGCTTGAAGGTT
876     M  L  Q  T  D  P  F  E  V  L  D  Q  K  G  V  G  Q  L  I  K  L  A  T  E  K  G  R  A  A  K  P  S  L  K  V

2731   GGCATATGCGGAGAACATGGAGGAGAGCCTTCTTCTGTCGCATTTTTTGTTGAGGCTGGACTTGATTATGTCTCATGTTCCCCATTCAGAGTTCCTATTGCAAGA
911     G  I  C  G  E  H  G  G  E  P  S  S  V  A  F  F  V  E  A  G  L  D  Y  V  S  C  S  P  F  R  V  P  I  A  R

2836   TTAGCTGCAGCTCAAGTTGCAGTT**TGA**                     ORF: 2863 bp       Protein: 953 aa
946     L  A  A  A  Q  V  A  V  *
```

Figure S2: Full length cDNA/ORF of the *SmPPDK* gene

L1

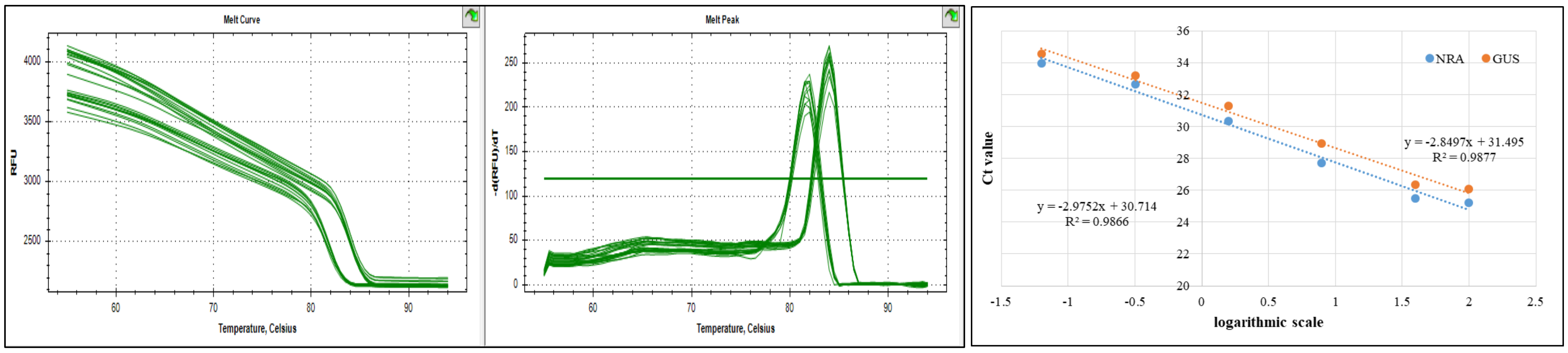

L20

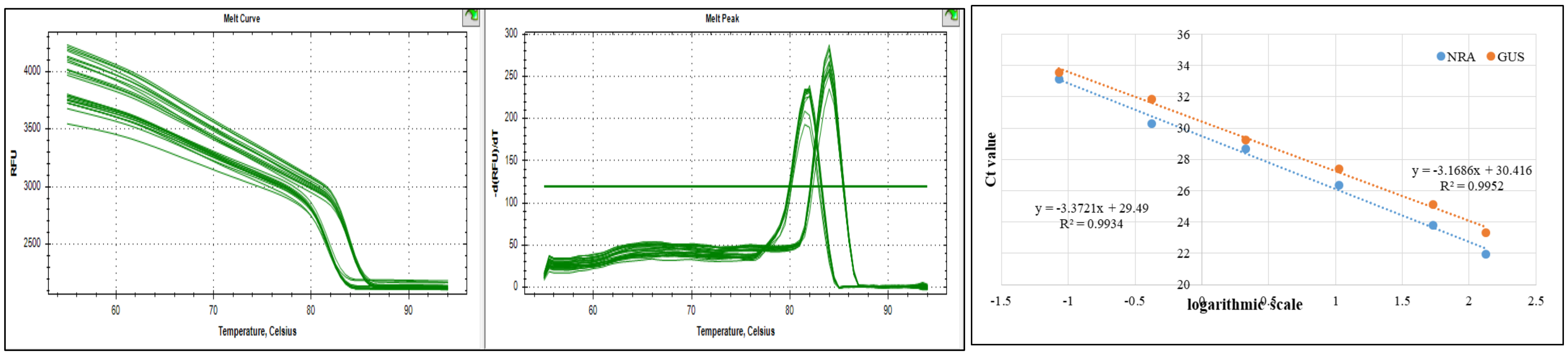

L32

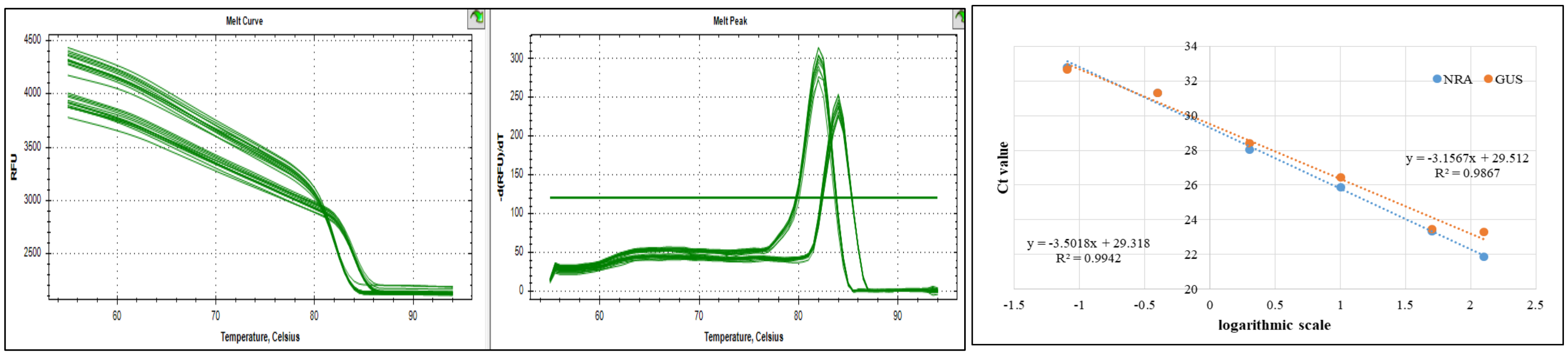

L33

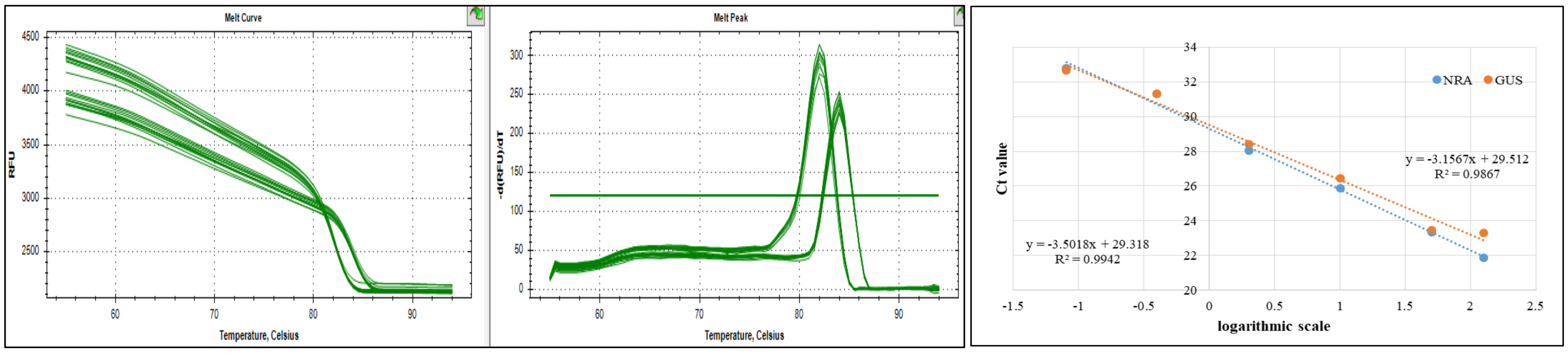

L40

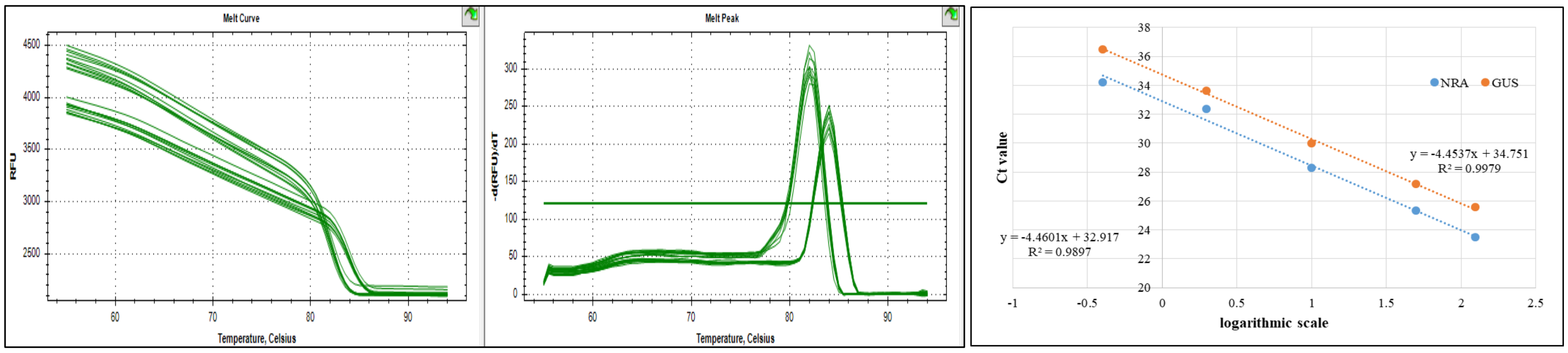

Copy number calculation for transgene ↓

Transgenic line	Efficiency of *GUS* RT- qPCR	Efficiency of *NRA* RT- qPCR	Copy no. ratio (*GUS:NRA*)	Transgene copy no. calculated
**L1**	1.168262	1.243438	1.034671	1
**L20**	0.97948	1.06822	1.04483	1
**L32**	1.021483	1.089395	1.033595	1
**L33**	0.930045	1.073894	1.074531	1
**L40**	0.675754	0.676997	1.000742	1

Figure S3: Determination of transgene integration event by real time qPCR

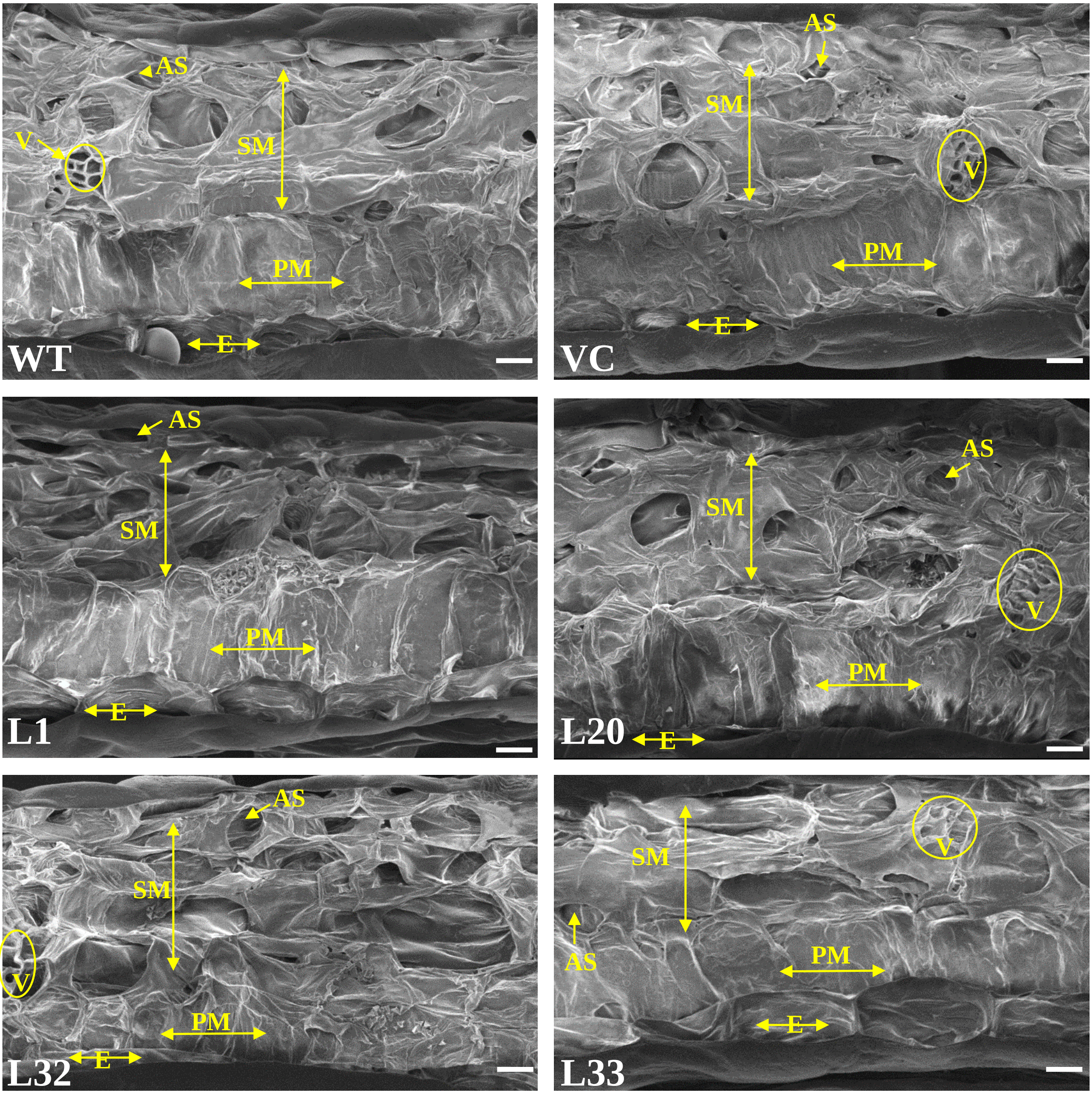

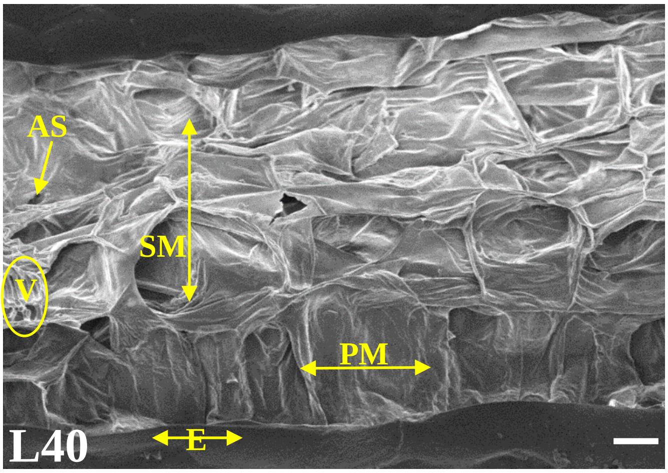

E: Epidermis
(upper & lower)
PM: Palisade Mesophyll
SM: Spongy Mesophyll
V: Vein
AS: Air Space
(in multiple numbers)

Figure S4: Scanning electron microscopy (SEM) of leaves of control and transformed plants showing ultrastructure of mesophyll cells

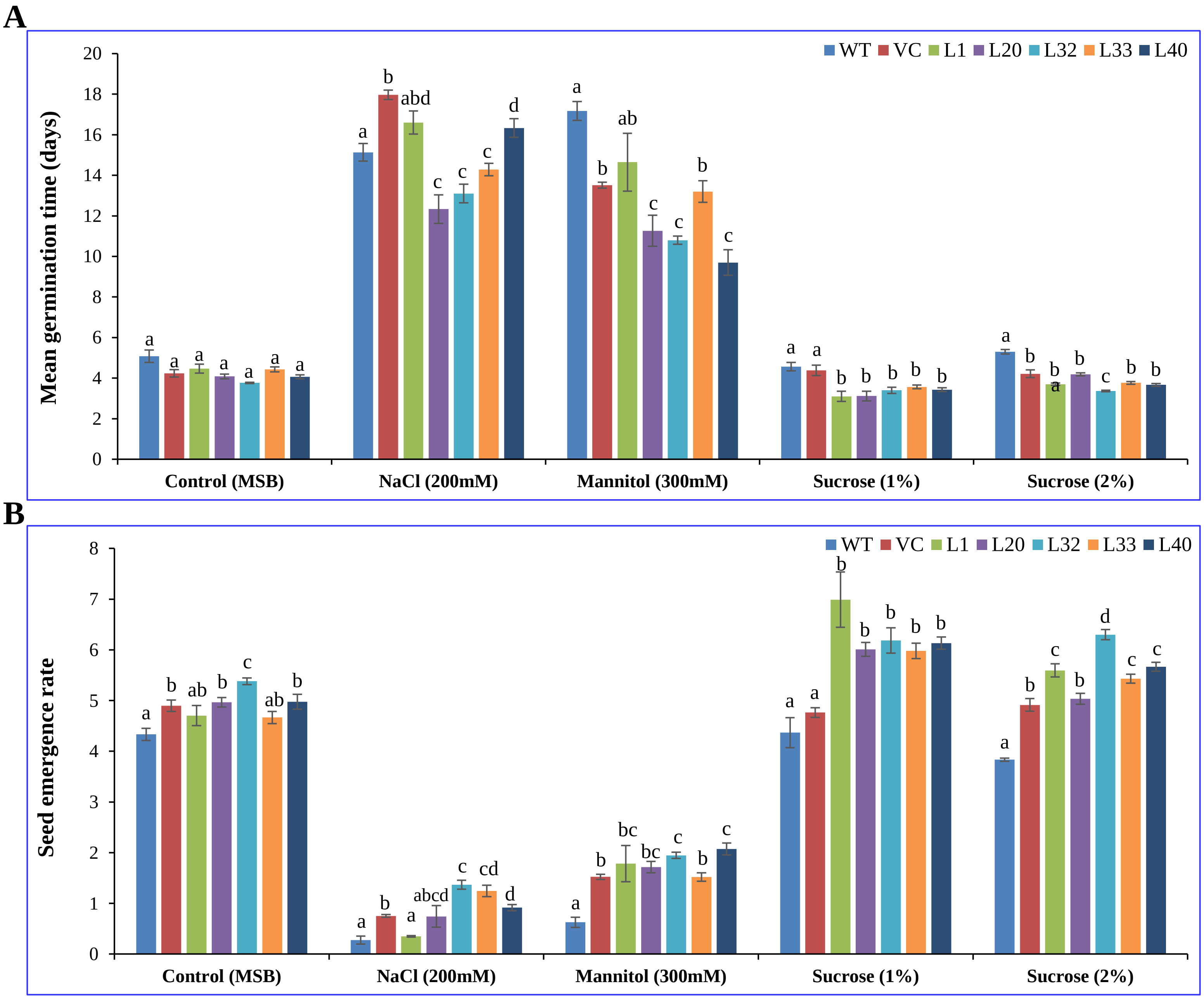

Figure S5: Seed germination study. Mean germination time (A) and seed emergence rate (B) of transgenic tobacco lines along with WT and VC plants were determined under different abiotic stress conditions. Bars represent means ± *SE* and values with different letters are significant at $P < 0.05$

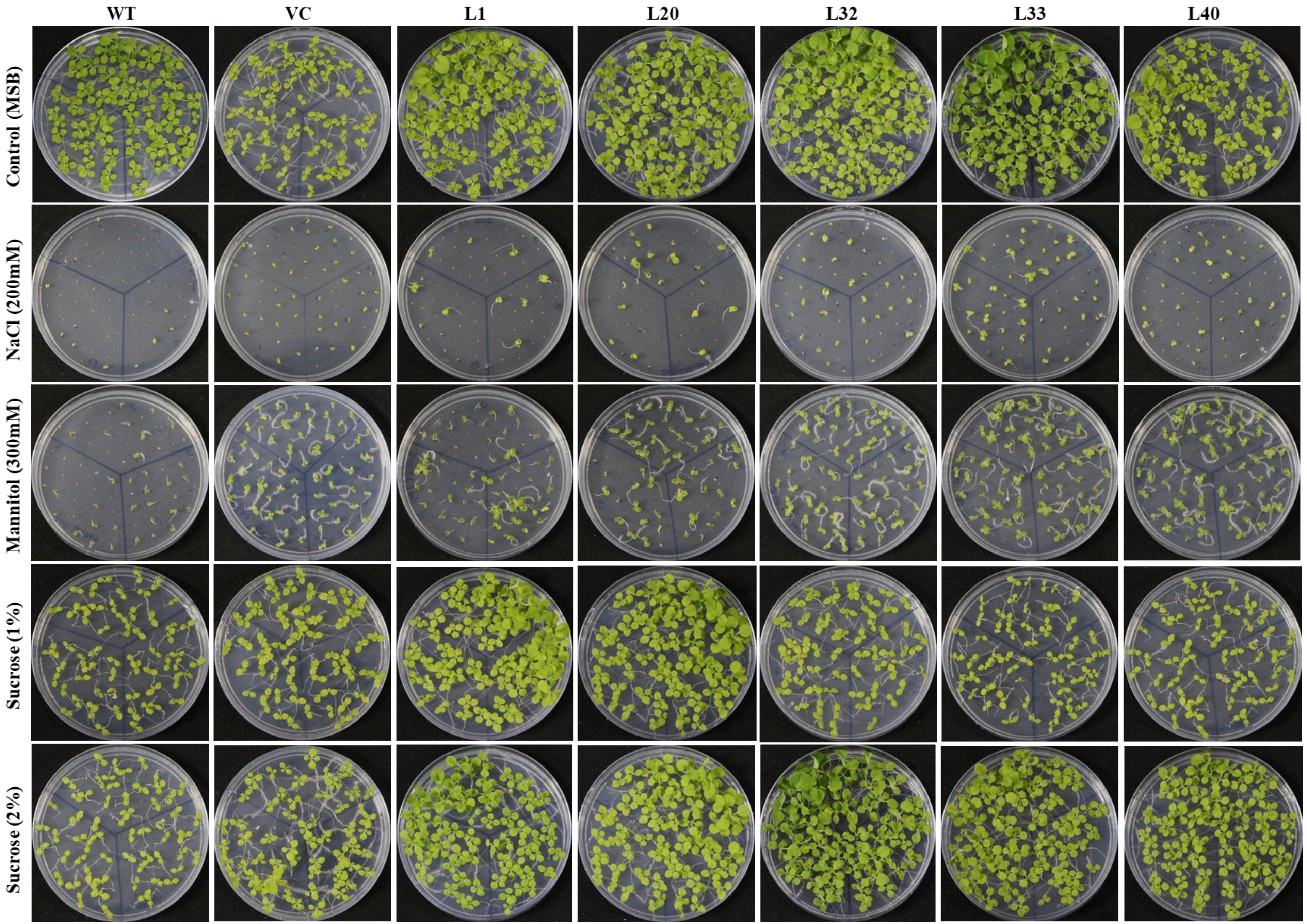

Figure S6: Comparative seed germination study of transgenic lines under different abiotic stress conditions

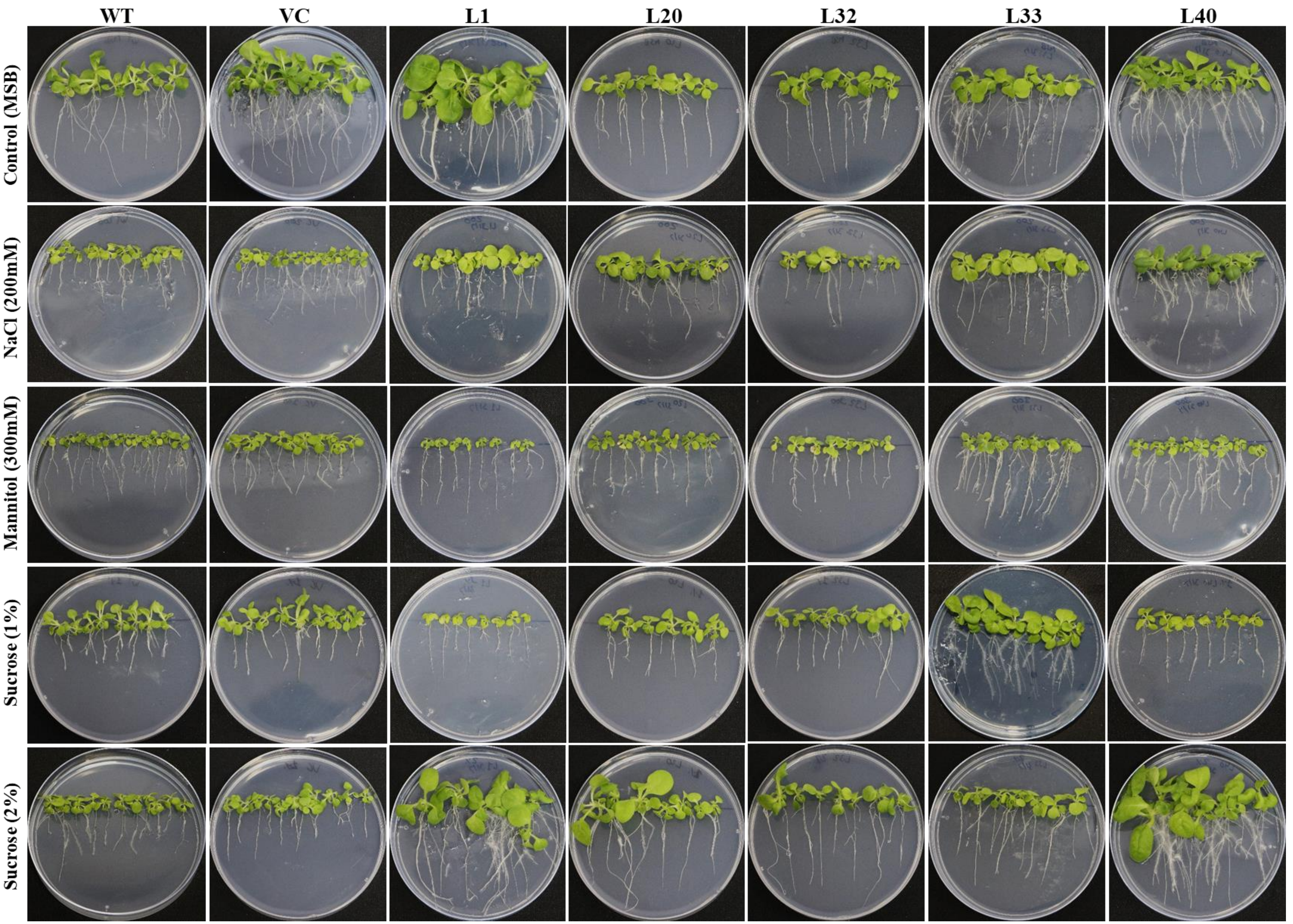

Figure S7: Comparative seedling growth study of transgenic lines under different abiotic stress conditions

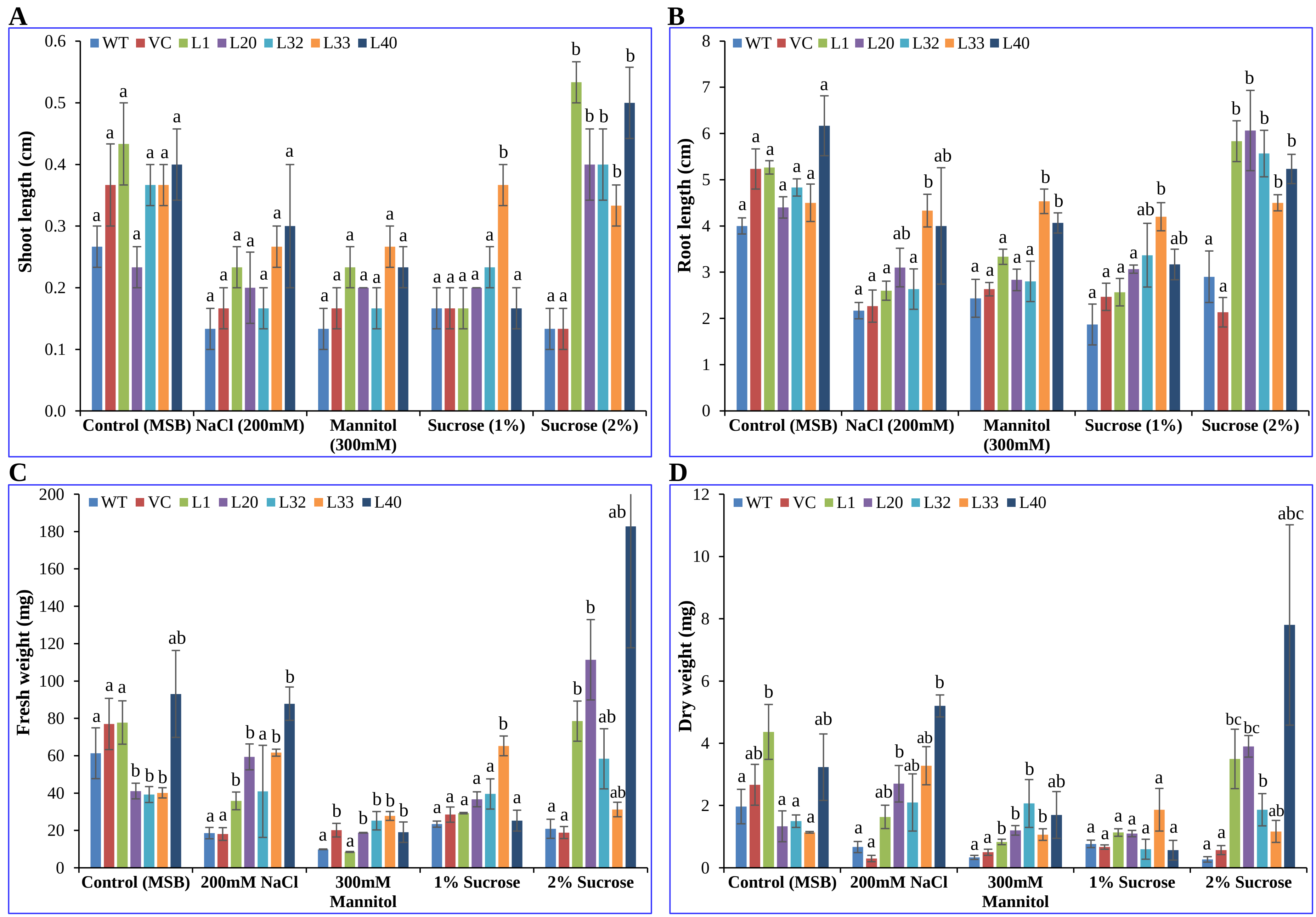

Figure S8: Plant growth parameter analysis. Measurement of shoot length (A), root length (B), fresh weight (C) and dry weight (D) of transgenic lines under different abiotic stress conditions. Bars represent means ± *SE* and values with different letters are significant at $P < 0.05$

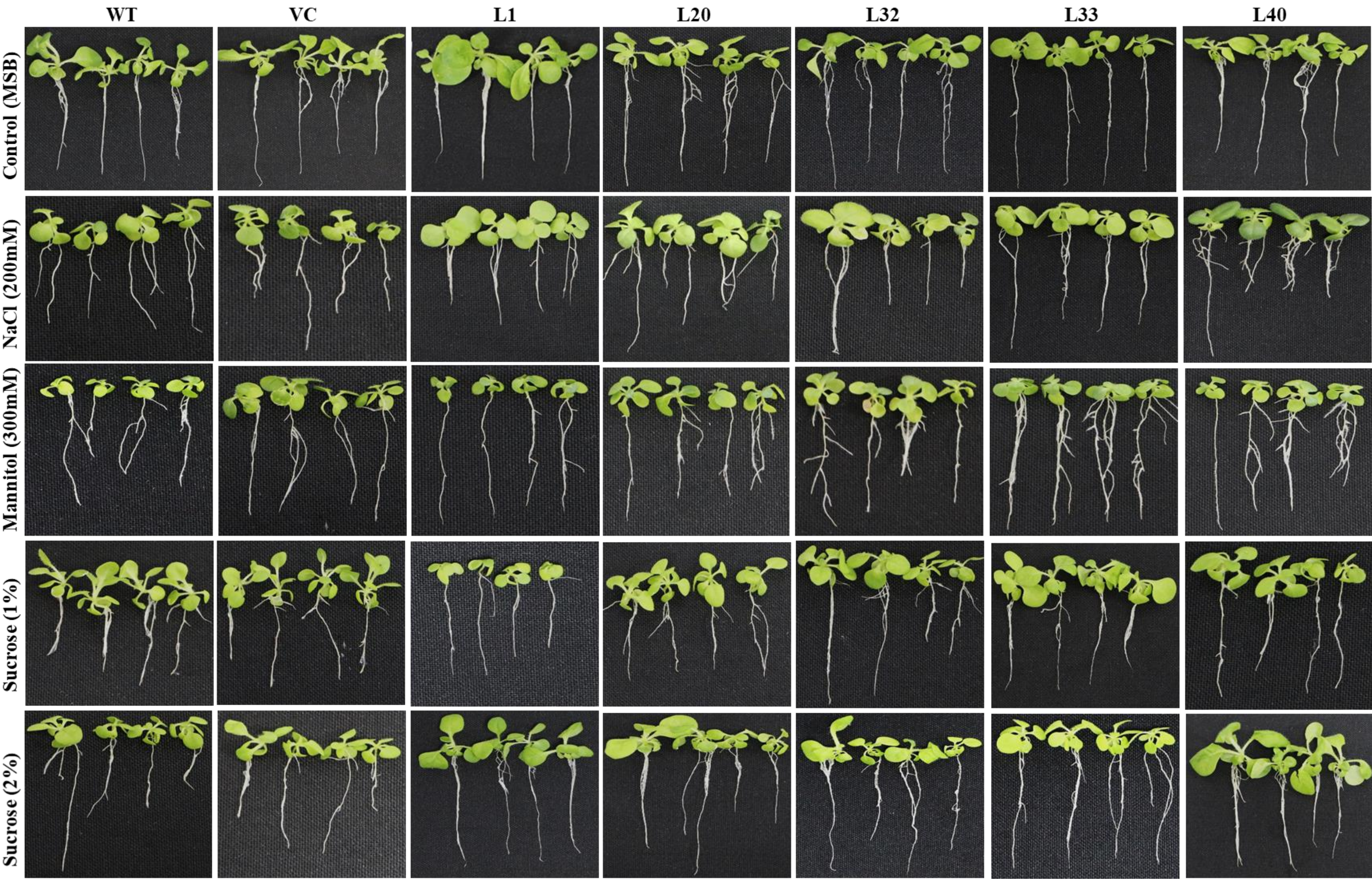

Figure S9: Comparative plant growth study of transgenic lines under different abiotic stress conditions

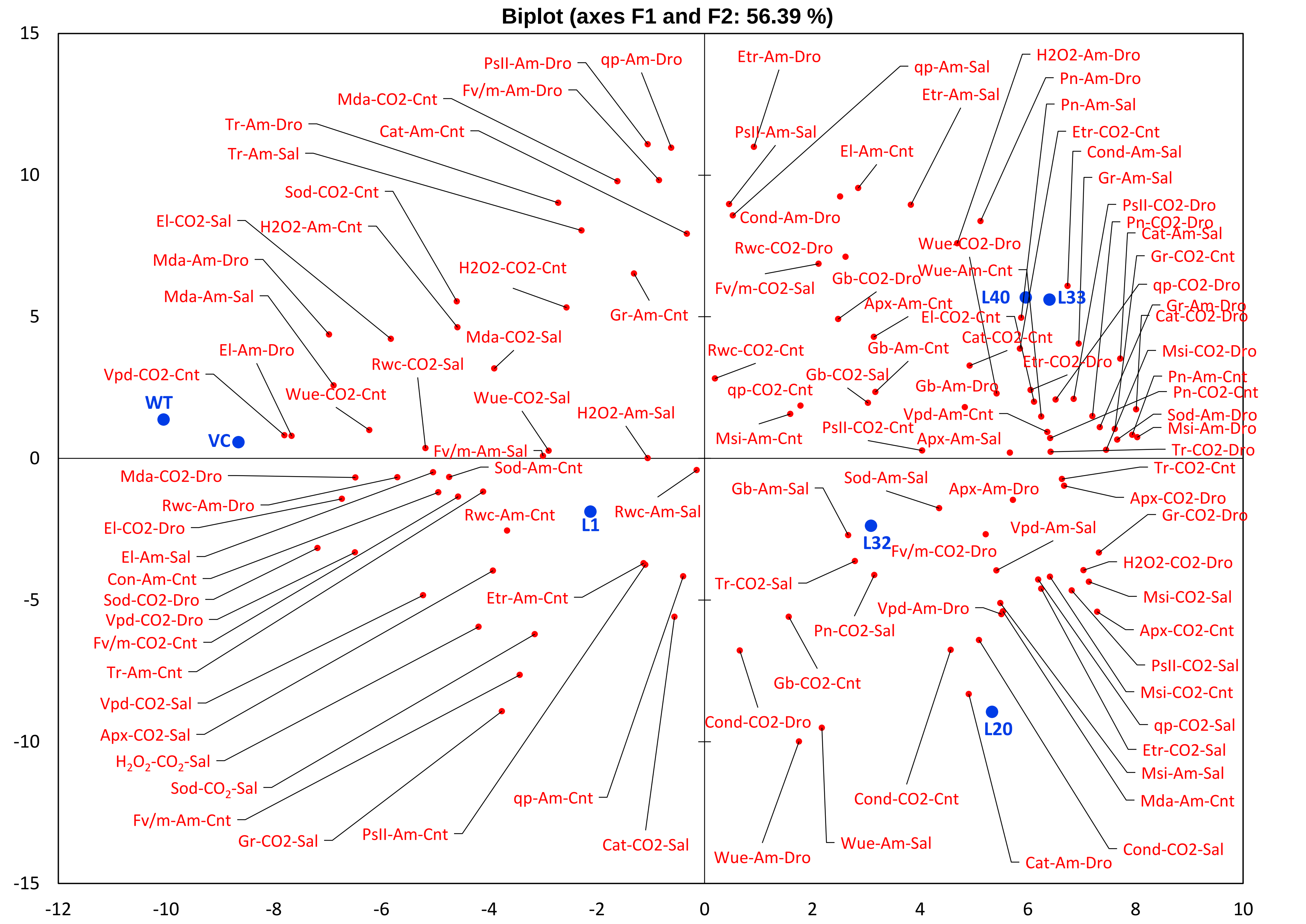

Figure S10: Principal component analysis. A Bi-plot of physiological and biochemical parameters of control and transgenic plants grown under ambient and elevated CO_2 environment

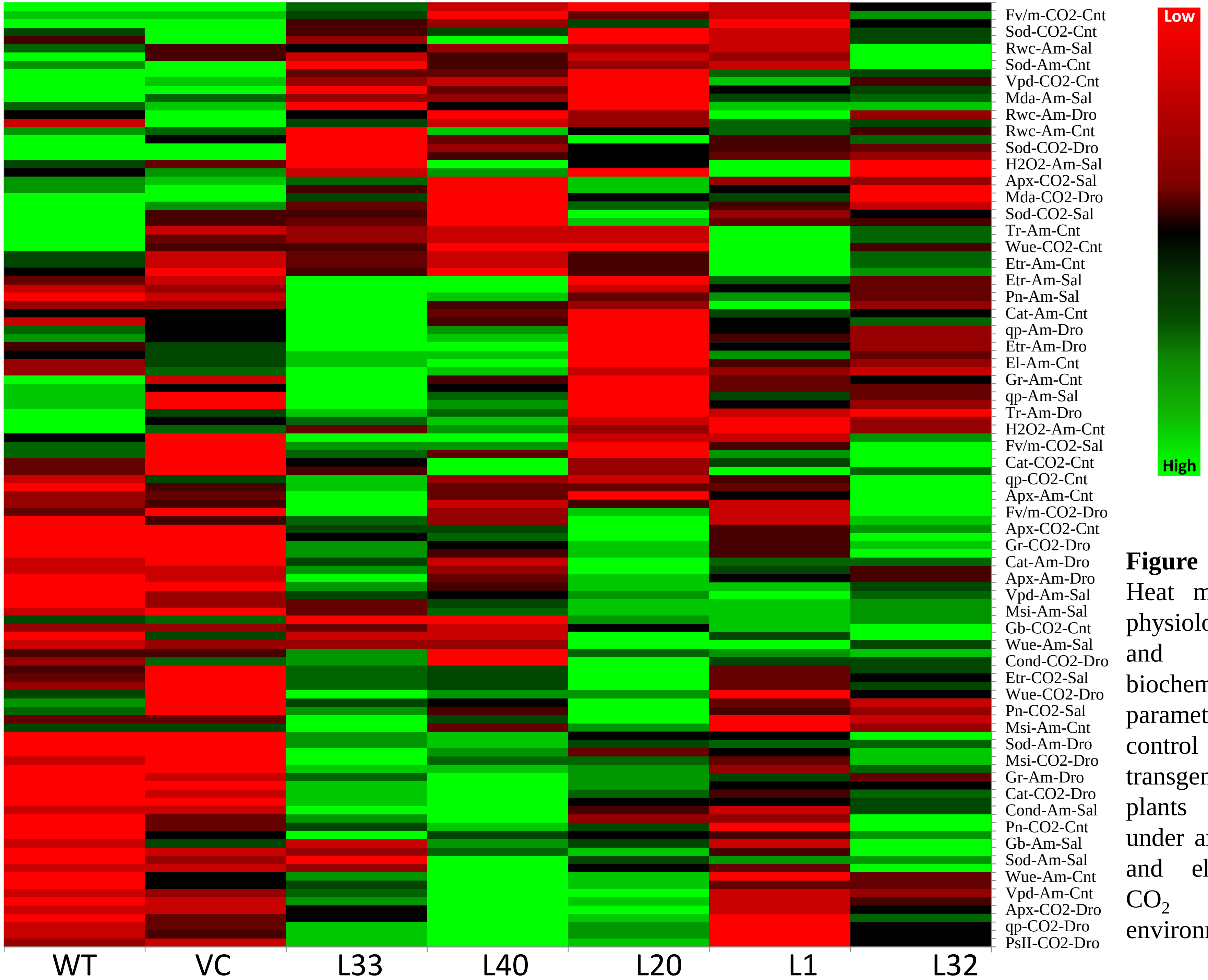

Figure S11: Heat map of physiological and biochemical parameters of control and transgenic plants grown under ambient and elevated CO_2 environment

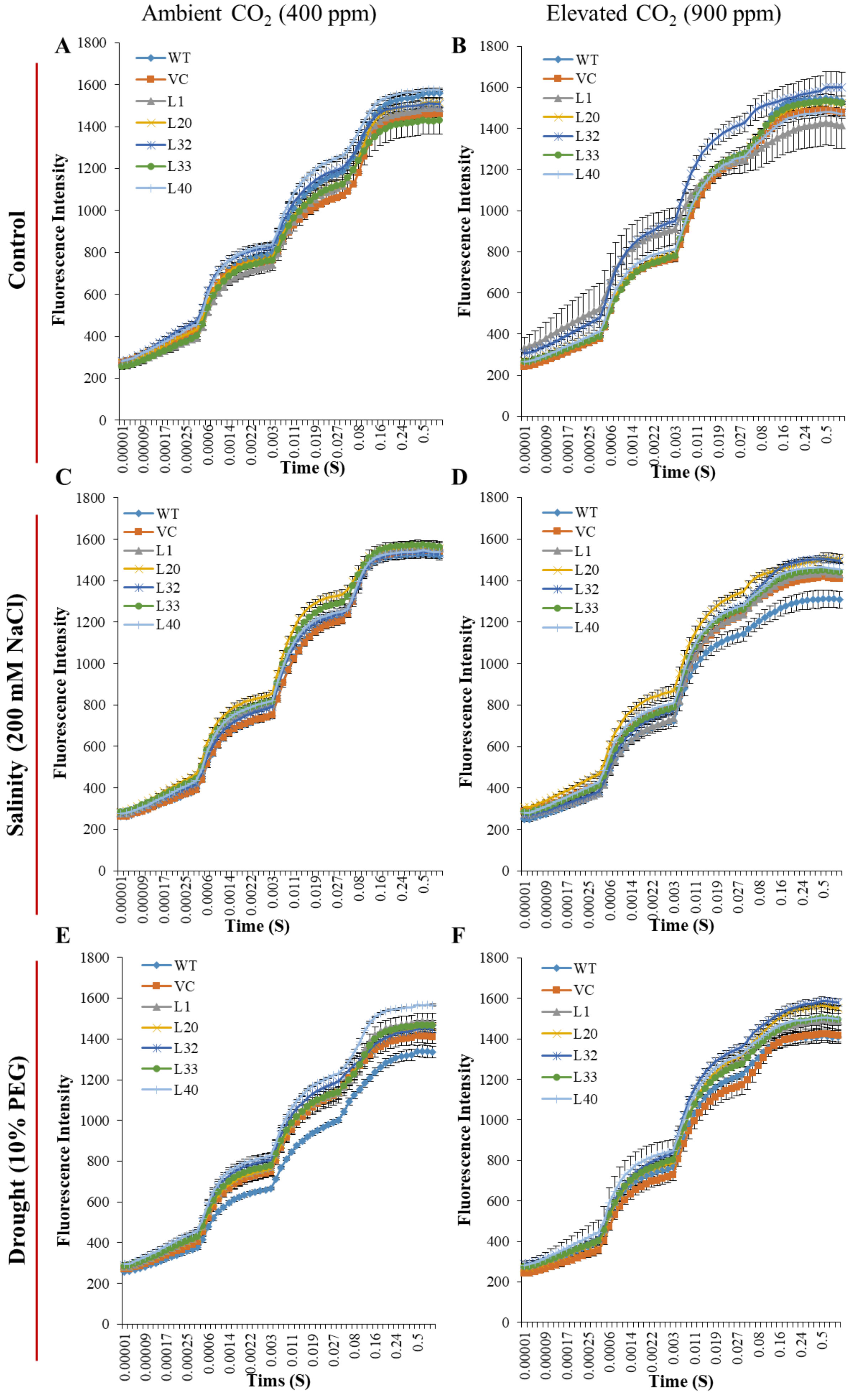

Figure S12: Fluorescence transient curve (OJIP) analysis of transgenic lines under different abiotic stress conditions

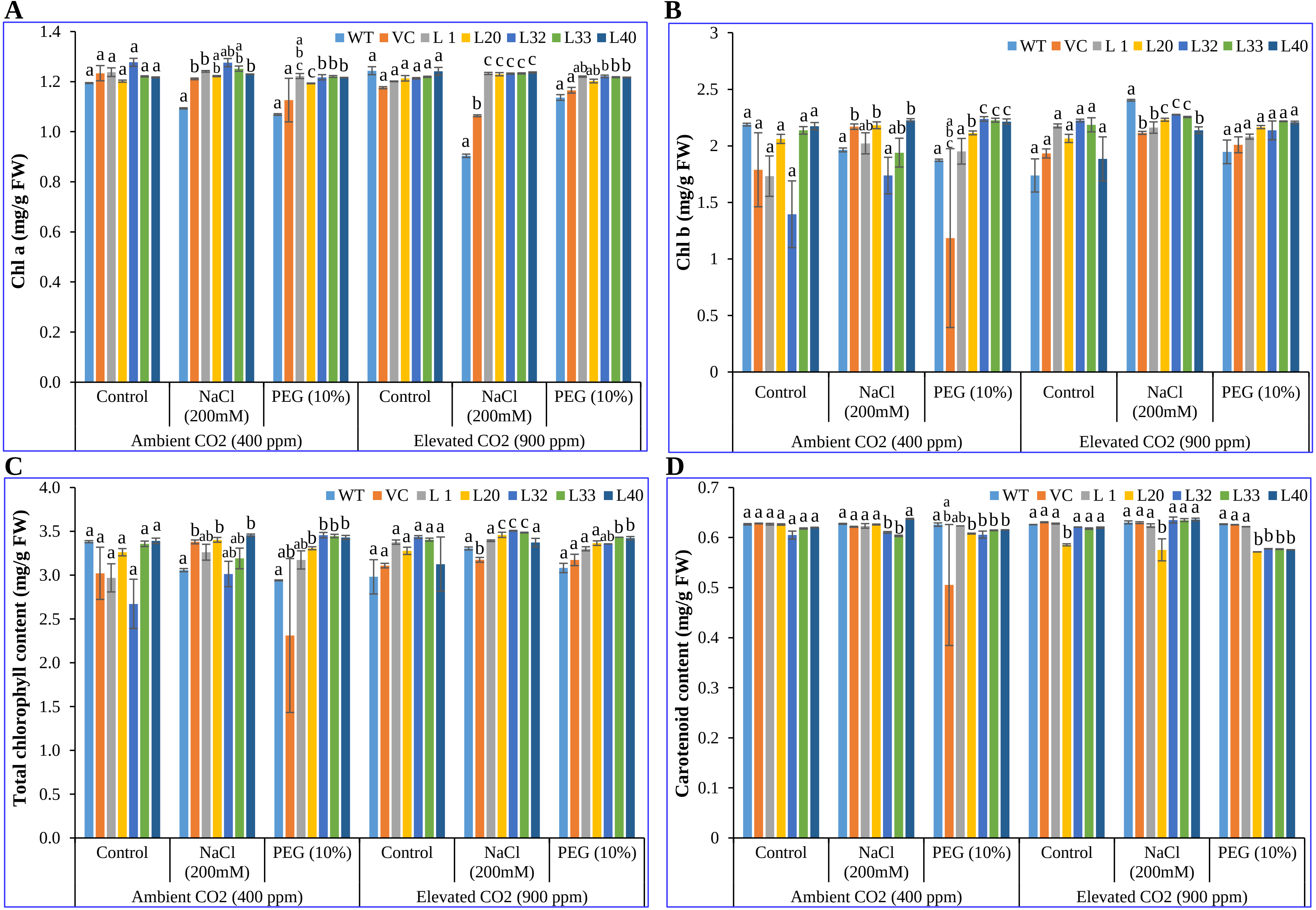

Figure S13: Estimation of chlorophyll and carotenoids contents. Chlorophyll a (A), chlorophyll b (B), total chlorophyll (C) and carotenoids (D) contents of WT, VC, and T1 transgenic lines (L1, L20, L32, L33 and L40) were measured under salinity (200 mM NaCl) and osmotic (10% PEG) stress condition grown in ambient and elevated CO_2 environment. Bars represent means ± *SE* and values with different letters are significant at $P < 0.05$

A

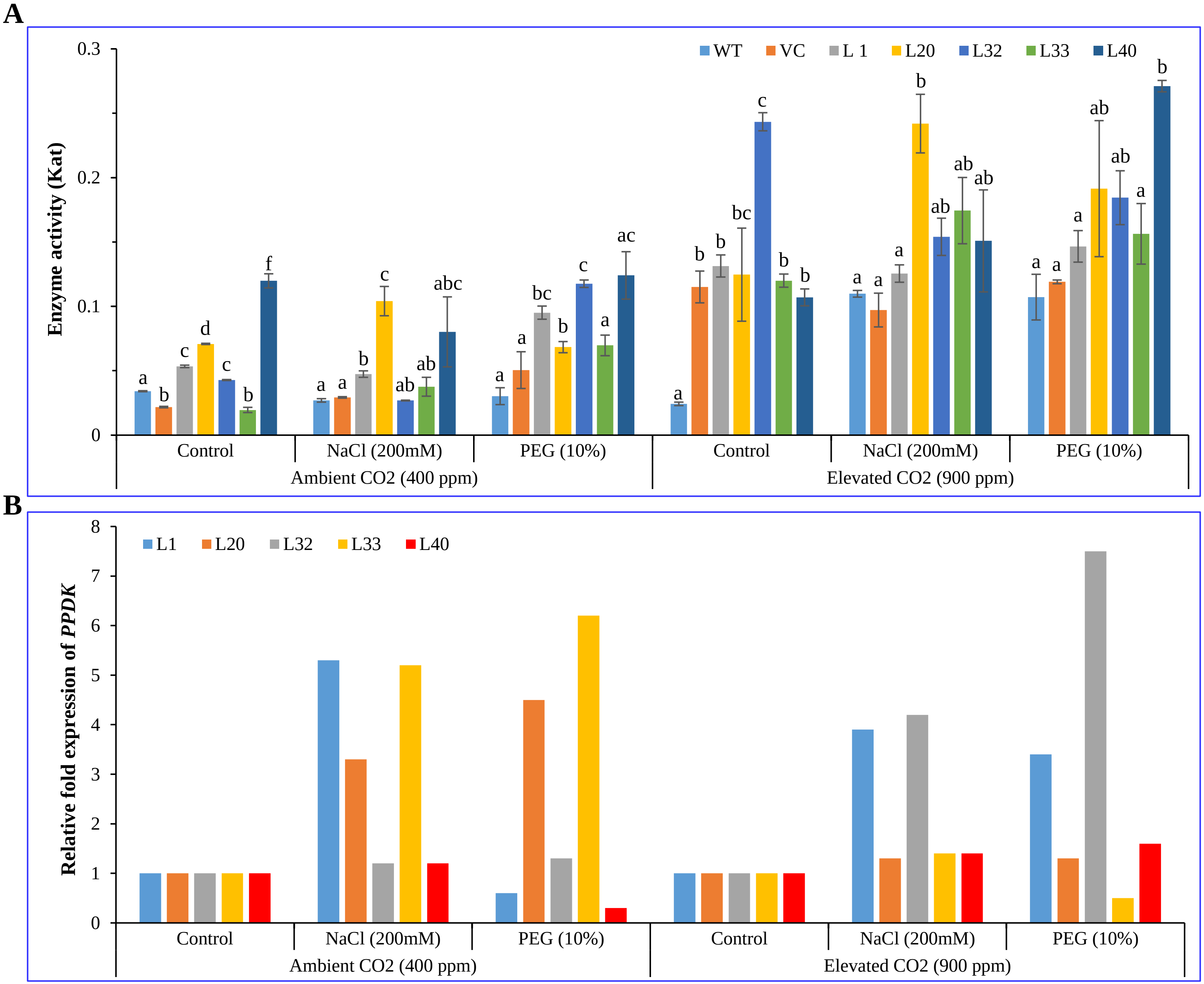

Figure S14: Pyruvate phosphate dikinase activity in transgenic lines. *Sm*PPDK enzyme activity (A) and *SmPPDK* transcript expression (B) of WT, VC, and T1 transgenic lines (L1, L20, L32, L33 and L40) were measured under salinity (200 mM NaCl) and osmotic (10% PEG) stress condition grown in ambient and elevated CO_2 environment. Bars represent means ± *SE* and values with different letters are significant at $P < 0.05$

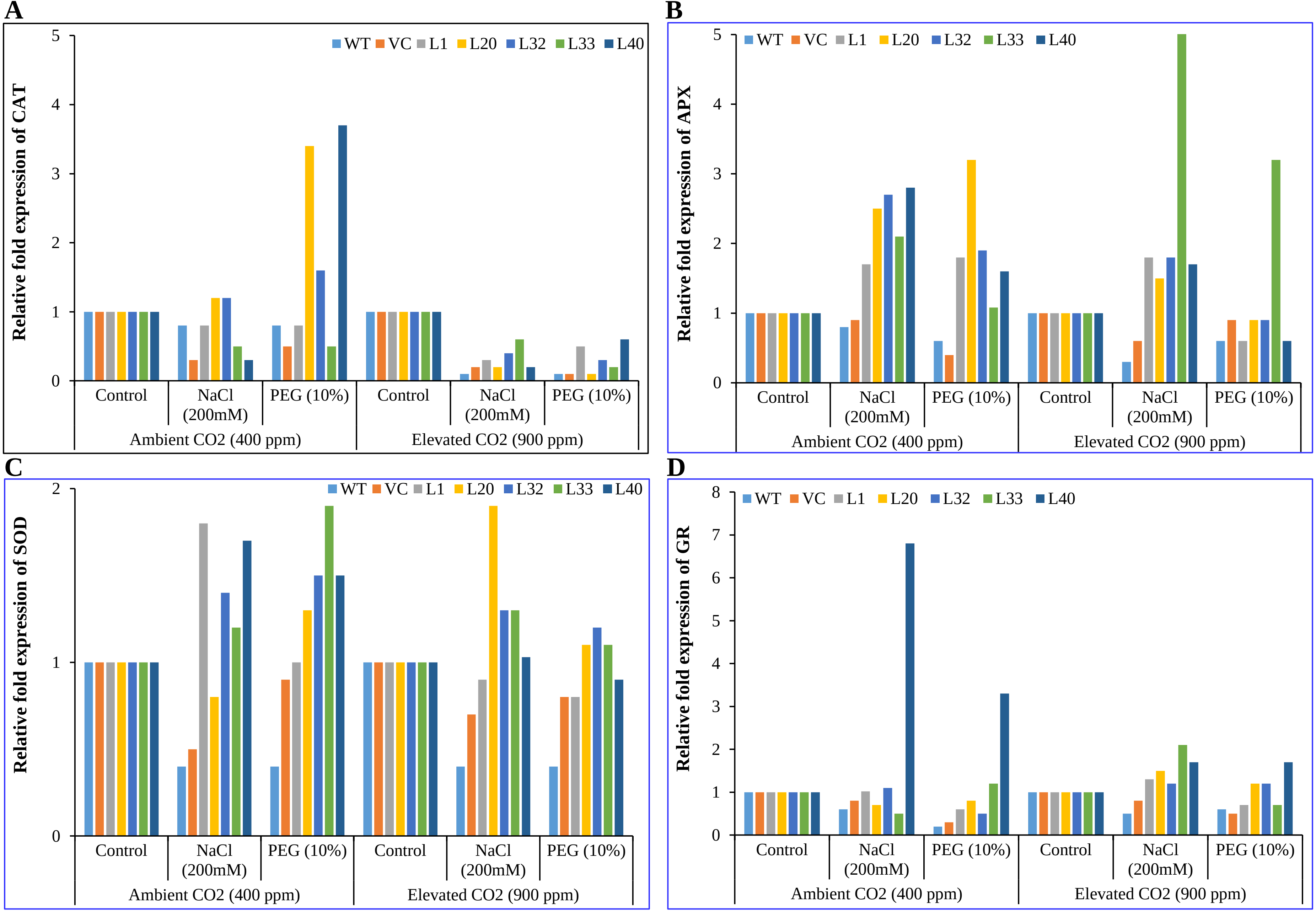

Figure S15: Transcript expression analysis of ROS scavenging enzyme encoding gene in transgenic lines. Estimation of relative gene expression of catalase (A), ascorbate peroxidase (B), superoxide dismutase (C) and glutathione reductase (D) genes in WT, VC, and T1 transgenic lines (L1, L20, L32, L33 and L40) were measured under salinity (200 mM NaCl) and osmotic (10% PEG) stress condition grown in ambient and elevated CO_2 environment

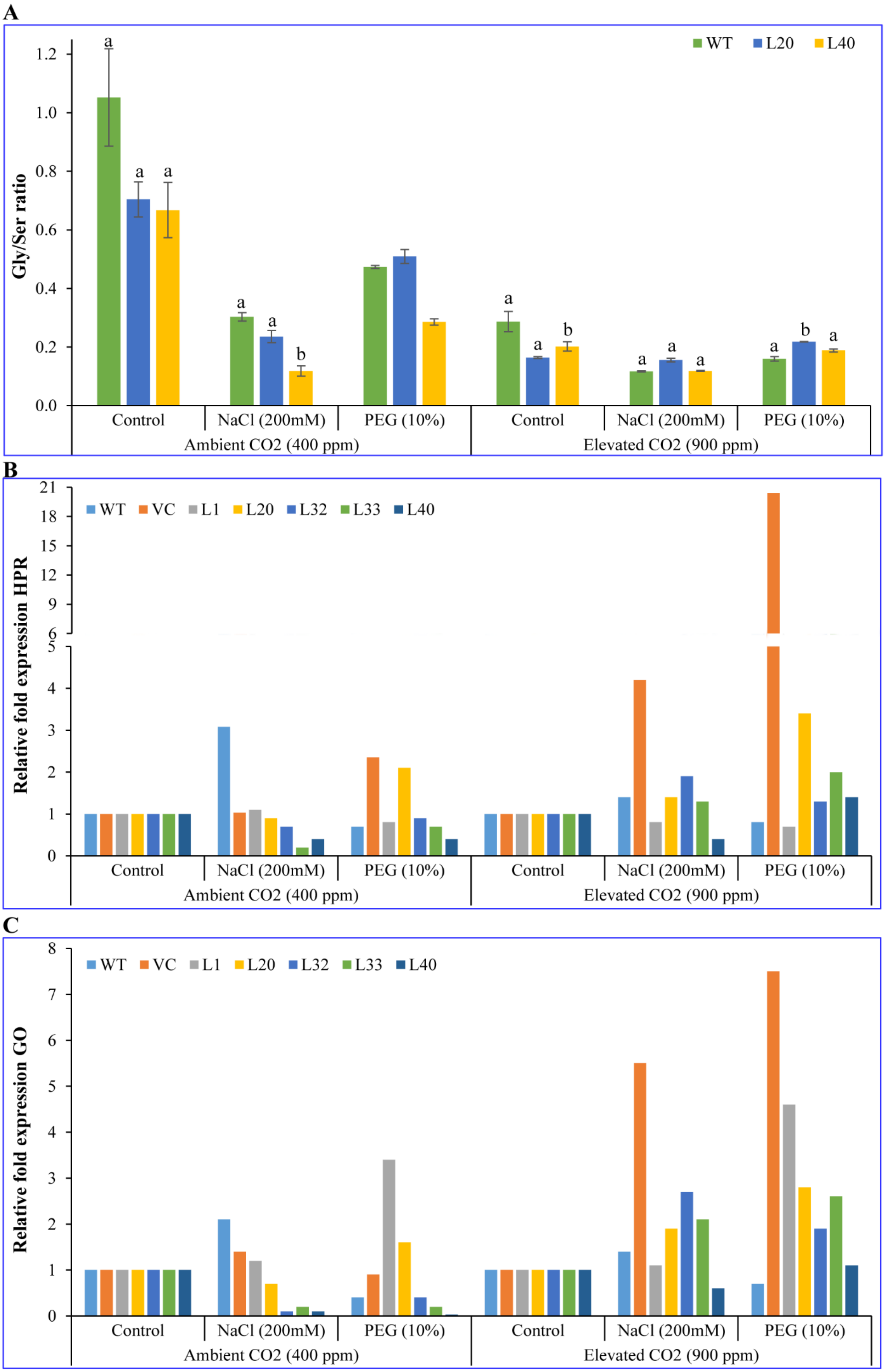

Figure S16: Study of photorespiration response in transgenic lines. (A) Glycine/Serine ratio was measured in two selected transgenic lines and WT leaves, transcript expression analysis of hydroxypyruvate reductase (*NtHPR*) gene (B) and glycolate oxidase (*NtGO*) gene (C) of WT, VC, and T1 transgenic lines (L1, L20, L32, L33 and L40) under salinity (200 mM NaCl) and osmotic (10% PEG) stress condition grown in ambient and elevated CO_2 environment. Bars represent means ± *SE* and values with different letters are significant at $P < 0.05$

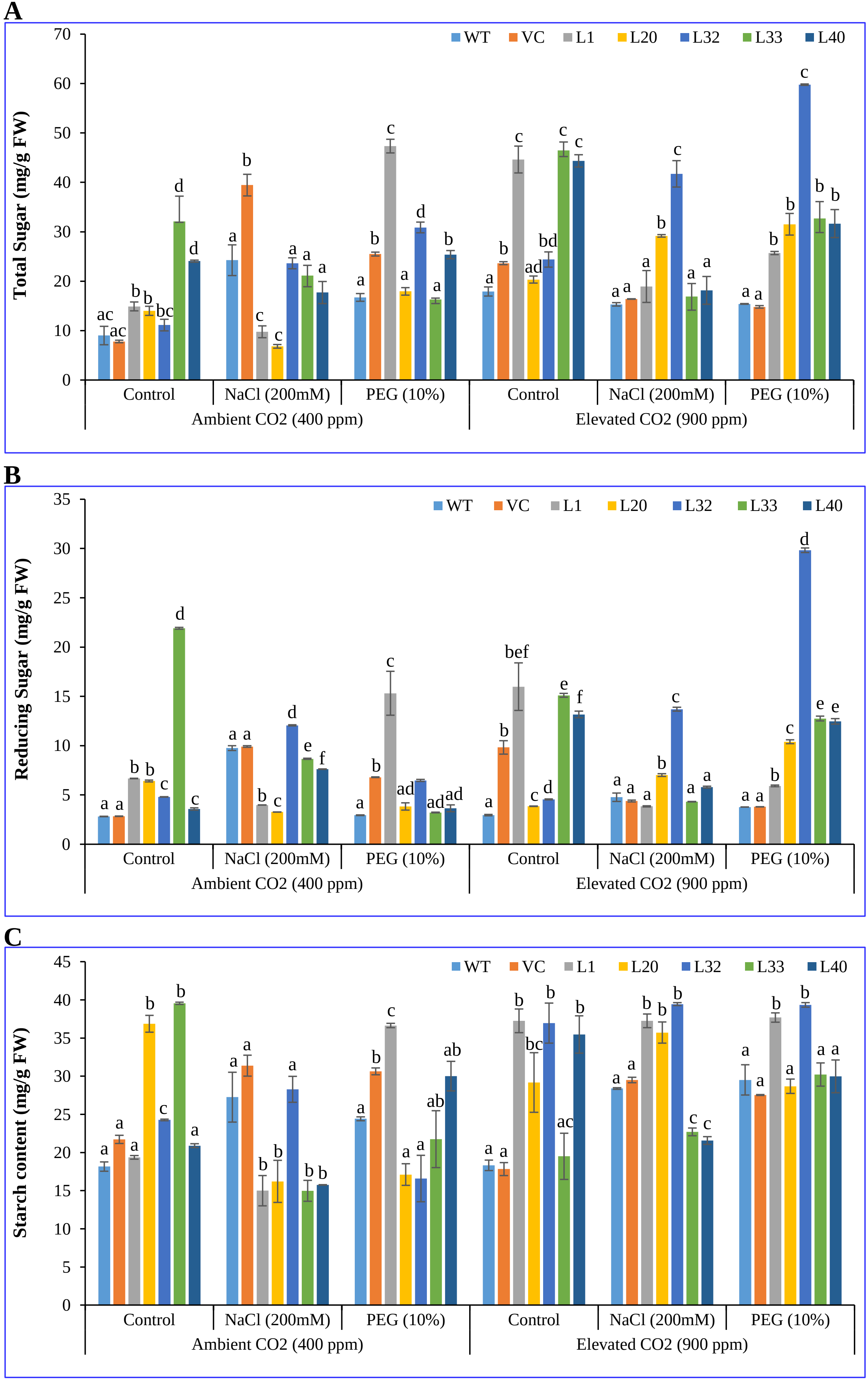

Figure S17: Determination of sugar content in transgenic lines. Total sugar (A), reducing sugar (B) and starch content (C) were measured under salinity (200 mM NaCl) and osmotic (10% PEG) stress condition grown in ambient and elevated CO_2 environment. Bars represent means ± *SE* and values with different letters are significant at $P < 0.05$

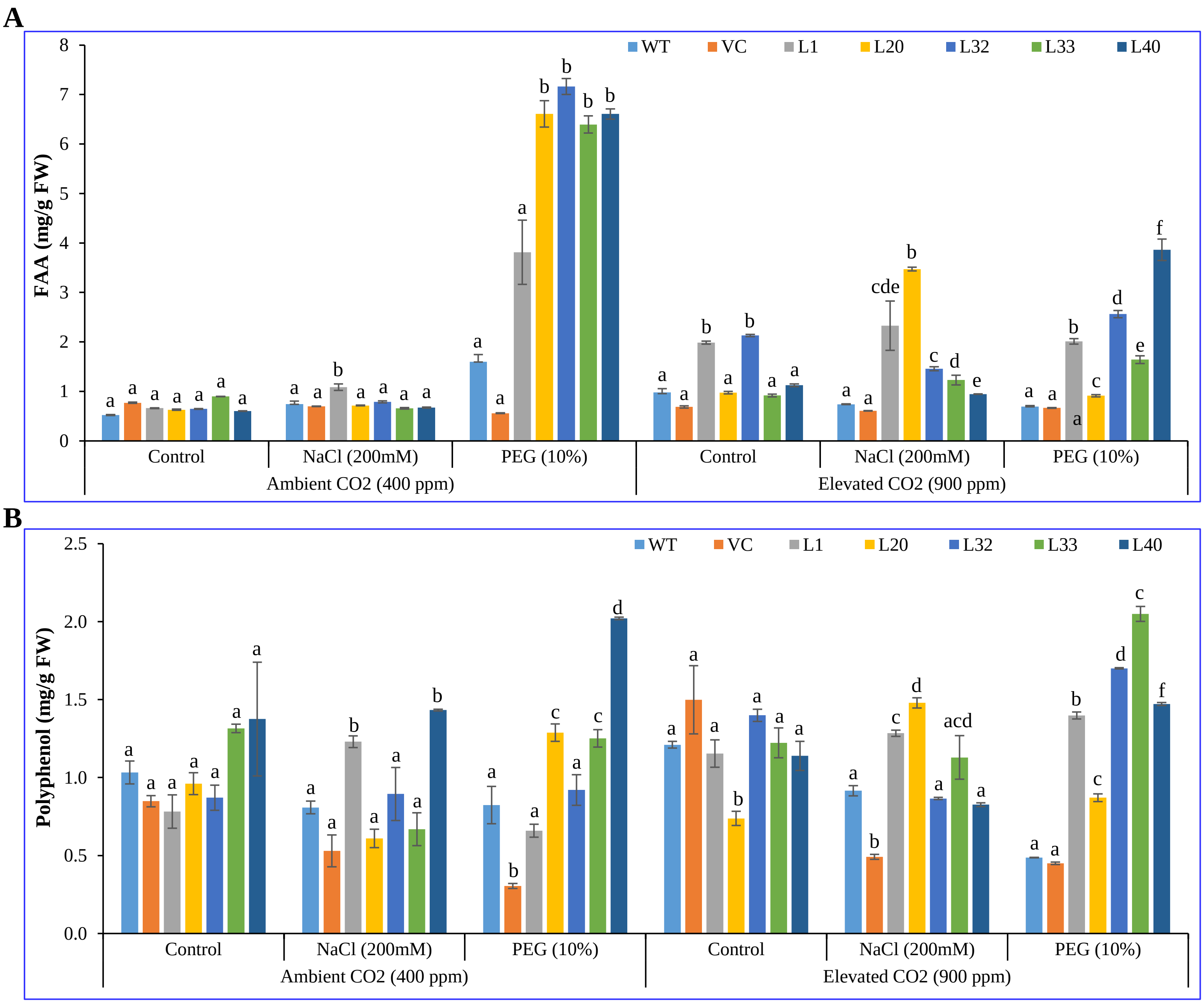

Figure S18: Determination of free amino acids and polyphenols in transgenic lines. FAA (A) and polyphenols were measured under salinity (200 mM NaCl) and osmotic (10% PEG) stress condition grown in ambient and elevated CO_2 environment. Bars represent means ± *SE* and values with different letters are significant at $P < 0.05$

Table S2: Identified different metabolites in the leaf of WT and two selected transgenic lines (L20 and L40) under salt (200 mmol L^{-1} NaCl) and drought (10% PEG) treatment for 24 hr under ambient CO_2 (400 ppm) condition. The relative concentration of each group of metabolites in control and stress condition is a mean of data from two biological replicates.

Categories	Metabolites	Control			Salt (200 mmol L^{-1} NaCl)			Drought (10% PEG)		
		WT	L20	L40	WT	L20	L40	WT	L20	L40
		Metabolites Concentration (µg/g) FW								
Amino acids	Asparagine	0.48±0.02	0.36±0.01	0.74±0.01	0.40±0.01	1.10±0.02	0.44±0.01	0.64±0.01	---	0.33±0.01
	Glycine	0.96±0.02	0.82±0.01	0.43±0.01	0.57±0.01	0.32±0.01	0.34±0.01	0.86±0.01	0.52±0.01	0.49±0.01
	L-Aspartic acid	6.46±0.07	7.79±0.15	2.33±0.03	3.90±0.03	6.32±0.02	7.22±0.04	7.05±0.01	4.40±0.02	4.44±0.02
	L-Glutamic acid	4.02±0.01	6.34±0.08	2.25±0.01	4.37±0.04	3.99±0.07	7.60±0.04	2.63±0.01	4.83±0.03	3.48±0.03
	L-Glutamine	1.36±0.05	0.59±0.01	0.93±0.01	1.05±0.01	2.89±0.08	0.94±0.01	6.24±0.03	0.87±0.01	2.54±0.03
	L-Methylalanine	0.63±0.02	0.76±0.01	---	---	0.41±0.01	0.74±0.01	---	0.93±0.01	0.68±0.01
	L-Proline	14.56±0.15	14.71±0.39	4.63±0.17	44.38±0.34	27.75±0.31	34.70±0.21	31.18±0.11	18.76±0.15	22.40±0.15
	L-Threonine	0.72±0.01	1.31±0.03	1.25±0.03	3.03±0.02	1.03±0.02	1.44±0.01	2.72±0.01	1.94±0.01	2.03±0.02
	L-Valine	0.12±0.01	0.29±0.01	0.24±0.01	1.04±0.01	0.16±0.01	0.33±0.01	1.87±0.01	0.80±0.01	0.27±0.01
	Phenylalanine	0.19±0.01	0.71±0.02	0.42±0.01	1.15±0.01	0.43±0.01	0.71±0.01	1.80±0.01	0.95±0.01	0.51±0.01
	Serine	0.91±0.01	1.20±0.03	0.62±0.01	1.88±0.02	1.35±0.01	2.81±0.02	1.82±0.01	1.02±0.01	1.72±0.02
Sugars	D-(-)-Fructose	10.35±0.01	20.07±0.98	19.05±0.01	2.48±0.01	7.58±0.01	7.42±0.01	65.99±0.20	98.13±0.85	66.15±0.03
	D-(+)-Turanose	0.19±0.01	0.54±0.01	0.18±0.01	0.25±0.01	0.18±0.01	0.14±0.01	0.07±0.01	0.44±0.01	---
	D-Galactose	0.15±0.01	0.41±0.02	0.33±0.01	0.09±0.01	0.27±0.01	---	0.07±0.01	0.68±0.01	0.36±0.01
	D-Glucopyranoside	1.68±0.03	4.13±0.07	4.34±0.09	1.91±0.02	1.30±0.07	1.28±0.01	4.14±0.01	6.89±0.06	7.80±0.05
	D-Glucose	11.10±0.74	39.20±1.71	22.28±0.22	5.02±0.03	8.79±0.50	13.09±0.05	0.12±0.01	150.36±1.29	113.03±0.21
	D-Ribose	0.32±0.01	---	0.19±0.01	---	0.24±0.01	---	0.43±0.01	0.44±0.01	0.45±0.01
	Galactinol	0.22±0.01	0.37±0.01	---	0.23±0.01	0.19±0.01	0.34±0.01	0.44±0.01	---	0.14±0.01
	Maltose	0.17±0.01	---	---	---	---	---	0.10±0.01	---	---
	Melibiose	0.32±0.01	1.37±0.02	0.26±0.01	0.48±0.01	0.52±0.02	0.32±0.01	1.32±0.01	1.02±0.01	---
	Sucrose	45.58±0.35	75.92±0.95	28.61±0.17	97.25±0.24	63.97±0.28	110.47±0.79	94.16±0.61	73.59±0.87	87.34±0.10

**Krebs cycle intermediates**	Shikimic acid	0.16±0.01	0.35±0.01	---	---	---	---	0.26±0.01	---	---
	Citric acid	7.72±0.05	9.79±0.13	2.62±0.07	1.93±0.01	3.75±0.01	8.00±0.06	1.53±0.01	0.88±0.01	1.54±0.02
	2-Ketoglutaric acid	0.42±0.01	0.24±0.01	0.27±0.01	0.24±0.01	0.26±0.01	0.44±0.01	0.24±0.01	0.30±0.01	0.44±0.01
	D-Psicose	14.84±0.01	25.41±1.20	22.53±0.23	---	10.49±0.01	10.44±0.04	---	121.96±0.06	88.22±0.27
	Chlorogenic acid	0.11±0.01	0.55±0.03	0.62±0.03	0.30±0.01	0.11±0.01	0.15±0.01	0.06±0.01	---	0.68±0.01
	Malic acid	62.61±0.23	81.71±2.84	83.38±3.62	28.28±0.16	27.45±0.37	48.91±0.27	56.08±0.14	115.81±0.98	55.65±0.52
**Sugar acids**	Glyceric acid	3.68±0.06	5.29±0.22	1.44±0.03	1.83±0.01	2.19±0.06	2.51±0.02	5.08±0.01	1.57±0.01	4.86±0.04
	L-Threonic acid	12.37±0.20	16.51±0.64	5.76±0.20	5.53±0.03	7.14±0.30	11.71±0.06	40.00±0.05	8.39±0.07	8.65±0.08
	Ribonic acid	0.10±0.01	0.39±0.01	---	---	0.33±0.01	0.12±0.01	0.17±0.01	---	0.07±0.01
**Fatty acids**	4-Aminobutanoic acid	1.57±0.02	1.19±0.02	0.34±0.01	0.40±0.01	0.55±0.01	1.06±0.01	2.75±0.01	1.17±0.01	0.86±0.01
	Glycerol	1.44±0.06	1.56±0.01	1.00±0.01	1.39±0.01	0.90±0.03	0.98±0.01	0.55±0.01	1.10±0.01	3.84±0.01
	Myo-Inositol	20.73±0.22	34.20±0.80	15.31±0.53	34.62±0.24	33.54±0.29	52.82±0.37	33.68±0.12	42.76±0.38	53.78±0.06
	Palmitic Acid	0.25±0.01	0.19±0.01	0.48±0.01	0.26±0.01	0.17±0.01	0.33±0.02	0.12±0.01	0.39±0.02	0.11±0.01
	Stearic acid	0.26±0.01	0.12±0.01	0.65±0.01	0.40±0.01	0.24±0.01	0.39±0.06	---	---	---
**Miscellaneous**	2-Butenedioic acid	0.32±0.01	0.65±0.01	0.69±0.01	0.47±0.01	0.21±0.01	0.24±0.01	0.26±0.01	0.95±0.01	0.39±0.01
	Butanedioic acid	0.64±0.01	0.45±0.01	0.19±0.01	0.11±0.01	0.20±0.01	0.35±0.01	0.16±0.01	0.13±0.01	0.13±0.01
	Ethanolamine	0.95±0.02	1.52±0.05	0.85±0.01	1.21±0.01	0.79±0.01	0.93±0.01	0.73±0.01	1.40±0.01	1.03±0.01
	Quininic acid	9.96±0.18	5.42±0.12	1.75±0.03	2.69±0.01	4.37±0.07	10.56±0.05	19.47±0.04	1.34±0.01	8.02±0.04
	Silanol	0.78±0.01	1.83±0.01	2.13±0.01	---	0.52±0.01	0.93±0.01	1.26±0.01	0.66±0.01	---
**Amine**	Tyramine	0.32±0.01	0.28±0.01	0.22±0.01	0.25±0.01	0.11±0.01	0.23±0.01	0.72±0.06	0.14±0.01	0.14±0.01
	2-Propenoic acid	0.09±0.01	---	---	---	---	---	---	---	---
	Cyclooctasiloxane	0.85±0.01	0.34±0.01	0.29±0.01	0.25±0.01	0.22±0.01	0.47±0.01	0.18±0.01	0.41±0.01	0.25±0.01

Note: “---” means not detected

Table S3: Identified different metabolites in the leaf of WT and two selected transgenic lines (L20 and L40) under salt (200 mmol L^{-1} NaCl) and drought (10% PEG) treatment for 24 hr under elevated CO_2 (900 ppm) condition. The relative concentration of each group of metabolites in control and stress condition is a mean of data from two biological replicates.

Categorises	Metabolites	Control			Salt (200 mmol L^{-1} NaCl)			Drought (10% PEG)		
		WT	L20	L40	WT	L20	L40	WT	L20	L40
		Metabolites Concentration (μg/g) FW								
Amino acids	Adenine	0.18±0.01	11.78±0.18	0.08±0.01	0.27±0.01	---	0.28±0.01	0.13±0.01	0.14±0.01	0.11±0.01
	Asparagine	0.13±0.01	230.58±0.19	2.61±0.01	1.92±0.02	2.78±0.03	2.93±0.18	0.55±0.01	2.27±0.09	0.70±0.01
	Glycine	0.23±0.01	15.28±0.08	0.26±0.01	0.35±0.01	0.34±0.01	0.19±0.01	0.23±0.01	0.34±0.01	0.34±0.01
	L-Aspartic acid	5.76±0.03	413.29±4.74	6.58±0.01	5.92±0.01	7.73±0.02	0.13±0.01	5.35±0.01	9.15±0.02	7.37±0.06
	L-Glutamic acid	5.33±0.05	314.22±2.89	5.12±0.01	7.91±0.01	5.49±0.02	4.42±0.09	4.03±0.01	3.68±0.01	4.82±0.04
	L-Glutamine	0.44±0.01	198.88±0.62	2.05±0.01	1.40±0.01	2.76±0.02	12.96±0.32	1.77±0.01	5.95±0.06	2.31±0.03
	L-Leucine	---	---	---	1.41±0.01	0.96±0.01	0.39±0.03	---	0.39±0.01	0.30±0.01
	L-Lysine	---	15.75±0.08	---	0.23±0.01	0.23±0.01	0.27±0.01	---	0.65±0.01	---
	L-Proline	8.44±0.06	10.19±0.02	5.65±0.01	28.95±0.03	17.43±0.07	18.60±1.11	9.74±0.02	16.96±0.07	11.85±0.12
	L-Serine	---	4.18±0.12	0.06±0.01	0.20±0.01	0.16±0.01	0.09±0.01	0.08±0.01	0.08±0.01	0.12±0.01
	L-Threonine	1.08±0.01	42.65±0.41	1.02±0.01	1.80±0.01	1.49±0.01	1.06±0.08	0.94±0.01	1.17±0.01	1.37±0.01
	L-Tryptophan	0.70±0.02	14.43±0.01	0.06±0.01	0.41±0.01	0.09±0.01	---	0.30±0.01	0.04±0.01	0.06±0.01
	L-Valine	0.23±0.01	8.20±0.04	0.22±0.01	1.17±0.01	0.61±0.01	0.45±0.01	0.28±0.01	0.30±0.01	0.40±0.01
	Methylalanine	---	6.23±0.03	---	---	---	---	---	---	0.30±0.04
	Phenylalanine	0.64±0.01	24.21±0.49	0.63±0.01	2.22±0.01	1.08±0.01	0.47±0.03	0.83±0.01	0.65±0.01	0.84±0.01
	Serine	0.98±0.01	93.18±0.68	1.29±0.01	3.02±0.01	2.18±0.01	1.55±0.12	1.45±0.01	1.56±0.01	1.83±0.01
	β-Alanine	---	---	0.05±0.01	---	0.08±0.01	0.08±0.01	0.07±0.01	0.11±0.01	0.06±0.01

**Sugars**	D-(-)-Fructose	14.71±0.01	187.24±0.09	20.22±0.01	46.36±0.20	27.94±0.06	36.86±0.60	28.63±0.04	17.06±0.06	14.53±0.01
	D-Galactose	3.24±0.04	---	5.42±0.01	---	8.86±0.01	---	---	---	1.21±0.11
	D-Glucose	19.70±0.18	15.04±0.03	30.05±0.01	76.96±0.31	47.76±0.11	57.78±0.03	36.20±0.11	25.30±0.25	14.64±0.04
	L-(+)-Threose	0.32±0.01	213.08±0.85	0.18±0.01	0.12±0.01	---	---	0.12±0.01	---	---
	Melibiose	---	---	0.19±0.01	0.33±0.01	---	---	0.13±0.01	---	0.34±0.01
	Sucrose	66.86±0.63	211.83±0.67	34.95±0.11	126.05±1.24	86.93±0.26	89.38±1.60	54.80±0.08	71.38±0.69	45.87±0.28
**Sugar acid**	L-Threonic acid	0.36±0.01	571.51±6.70	7.31±0.01	0.27±0.01	5.01±0.01	8.12±0.26	3.48±0.33	4.13±0.03	8.40±0.04
	Glycerol	5.89±0.01	149.21±5.07	2.59±0.05	3.84±0.01	2.97±0.07	2.79±0.01	3.58±0.04	1.52±0.04	3.37±0.01
**Krebs cycle intermediates**	Malic acid	93.90±0.86	106.63±1.29	27.83±0.03	66.98±0.09	22.05±0.05	38.83±1.89	70.77±0.19	27.19±0.25	60.29±0.50
	Citric acid	17.17±0.25	---	3.64±0.02	11.38±0.02	2.97±0.01	2.69±0.03	9.02±0.06	8.03±0.05	8.81±0.13
	2-Ketoglutaric acid	0.22±0.01	268.59±1.07	0.12±0.01	0.24±0.01	0.13±0.01	0.14±0.01	0.14±0.01	0.10±0.01	0.13±0.01
	Shikimic acid	---	4.96±0.01	0.06±0.01	0.25±0.01	---	0.09±0.09	0.11±0.01	0.07±0.01	0.17±0.01
**Fatty acid**	4-Aminobutanoic acid	1.42±0.01	43.89±0.72	0.73±0.01	1.53±0.01	0.66±0.01	0.79±0.04	1.11±0.01	1.04±0.01	1.24±0.01
**Amine**	Tyramine	0.35±0.01	26.35±0.20	0.58±0.01	1.72±0.01	0.73±0.01	0.13±0.01	0.21±0.01	0.44±0.03	0.60±0.04
**Miscellaneous**	Ethanolamine	0.78±0.01	22.47±0.25	0.34±0.01	0.52±0.01	0.56±0.01	0.30±0.01	0.79±0.01	0.31±0.01	0.73±0.01
	Quininic acid	5.85±0.06	---	2.65±0.01	19.01±0.07	2.32±0.01	5.39±0.22	3.44±0.01	3.31±0.03	2.46±0.01

Note: “---” means not detected

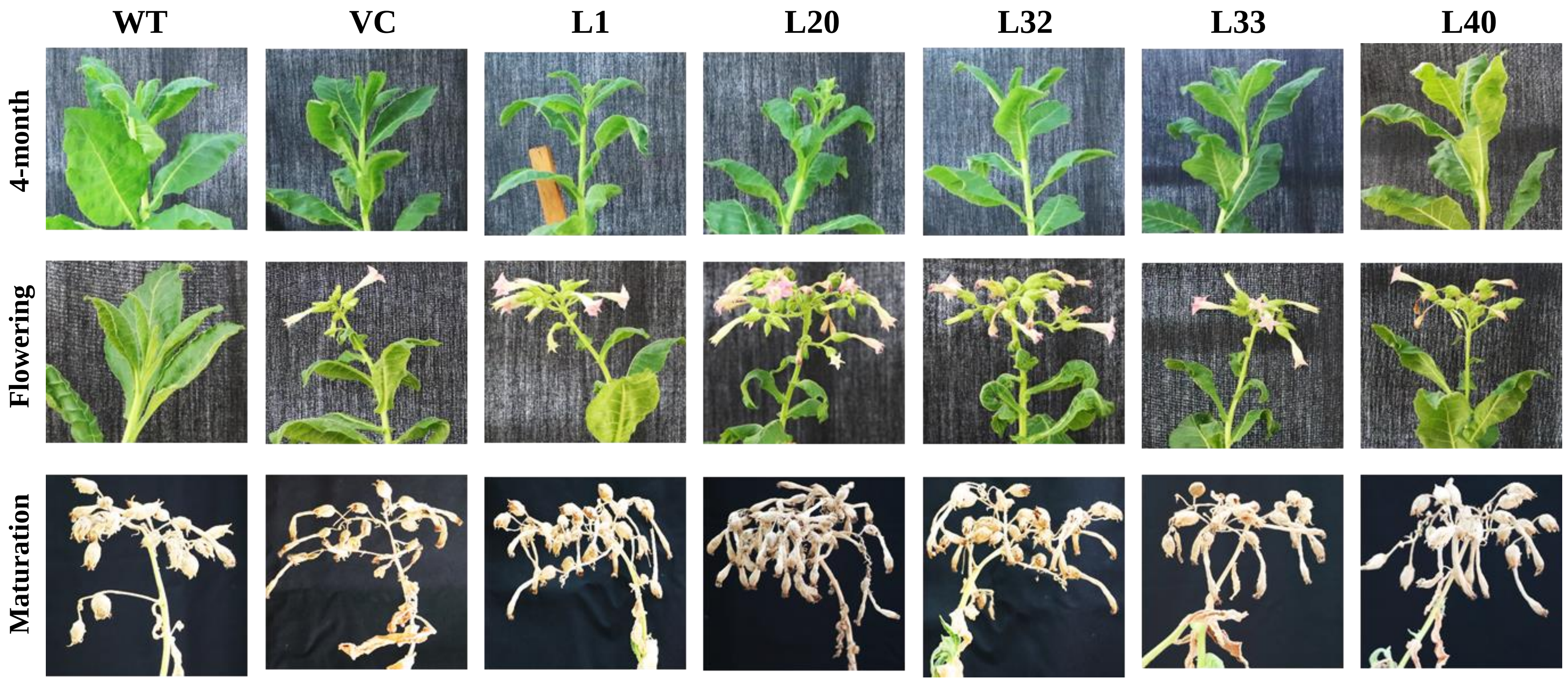

Figure S19: Comparative plant growth study. Plant images of WT, VC, and T1 transgenic lines (L1, L20, L32, L33 and L40) were documented at different stages of growth

Table S4: Performance of plants grown in green house under normal condition

**Characteristics**	**WT**	**VC**	**L1**	**L20**	**L32**	**L33**	**L40**
Plant height (inch)	48 ± 1	44 ± 2	42 ± 3	47 ± 5	41 ± 1	42 ± 6	42 ± 5
No. of leaf / plant	37 ± 2	37 ± 1	39 ± 6	40 ± 5	45 ± 1	43 ± 1	37 ± 1
No. of pod / plant	19 ± 1	13 ± 2	25 ± 6	13 ± 2	16 ± 1	20 ± 1	20 ± 4
Pod weight / plant (g)	2.2 ± 0.2	1.2 ± 0.1	3 ± 1	1.3 ± 0.1	2.1 ± 0.4	2.1 ± 0.3	2.4 ± 0.5
Plant dry weight (g)	20 ± 1	23 ± 3	24 ± 4	29 ± 2	28 ± 2	25 ± 2	22 ± 3

Value showed as mean ± SE (n=3)

Table S5: Analysis of the photosynthesis parameters of transgenic lines grown in ambient CO_2 environment under control field conditions

Transgenic Lines ↓	Net photosynthesis		Conductance		Ci		ETR		Transpiration rate		VpdL		Ci/Ca		qP		ΦPSII	
	Mean	±SE	Mean	±SE	Mean	±SE	Mean	±SE	Mean	±SE	Mean	±SE	Mean	±SE	Mean	±SE	Mean	±SE
**Three month old plants**																		
WT	20.09396469	0.1958288	0.10517	0.00607	63.7294	20.9328	58.3473	11.5159	1.736456034	0.07847	1.63463	0.01927	0.16782	0.05501	0.24131	0.03494	0.13352	0.02623
VC	15.02514502	0.7954501	0.07324	0.00295	47.4017	32.0056	54.0696	11.8856	1.250340431	0.04573	1.68291	0.00576	0.12168	0.08157	0.24179	0.05181	0.1237	0.0272
L1	23.79635317	0.6854316	0.1265	0.00841	63.7389	11.2833	68.8984	8.25011	2.161053811	0.12521	1.69503	0.01367	0.16981	0.03013	0.31163	0.0308	0.15731	0.01887
L20	21.5123605	0.0777362	0.10623	0.00145	45.2469	5.96018	52.7375	1.8512	1.704594207	0.02128	1.58808	0.00165	0.11964	0.01563	0.22793	0.01035	0.12056	0.00423
L32	20.85913117	0.2422805	0.10216	0.0028	42.3449	5.32899	66.2255	12.0022	1.682731136	0.0427	1.62907	0.0028	0.11201	0.01397	0.28676	0.0468	0.15154	0.02746
L33	20.05651331	0.1655738	0.10331	0.00095	59.7099	0.33412	71.666	0.9462	1.688275392	0.01271	1.61551	0.00256	0.15749	0.00087	0.29048	0.00364	0.16386	0.00218
L40	20.00376976	0.2188592	0.09827	5.7E-05	45.127	3.91353	62.6355	3.47971	1.682807957	0.00312	1.69292	0.00215	0.11884	0.01027	0.25681	0.01118	0.14321	0.00822
**Four month old plants**																		
WT	18.99692909	0.7888478	0.09594	0.01363	48.8322	30.8618	57.9941	11.0261	1.583388597	0.18134	1.63853	0.04206	0.12878	0.08123	0.25372	0.05152	0.13262	0.02518
VC	15.93553916	0.1596335	0.07541	0.00147	32.9824	8.07949	58.1834	11.4107	1.281902634	0.02359	1.67586	0.00163	0.08707	0.0217	0.26496	0.03956	0.13314	0.02609
L1	24.47664854	0.5695839	0.14673	0.00455	98.2851	1.60517	61.3909	1.28913	2.450825576	0.05972	1.65935	0.01037	0.26187	0.00451	0.28659	0.00718	0.14024	0.00297
L20	21.60079464	0.0577014	0.11403	0.00182	66.1521	5.68292	51.6572	16.3959	1.821132284	0.0266	1.58165	0.00186	0.17486	0.01509	0.2143	0.0692	0.11812	0.03747
L32	20.87305452	0.166625	0.10904	0.00161	63.889	7.07169	82.9041	18.4311	1.791934528	0.02735	1.62638	0.00102	0.16842	0.01857	0.34029	0.06929	0.18964	0.04216
L33	20.15271022	0.6168917	0.10085	0.00078	50.8635	12.7591	84.5235	9.62182	1.659140272	0.00927	1.62591	0.0033	0.13401	0.03344	0.35064	0.03369	0.19324	0.022
L40	20.07979493	0.4207629	0.09976	0.00146	48.4886	2.57241	57.8953	1.98466	1.712597435	0.02409	1.69752	0.00073	0.12776	0.00662	0.23279	0.00092	0.1319	0.00463
**Before flowering**																		
WT	4.461306896	0.0967034	0.03995	0.00077	208.26	0.35492	41.1851	2.78472	0.721641015	0.02299	1.78118	0.02547	0.55064	0.00051	0.21239	0.01359	0.09405	0.00628
VC	4.577505929	0.0656195	0.03405	0.00297	170.774	16.7646	38.1122	0.33788	0.663634634	0.03601	1.92455	0.06253	0.41885	0.0545	0.19454	0.00362	0.0871	0.00081
L1	10.78551153	0.0858307	0.05507	0.00019	65.108	1.03034	61.662	3.76483	1.237885612	0.00361	2.14183	0.00381	0.16697	0.00237	0.30148	0.01712	0.14083	0.00858
L20	11.33906393	0.3020193	0.05778	0.00172	63.8019	0.7873	73.4989	2.53057	1.308683394	0.04064	2.19537	0.00294	0.1648	0.00207	0.35295	0.01301	0.16783	0.00574
L32	10.76971343	0.0334883	0.0543	0.00037	61.9523	2.28446	75.6423	11.0428	1.217138155	0.01557	2.13782	0.01302	0.1589	0.00535	0.35345	0.03948	0.17275	0.02524
L33	6.712986841	0.2508395	0.03476	3.4E-05	78.7368	10.7562	31.3104	2.7413	0.643953183	0.00303	1.81542	0.00688	0.19894	0.02753	0.15828	0.01218	0.07162	0.00629
L40	6.310659956	1.1509911	0.03342	1.9E-05	85.9713	54.692	31.4504	3.3061	0.626835642	0.00445	1.84029	0.01626	0.21632	0.13729	0.1596	0.01946	0.07185	0.00753

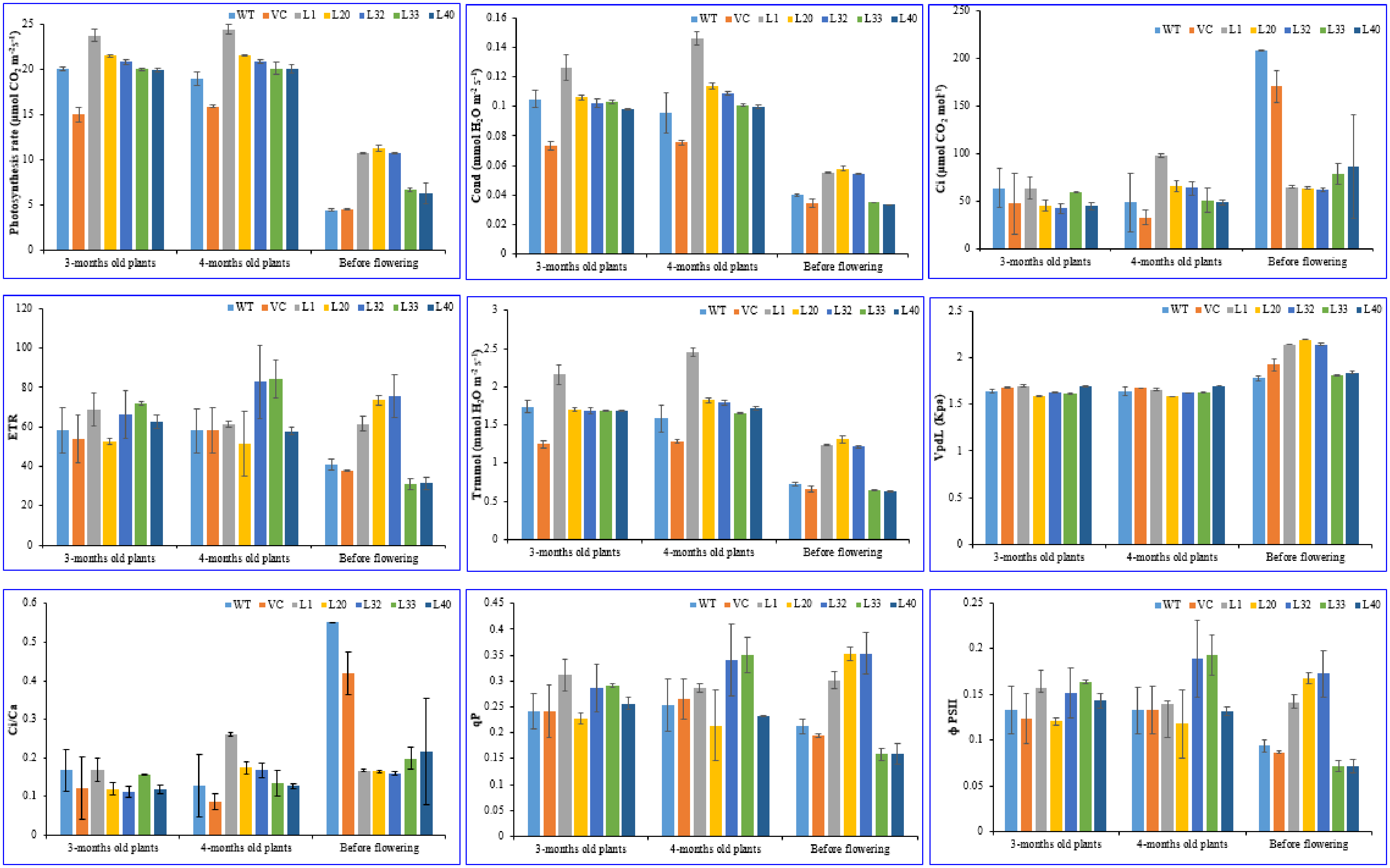

Supplement: Supplementary file 1 [file Data_Sheet_1.PDF]
